# Design, synthesis, herbicidal activity, and the molecular docking study of novel phenylpyrazole derivatives with strobilurin moieties†

**DOI:** 10.1039/d5ra02377g

**Published:** 2025-05-14

**Authors:** Wenliang Zhang, Xiaodong Jin, Wenwu Chen, Shulin Hao, Xiaohua Du

**Affiliations:** a Catalytic Hydrogenation Research Center, Zhejiang Key Laboratory of Green Pesticides and Cleaner Production Technology, Zhejiang Green Pesticide Collaborative Innovation Center, Zhejiang University of Technology Hangzhou 310014 Zhejiang China duxiaohua@zjut.edu.cn; b Taizhou Key Laboratory of Green Agrochemicals Development, Synwill Co., Ltd Taizhou 318000 Zhejiang China; c Zhejiang Hanghua Technology Co., Ltd Huzhou 313000 China

## Abstract

Phenylpyrazole derivatives were innovatively designed and synthesized to fabricate novel herbicides containing strobilurin moieties. These resulting compounds were analyzed by nuclear magnetic resonance spectroscopy (NMR) and high-resolution mass spectrometry (HRMS), exhibiting high herbicidal properties against Amaranthus retroflexus. The target compounds were synthesized through cyclization, chlorination, Vilsmeier–Hack reaction, Suzuki coupling reaction, and esterification reactions. The experimental conditions were mild, and the starting materials were easy to obtain, with a separation yield of approximately 80%. Moreover, herbicidal activity of the title compounds was studied *via* a pot culture experiment. Compounds 7a, 7b, 7e–7g, 7i, and 7l demonstrated good inhibition on *A*. *retroflexus*, which was inferior to that of fomesafen at 150 g a.i./hm^2^. The docking results indicated that the binding energies of compound 7f with protoporphyrinogen oxidase (PPO) were both negative and spontaneous, with numerous interaction active sites. Thus, it is speculated that compound 7f is a PPO inhibitor.

## Abstract 2

Phenylpyrazole derivatives were designed and synthesized to fabricate novel herbicides containing strobilurin moieties. Most compounds demonstrated good inhibition on *A. retroflexus*, which were speculated as PPO inhibitors.

## Graphical abstract

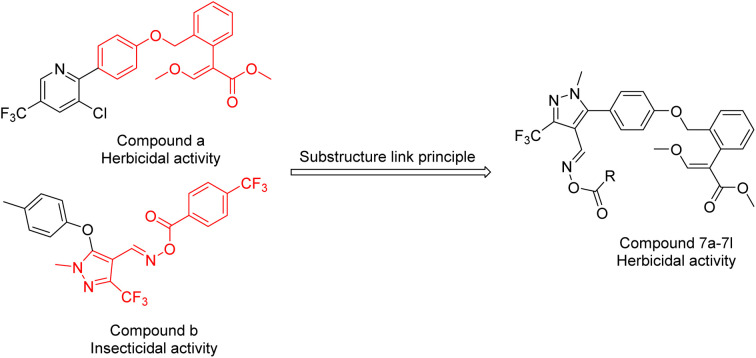

## 1 Introduction

Pesticides are an important means of preventing and controlling agricultural diseases, insects, and weeds and ensuring agricultural production.^1^ However, some pesticides have been reduced in use or even banned due to the development of resistance or their high biological toxicity.^2^ Penoxsulam is a herbicide widely used in rice fields, but an increasing number of weeds in China have developed biological resistance to it, particularly *Echinochloa crusgalli*, which exhibits the highest level of resistance. In the past, fipronil was widely used due to its excellent insecticidal activity but subsequently proved to be highly toxic to bees, and its market share was gradually replaced by chlorantraniliprole. The structure of pesticides needs to be constantly improved to combat the increasing problem of biological resistance. Therefore, discovering new pesticides is one of the important research directions of the pesticide industry.^3–5^

**Fig. 1:**
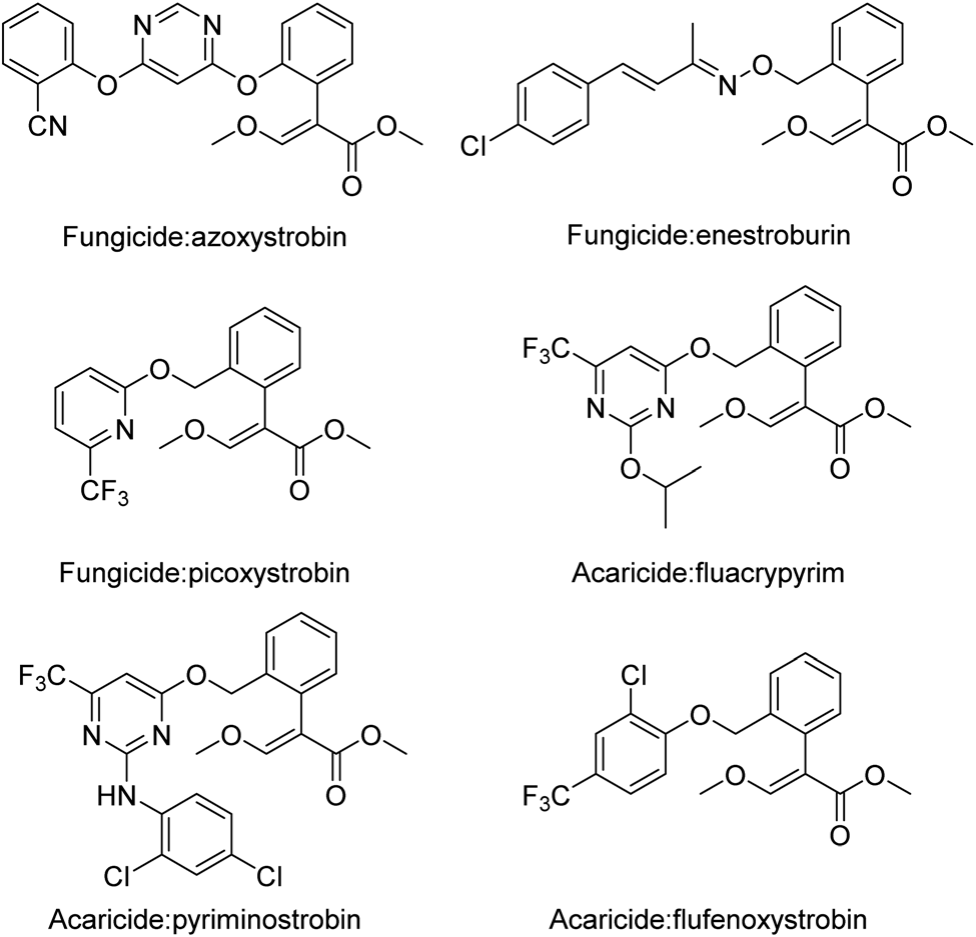
Some biologically active compounds containing strobilurin moieties.

As important natural antifungal compounds isolated from mushrooms, strobilurin derivatives have received much more attention in pesticide creation projects.^6^ These derivatives exhibit biological activity by inhibiting mitochondrial respiration. In the field of fungicides, such as azoxystrobin, the first strobilurin fungicide was discovered and developed by Zeneca Agrochemicals (Syngenta AG).^7^ Owing to their excellent biological activity, more strobilurin derivatives, such as fungicides,^8–10^ herbicides,^11,12^ and acaricides,^13–16^ have been developed and reported (Fig. 1).

**Fig. 2:**
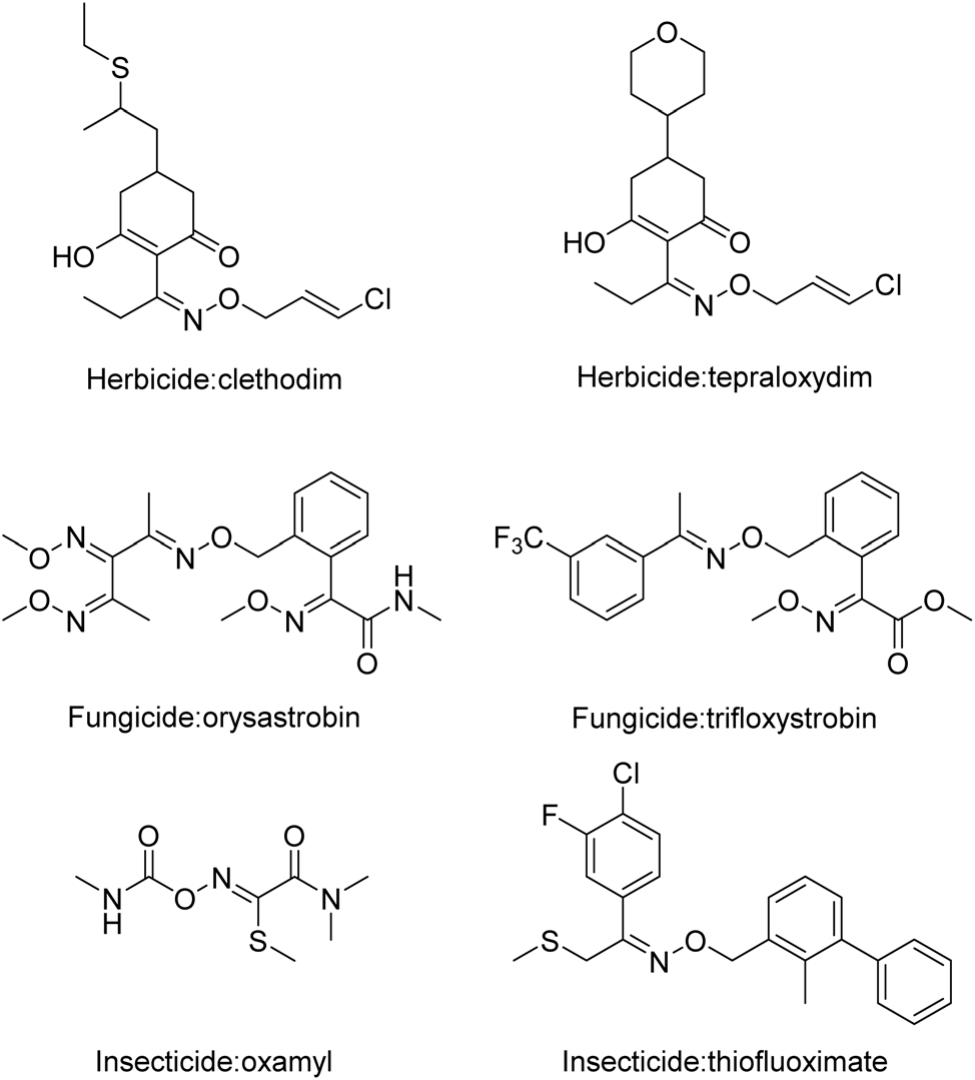
Some biologically active compounds containing oxime moieties.

Moreover, oxime derivatives are very common in the field of pesticides, with advantages such as high yields and mild reaction conditions.^17^ Many pesticides containing oxime-based compounds exhibit excellent biological activity. Herbicides,^18,19^ fungicides,^20,21^ and insecticides^22,23^ composed of oxime have been frequently reported (Fig. 2). Agricultural mites are one of the most difficult biological groups to control worldwide. Fenpyroximate, composed of pyrazole oxime derivatives,^24^ a commercial acaricide created by Nihon Nohyaku, has been widely used to increase crop yields.

PPO (EC1.3.3.4) is widely present in organisms and is a biological enzyme that assists in the synthesis of chlorophyll or heme, which promotes the oxidation of protoporphyrinogen IX to proto-porphyrin IX. A large amount of protoporphyrin IX can accumulate by suppressing the performance of PPO. PPO inhibitors replace protoporphyrinogen IX in binding to PPO enzymes, resulting in a large accumulation of the substrate protoporphyrinogen IX and permeation into the cytoplasm, which is subsequently oxidized to protoporphyrin IX by nonenzymatic mechanisms or oxidases. Reactive oxygen species are produced in the presence of visible light, ultimately leading to plant death. Many PPO inhibitor compounds with herbicidal activity (such as oxadiazole, uracil, phenylpyrazole, diphenyl ether, thiadiazole, triazolinone, oxazolidinone, and *N*-phenylphthalimide^25–31^) have been reported. Therefore, the PPO enzyme has become an important target in the design of herbicides. The composition and structure of proteins are the basis of their functional performance, which to some extent determines their biological functions. The PPO protein from *Nicotiana tabacum* is formed by the dimerization of two monomers with a mass of *ca.* 55 kDa, with each monomer containing 503 amino acids.^32^ The 503 amino acids of PPO fold into a compact structure with three domains: an FAD-binding domain, a substrate-binding domain, and a membrane-binding domain. The first two domains display a *p*-hydroxybenzoate-hydroxylase (PHBH)-fold-like topology. The parts of the structure re-sponsible for FAD binding show significant amino acid (aa) sequence and structural homologies to other flavoenzymes, such as human monoamine oxidase B (MAO B, 1.68 A° rmsd over 256 Ca atoms with 15.1% aa sequence identity), polyamine oxidase (PAO, 1.71 A° rmsd over 190 Ca atoms with 14.9% aa sequence identity), and d-amino acid oxidase (DAO, 1.98 A° rmsd over 148 Ca atoms with 14.9% aa sequence identity). Furthermore, phytoene desaturase (PDS) is a plant enzyme involved in carotenoid biosynthesis, with 15.2% aa sequence identity.

**Scheme 1:**
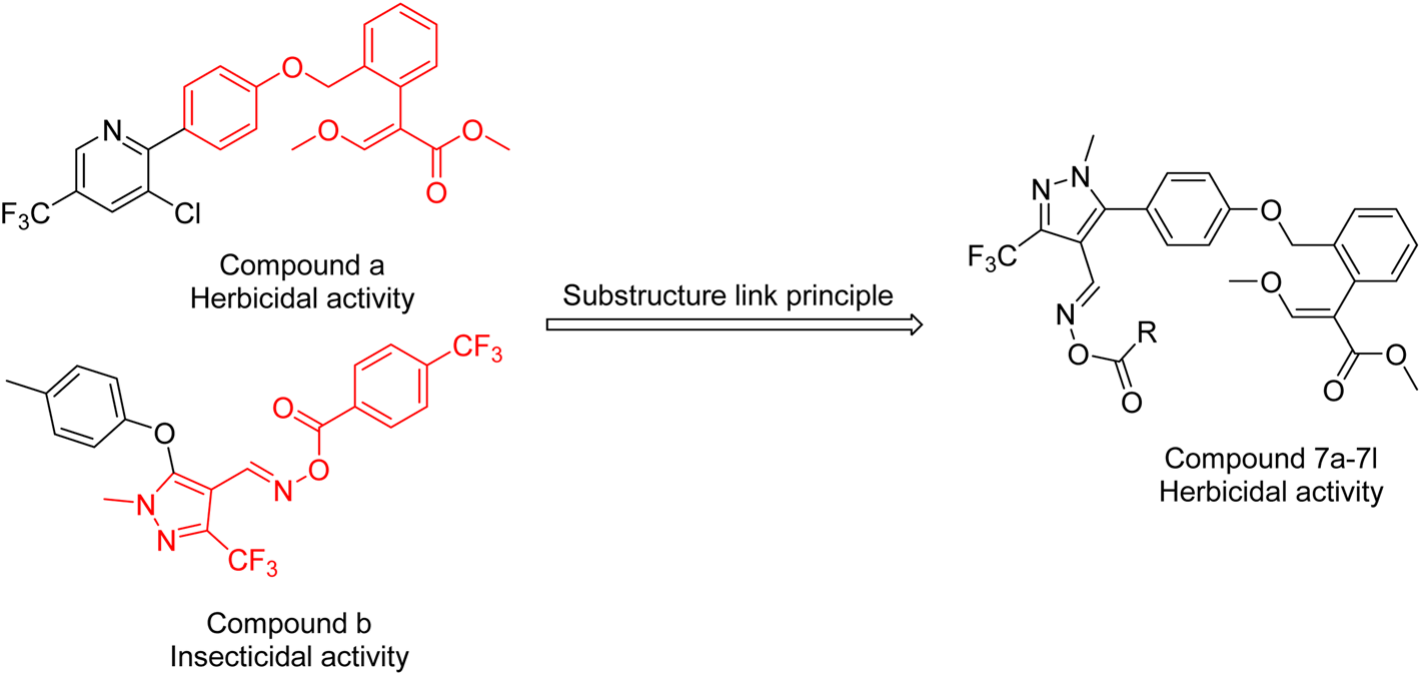
Design strategy for the novel pyrazoxime derivatives.

Therefore, strobilurin derivatives and oxime derivatives have generally been used in pesticides.^33–38^ Liu *et al.* designed and synthesized a class of novel phenylpyrazole derivatives, some of which possessed good fungicidal and acaricidal activities.^39^ A series of strobilurin derivatives were prepared by Cao *et al.* to treat weeds at a very low dose. These compounds not only can protect against different kinds of weeds but also have less influence on crops.^11,12^ A series of new *N*-methoxycarbamate derivatives containing substituted pyrazoles were synthesized using substituted aryl alkyl ketones as the starting material by esterification, cyclization, and condensation, and these products showed good fungicidal activity.^40^ To obtain bioactive compounds with new structures, the absorption distribution metabolism and exclusion of compound a (ESI Material Fig. S1†) were analysed, and the pyridine was replaced with a pyrazole. The structure of these compounds was optimized, and their herbicidal activity was investigated. Twelve pyrazoxime derivative compounds containing strobilurin motifs were prepared according to the following principles: “substructure link principle” (Scheme 1).^41–44^ The title products were explored *via* NMR, IR and HRMS (ESI Material Fig. S2–S64†). In addition, the herbicidal performance of the resulting products was determined.

## 2 Results and discussion

### 2.1 Chemistry

**Scheme 2:**
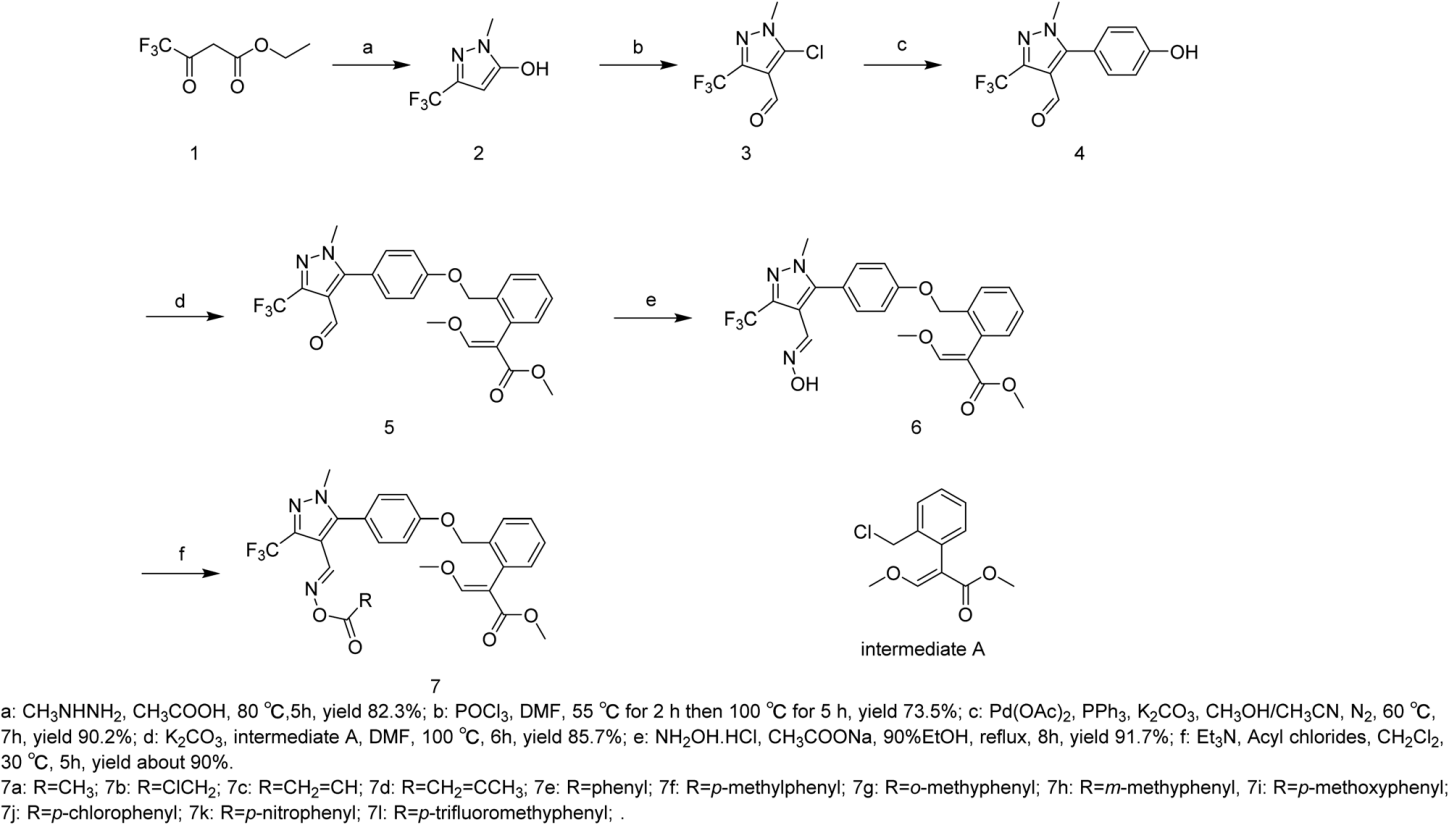
Synthesis of phenylpyrazole derivatives.

Scheme 2 displays the preparation processes of the compounds. Compound 4 was fabricated by cyclization, chlorination, the Vilsmeier–Haack reaction, and the Suzuki coupling reaction using ethyl 4,4,4-trifluoro-3-oxobutanoate (1) and methylhydrazine as the starting materials. Compound 6 was synthesized in two steps, namely, etherification and oximation. The high-yield target compounds were generated *via* esterification, where acyl chlorides and triethylamine (TEA) served as the reactants and dichloromethane (DCM) acted as the solvent. Intermediate A was synthesized following established procedures,^7^ with its E-configuration confirmed through comparison to literature data.^45,46^ Compounds 7a–7l were characterized *via* HRMS and NMR, and the results agreed with the results of the assigned structures (ESI Materials†).

### 2.2 Greenhouse herbicidal performance testing

As listed in Table 1, three preliminary conclusions have been drawn. Almost all the compounds exhibited good activity against *A*. *retroflexus*. First, when the ester is an alkene ester, its activity is the lowest among all the tested compounds. Second, when the substituent at position 4 of the benzene ring was methyl, the compound exhibited the highest herbicidal activity. Third, the location of the substituents on the benzene ring has a minor impact on the activity of the compounds.

**Table 1:** The chemical composition and herbicidal performance of phenylpyrazole derivatives subjected to 150 g a.i./hm^2^ postemergence treatment in a greenhouse (25 ± 1 °C) experimental environment^a^

Compound	Chemical structure	Fresh weight inhibition rate (%)					
	R	EC	DS	SV	AT	AR	EP
7a	CH_3_	0.0	13.2	7.5	8.5	79.3	5.5
7b	CH_2_Cl	20.2	5.7	17.2	15.3	84.1	7.8
7c	CH_2_ = CH	13.4	18.5	9.5	11.2	64.5	6.7
7d	CH_2_ = CCH_3_	5.3	21.2	22.3	16.3	60.3	15.6
7e	Phenyl	23.9	12.7	10.2	4.5	82.3	5.2
7f	*p*-methylphenyl	15.8	0.0	5.8	23.2	89.0	0.0
7g	*o*-methylphenyl	0.0	25.2	25.2	5.7	78.0	11.2
7h	*m*-methylphenyl	9.4	22.3	26.3	7.2	73.5	12.3
7i	*p*-methoxyphenyl	25.6	0.0	13.1	45.6	78.7	7.3
7j	*p*-chlorophenyl	11.6	13.5	0.0	0.0	76.3	0.0
7k	*p*-nitrophenyl	3.3	18.2	0.0	10.2	74.5	0.0
7l	*p*-trifluoromethylphenyl	6.3	10.2	3.2	0.0	78.3	0.0
CK	—	0.0	0.0	0.0	0.0	0.0	0.0
Fomesafen	—	57.8	84.5	6.5	95.1	92.3	71.3

a Fresh weight inhibition rate (%): (control group fresh weight – treatment group fresh weight)/control group fresh weight × 100%; EC: *Echinochloa crusgalli*; DS: *Digitaria sanguinalis*; SV: *Setaria viridis*; AT: *Abutilon theophrasti*; AR: *Amaranthus retroflexus*; EP: *Eclipta prostrate*. CK: controlcheck.

### 2.3 Determination of ED_50_

To gain a deeper understanding of the herbicidal activity of compounds 7b, 7e, and 7f, an ED_50_ assay was subsequently conducted. As shown in Table 2, compounds 7b and 7f exhibited good herbicidal activity against *A. retroflexus*, with respective ED_50_ values of 13.1958 and 12.5696 g a.i./hm^2^, which were comparable to that of the positive control (ED_50_ of 10.6720 g a.i./hm^2^).

**Table 2:** Herbicidal activities of compounds 7b, 7e and 7f against *A*. *retroflexus* in a greenhouse setting

Compound	*A. retroflexus*
	ED_50_ (g a.i./hm^2^)
7b	13.1958
7e	37.0742
7f	12.5696
Fomesafen	10.6720

### 2.4 Docking determination

In this study, docking studies were conducted to examine the binding configuration between the small molecule and the protein (PDB ID: 1SEZ) using the AutoDock Vina program. The structural formula of the substrate molecule was drawn using ChemDraw, and its energy was optimized and saved as a PDB format file using Chem3D. AutoDock Tools 1.5.6 was employed to add charges and perform other processing on the small molecule's PDB file, which was then saved in PDBQT format. Using the graphical interface of AutoDock Tools 1.5.6, polar hydrogen atoms and charges were added to the protein, converting its PDB format file into PDBQT format. The protein molecules were set as the search space, and an appropriate grid was established in the *x*, *y*, and *z*-axis directions with an exhaustiveness of 8. Finally, all remaining parameters in the AutoDock software are set to their default values. Once docking was complete, we analyzed 200 docking results through cluster analysis. From the optimal cluster, we will identify the binding conformation with the best docking score (the lowest value) to elucidate the binding site and mode between the template molecule and the screening molecule.^47^ Fomesafen and compound 7f were selected for molecular docking to analyze the interaction types of this series of products with PPO.

First, the binding energies of fomesafen and compound 7f with PPO proteins were tested. As shown in Table 3, the binding energies of fomesafen and compound 7f with proteins are negative, indicating that the binding between proteins and small molecules is spontaneous. In addition, these proteins have high binding energies with small molecules, and the close binding between compounds and proteins provides a basis for their herbicidal activity.

**Table 3:** Binding energy between different small molecules and proteins after docking

Compound	Binding energy (kcal mol^−1^)
7f	−8.073
Fomesafen	−9.38

According to the results of molecular docking, fomesafen formed van der Waals forces with the amino acid residues LEU B:369, LEU B:473, ASN B:67, ASN B:468, GLY B:175, GLY B:178, LYS B:51, CYS B:177, SER B:474, and THR B:176; conventional hydrogen bonds with the amino acid residue VAL B:475; π–π stacking interactions with the amino acid residue PHE B:439; π–π T-shaped interactions with the amino acid residue PHE B:392; amide-π stacking interactions with the amino acid residue GLY B:65; and alkyl interactions with the amino acid residues LEU B:334 and ALA B:438. Compound 7f formed van der Waals forces with the amino acid residues ILE B:100, ILE B:169, ILE B:204, LEU B:116, LEU B:356, MSE B:130, PRO B:113, VAL B:164, and PHE B:190; conventional hydrogen bonds with the amino acid residue ARG B:98; π–π T-shaped interactions with the amino acid residue PHE B:160; amide-π stacking interactions with the amino acid residues PHE B:172 and PRO B:171; and alkyl interactions with the amino acid residues VAL B203, LEU B:193, and ALA B:207 (ESI Material Fig. S65 and S66†).

**Fig. 3:**
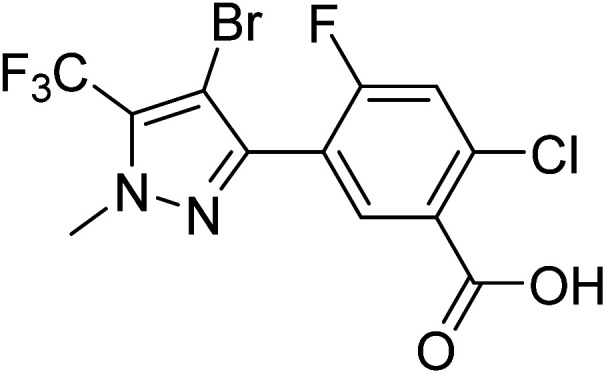
The chemical structure of the inhibitor.

Furthermore, Koch *et al.* reported the binding configuration of inhibitors (Fig. 3) with mitochondrial PPO from *N*. *tabacum*, inferring that the amino acid residues ARG 98 and PHE 392 play a very important role.^32^

Finally, the binding energies of compound 7f with PPO are both negative and spontaneous, with numerous interaction active sites. We speculated that compound 7f is a PPO enzyme inhibitor herbicide and that different interactions at different active sites lead to different herbicidal activities than those of fomesafen.

## 3 Materials and characterization

### 3.1 Instrumentation

All reagents (ethyl 4,4,4-trifluoroacetate, methylhydrazine, phosphorus oxychloride, 4-hydroxyphenylboronic acid, and methoxyamine hydrochloride, *etc*) used in the synthesis were purchased from Bide Pharmatech Ltd., while solvents (*N*,*N*-dimethylformamide (DMF), methanol, ethyl alcohol, ethyl acetate, and petroleum ether, *etc*) for experimental reactions and column chromatography were obtained from Hangzhou Hehui Chemical Co., Ltd. Notably, all materials were utilized directly without further purification. For biological experiments, fomesafen was provided as a positive control by the Biological Testing Center of Nankai University in China. The melting point of compounds was measured using a B-545 melting point meter, which does not require calibration. The NMR spectra were recorded with a Bruker AV-400 spectrometer, employing Chloroform-d or Dimethyl sulfoxide-d6 as solvents. The HRMS was performed using an Agilent 6545 Q-TOF liquid chromatography–mass spectrometer. Additionally, the seeds of six weeds (*E*. *crusgalli*, *D*. *sanguinalis*, *S*. *viridis*, *A*. *theophrasti*, *A*. *revolutus*, and *E*. *prosper*) were collected from fields near Nankai University in November 15, 2021 at Tianjin, and authenticated by Prof. Qiang Bian (Nankai University, Email: bianqiang@nankai.edu.cn). The voucher samples of these seeds had been deposited in National Engineering Research Center of Pesticide, Nankai University (EC-20211115-001, DS-20211115-001, SV-20211115-001, AT-20211115-001, AR-20211115-001, EP-20211115-001). No permission was required to access these samples.

### 3.2 Synthetic process

Scheme 2 shows the synthetic process of the compounds. Compound 4 was synthesized by cyclization chlorination, the Vilsmeier–Haack reaction, and the Suzuki cross-coupling reaction; compound 6 was prepared by etherification and oximation and 7a–7l were synthesized using an esterification reaction.

#### 3.2.1 Synthesis of 2

Ethyl 4,4,4-trifluoro-3-oxobutanoate (46.03 g, 0.25 mol) was mixed with a stirred solution containing acetic acid (15.01 g, 0.025 mol). The abovementioned solution was subsequently cooled to 10 °C, and 35% methyl hydrazine aqueous solution (32.91 g, 0.25 mol) was added to the above system. The resulting solution was stirred at 25 °C for 1 h and 80 °C for 5 h. The resulting solution was vacuum concentrated to obtain compound 2.^48,49^

##### 3.2.1.1 1-Methyl-3-(trifluoromethyl)-1*H*-pyrazol-5-ol (2)

White solid, yield 75.7%. ^1^H NMR (400 MHz, DMSO-*d*6) *δ*: 11.68 (s, 1H, OH), 5.72 (s, 1H, Pyrazole-H), 3.59 (s, 3H, CH_3_); ^13^C NMR (101 MHz, DMSO-*d*6) *δ*: 153.5, 138.7 (q, *J* = 111.5 Hz), 122.0 (d, *J* = 269.1 Hz), 84.8, 34.2; ^19^F NMR (377 MHz, DMSO-*d*6) *δ*: −56.65. HRMS calcd for C_5_H_5_F_3_N_2_NaO [M + Na]^+^ 189.0246, found 189.0243.

#### 3.2.2 Synthesis of 3

Phosphorus oxychloride (23.00 g, 0.15 mol) was slowly mixed with a continuously stirred DMF (10 mL) solution. After stirring at 0 °C for approximately 0.5 h, compound 2 (9.96 g, 0.06 mol) combined with DMF (60 mL) was subsequently added to the above reaction mixture while mixing at 110 °C for 6 h. Afterwards, the resulting reaction mixture was added to stirred ice water. 100 mL of ethyl acetate was used for the extraction of the mixture three times. Salt water and MgSO_4_ were used to wash and dry the mixed organic layers, respectively. The solution was vacuum concentrated, silica gel column chromatography was conducted by employing petroleum ether/ethyl acetate (3 : 1, v/v), and compound 3 was finally generated.^50,51^

##### 3.2.2.1 5-Chloro-1-methyl-3-(trifluoromethyl)-1*H*-pyrazole-4-carbaldehyde (3)

White solid, yield 72.4%.; ^1^H NMR (400 MHz, chloroform-*d*) *δ*: 9.93 (s, 1H, CHO), 3.95 (s, 3H, CH_3_); ^13^C NMR (101 MHz, chloroform-*d*) *δ*: 181.0, 141.8 (q, *J* = 270.7 Hz), 133.7, 120.0 (d, *J* = 271.4 Hz), 115.9, 36.9; ^19^F NMR (377 MHz, chloroform-*d*) *δ*: −61.62. HRMS calcd for C_6_H_4_ClF_3_N_2_NaO [M + Na]^+^ 234.9856, found 234.9859.

#### 3.2.3 Synthesis of 4

Palladium acetate (0.11 g, 0.47 mmol) was added to a mixture of compound 3 (2.00 g, 9.41 mmol), 4-hydroxybenzene boronic acid (1.43 g, 10.35 mmol), potassium carbonate (2. 06 g, 18.82 mmol), and triphenylphosphine (0.25 g, 0.94 mmol) dissolve in a complex solution system of 10 mL of acetonitrile combined with 20 mL of methanol. The mixed complex was stirred at 60 °C for 7 h, followed by the addition of water. Ethyl acetate (50 mL × 3) was used to extract the mixture three times. Salt water and MgSO_4_ were used to wash and dry the mixed organic layers, respectively. The solution was vacuum concentrated, and recrystallization was carried out in methanol and water to form compound 4.^11^

##### 3.2.3.1 5-(4-Hydroxyphenyl)-1-methyl-3-(trifluoromethyl)-1*H*-pyrazole-4-carbaldehyde (4)

White solid, yield 84.6%.; ^1^H NMR (400 MHz, chloroform-*d*) *δ*: 9.67 (s, 1H, CHO), 7.21 (d, *J* = 8.2 Hz, 2H, Ar–H), 6.92 (d, *J* = 8.2 Hz, 2H, Ar–H), 6.73 (s, 1H, OH), 3.76 (s, 3H, CH_3_); ^13^C NMR (101 MHz, chloroform-*d*) *δ*: 183.8, 158.2, 150.0, 141.6 (d, *J* = 39.3 Hz), 131.6, 121.9, 119.2, 117.7, 116.2, 37.6; ^19^F NMR (377 MHz, chloroform-*d*) *δ*: −61.69. HRMS calcd for C_12_H_9_F_3_N_2_NaO_2_ [M + Na]^+^ 293.0508, found 293.0513.

#### 3.2.4 Synthesis of 5

Compound 4 (2.00 g, 7.40 mmol) was dispersed in 80 mL of DMF, and potassium carbonate (2.05 g, 14.80 mmol) was then added to the mixture. The solution was stirred for approximately 0.5 h, and intermediate A (1.87 g, 7.77 mmol) was combined with the solution and stirred at 100 °C for 6 h. Water was subsequently added to the above reaction system, after which 30 mL of ethyl acetate was used to extract the mixture three times. Salt water and MgSO_4_ were used to wash and dry the mixed organic layers, respectively. The solution was vacuum concentrated, silica gel column chromatography was conducted by employing petroleum ether/ethyl acetate (3 : 1, v/v), and compound 5 was finally formed.^11^

##### 3.2.4.1 2-[2-(4-(4-(Formyl)methyl)-1-methyl-3-trifluoromethyl-1*H*-pyrazol-5-ylphenoxymethyl)phenyl]-3-methoxyacrylic acid methyl ester (5)

White solid, yield 90.1%.; ^1^H NMR (400 MHz, chloroform-*d*) *δ*: 9.73 (s, 1H, CHO), 7.63 (s, 1H, C=CH), 7.54 (d, *J* = 5.7 Hz, 1H, Ar–H), 7.38–7.35 (m, 2H, Ar–H), 7.29 (s, 1H, Ar–H), 7.26 (s, 1H, Ar–H), 7.22–7.19 (m, 1H, Ar–H), 7.06 (d, *J* = 8.6 Hz, 2H, Ar–H), 5.04 (s, 2H, ArCH_2_), 3.85 (s, 3H, CO_2_CH_3_), 3.81 (s, 3H, OCH_3_), 3.72 (s, 3H, CH_3_); ^13^C NMR (101 MHz, chloroform-d) *δ*: 183.0, 167.9, 160.6, 160.4, 149.7, 141.3 (d, *J* = 39.4 Hz), 135.3, 131.6, 131.4, 131.2, 128.3, 127.9, 127.5, 118.2, 117.8, 116.2, 115.4, 109.9, 68.2, 62.2, 51.8, 37.6; ^19^F NMR (377 MHz, chloroform-*d*) *δ*: −61.77. HRMS calcd for C_24_H_21_F_3_N_2_NaO_5_ [M + Na]^+^ 497.1295, found 497.1296.

#### 3.2.5 Synthesis of 6

Hydroxylamine hydrochloride (0.44 g, 6.33 mmol) and sodium acetate (0.55 g, 6.75 mmol) were mixed with a continuously stirred compound 5 (2.00 g, 4.22 mmol) solution. The resulting solution was subsequently refluxed for 6 h with 80% ethanol as the solvent. After the reaction, water was added to the resulting solution, after which 30 mL of ethyl acetate was used to extract the mixture three times. Salt water and MgSO_4_ were used to wash and dry the mixed organic layers, respectively. A vacuum concentration of the solution was used to acquire compound 6 for the next synthesis process.^52^

##### 3.2.5.1 2-[2-(4-(4-(Hydroxyimino)methyl)-1-methyl-3-trifluoromethyl-1*H*-pyrazol-5-ylphenoxymethyl)phenyl]-3-methoxyacrylic acid methyl ester (6)

White solid, yield 92.8%.; ^1^H NMR (400 MHz, chloroform-*d*) *δ*: 9.01 (s, 1H, CH = N), 7.89 (s, 1H, N–OH), 7.64 (s, 1H, C=CH), 7.56–7.53 (m, 1H, Ar–H), 7.36–7.34 (m, 2H, Ar–H), 7.26 (s, 1H, Ar–H), 7.22 (s, 1H, Ar–H), 7.20 (s, 1H, Ar–H), 7.02 (d, *J* = 8.4 Hz, 2H, Ar–H), 5.03 (s, 2H, ArCH_2_), 3.84 (s, 3H, CO_2_CH_3_), 3.76 (s, 3H, OCH_3_), 3.72 (s, 3H, CH_3_); ^13^C NMR (101 MHz, chloroform-*d*) *δ*: 168.1, 160.5, 160.1, 145.2, 141.0, 138.3 (q, *J* = 270.7 Hz), 135.4, 131.3, 131.2, 131.2, 128.3, 127.9, 127.5, 121.2 (d, *J* = 270.2 Hz), 119.5, 115.4, 110.9, 109.9, 68.2, 62.2, 51.9, 37.7; ^19^F NMR (377 MHz, chloroform-*d*) *δ*: −61.96. HRMS calcd for C_24_H_22_F_3_N_3_NaO_5_ [M + Na]^+^ 512.1404, found 512.1408.

#### 3.2.6 Synthesis of 7a–7l

A solution of 6 (0.6 g, 1.23 mmol) and TEA (0.24 g, 2.46 mmol) in DCM (15 mL) was stirred, and 1.35 mmol of acyl chloride was subsequently added to the solution and stirred at 25 °C for 7.5 h. When the above reaction was completed, the solution was mixed with 20 mL of water, and DCM (30 mL × 3) was used to extract the mixture three times. Salt water and MgSO_4_ were used to wash and dry the mixed organic layers, respectively. The solution was vacuum concentrated, silica gel column chromatography was conducted with petroleum ether/ethyl acetate (7 : 1, v/v), and the resulting products 7a–7l were obtained.^52^

##### 3.2.6.1 2-[2-(4-(4-(Acetoxyimino)methyl)-1-methyl-3-trifluoromethyl-1*H*-pyrazol-5-ylphenoxymethyl)phenyl]-3-methoxyacrylic acid methyl ester (7a)

White solid, yield 77.5%. m.p. 172.2–173.1 °C; ^1^H NMR (400 MHz, chloroform-*d*) *δ*: 8.12 (s, 1H, CH = N), 7.63 (s, 1H, C=CH), 7.56–7.52 (m, 1H, Ar–H), 7.38–7.34 (m, 2H, Ar–H), 7.27 (s, 1H, Ar–H), 7.24 (s, 1H, Ar–H), 7.22–7.19 (m, 1H, Ar–H), 7.03 (d, *J* = 8.7 Hz, 2H, Ar–H), 5.03 (s, 2H, ArCH_2_), 3.85 (s, 3H, CO_2_CH_3_), 3.80 (s, 3H, OCH_3_), 3.72 (s, 3H, CH_3_), 2.13 (s, 3H, CH_3_CO); ^13^C NMR (101 MHz, chloroform-*d*) *δ*: 170.4, 167.8, 160.3, 146.4, 146.4, 139.6 (d, *J* = 38.4 Hz, 135.4, 131.4, 131.3, 131.2, 130.6, 128.2, 127.9, 127.4, 120.9 (q, *J* = 270.7 Hz), 118.9, 115.3, 109.9, 109.1, 68.2, 62.1, 51.8, 37.9, 19.8; ^19^F NMR (377 MHz, chloroform-*d*) *δ*: −61.82. HRMS calcd for C_26_H_24_F_3_N_3_NaO_6_ [M + Na]^+^ 554.1509, found 554.1516.

##### 3.2.6.2 2-[2-(4-(4-(2-Chloroacetoxyimino)methyl)-1-methyl-3-trifluoromethyl-1*H*-pyrazol-5-ylphenoxymethyl)phenyl]-3-methoxyacrylic acid methyl ester (7b)

White solid, yield 81.5%. m.p. 135.2–136.4 °C; ^1^H NMR (400 MHz, chloroform-*d*) *δ*: 8.16 (s, 1H, CH = N), 7.92–7.61 (m, 2H, Ar–H and C=CH), 7.56–7.53 (m, 1H, Ar–H), 7.36 (d, *J* = 3.4 Hz, 2H, Ar–H), 7.22–7.20 (m, 2H, Ar–H), 7.07–7.01 (m, 2H, Ar–H), 5.04 (s, 2H, ArCH_2_), 4.20 (s, 2H, CH_2_Cl), 3.85 (s, 3H, CO_2_CH_3_), 3.80 (s, 3H, OCH_3_), 3.72 (s, 3H, CH_3_); ^13^C NMR (101 MHz, chloroform-*d*) *δ*: 167.9, 166.6, 160.4, 147.6, 146.7, 145.8 (d, *J* = 182.8 Hz), 135.3, 131.4, 131.2, 130.5, 127.9, 120.9 (q, *J* = 256.5 Hz), 118.7, 115.8, 115.4, 115.3, 109.9, 108.4, 68.3, 62.2, 51.8, 40.7, 37.9; ^19^F NMR (377 MHz, chloroform-*d*) *δ*: −61.83. HRMS calcd for C_26_H_23_ClF_3_N_3_NaO_6_ [M + Na]^+^ 588.1120, found 588.1113.

##### 3.2.6.3 2-[2-(4-(4-(Acryloyloxyimino)methyl)-1-methyl-3-trifluoromethyl-1*H*-pyrazol-5-ylphenoxymethyl)phenyl]-3-methoxyacrylic acid methyl ester (7c)

White solid, yield 77.5%. m.p. 142.2–144.1 °C; ^1^H NMR (400 MHz, chloroform-*d*) *δ*: 8.23 (s, 1H, CH = N), 7.65 (s, 1H, C=CH), 7.58 (d, *J* = 6.3 Hz, 1H, Ar–H), 7.40–7.36 (m, 2H, Ar–H), 7.32 (d, *J* = 8.4 Hz, 2H, Ar–H), 7.24–7.22 (m, 1H, Ar–H), 7.07 (d, *J* = 8.4 Hz, 2H, Ar–H and CH_2_ = CH̲), 6.55–6.51 (m, 1H, Ar–H), 6.29–6.22 (m, 1H, CH̲_2_ = CH), 5.91 (dd, *J* = 10.5, 1.4 Hz, 1H, CH̲_2_ = CH), 5.06 (s, 2H, ArCH_2_), 3.87 (s, 3H, CO_2_CH_3_), 3.83 (s, 3H, OCH_3_), 3.74 (s, 3H, CH_3_); ^13^C NMR (101 MHz, chloro-form-*d*) *δ*: 167.9, 164.0, 160.3, 147.6, 146.3, 140.1 (d, *J* = 86.9 Hz), 135.4, 132.4, 131.6, 131.3, 131.2, 128.2, 127.8, 127.5, 125.9, 121.0 (q, *J* = 258.6 Hz), 118.9, 115.3, 109.9, 109.0, 68.2, 62.1, 51.8, 38.0; ^19^F NMR (377 MHz, chloroform-*d*) *δ*: −61.84. HRMS calcd for C_27_H_24_F_3_N_3_NaO_6_ [M + Na]^+^ 566.1509, found 566.1518.

##### 3.2.6.4 2-[2-(4-(4-(Methacryloyloxyimino)methyl)-1-methyl-3-trifluoromethyl-1*H*-pyrazol-5-ylphenoxymethyl)phenyl]-3-methoxyacrylic acid methyl ester (7d)

White solid, yield 83.4%. m.p. 111.3–113.9 °C; ^1^H NMR (400 MHz, chloroform-*d*) *δ*: 7.92–8.22 (m, 1H, CH = N), 7.65–7.64 (m, 1H, C=CH), 7.59–7.55 (m, 1H, Ar–H), 7.40–7.31 (m, 3H, Ar–H), 7.28 (s, 1H, Ar–H), 7.26–7.21 (m, 2H, Ar–H), 7.06 (dt, *J* = 13.3, 5.4 Hz, 2H, CH̲_2_ = CCH_3_), 6.16 (s, 1H, Ar–H), 5.05 (s, 2H, ArCH_2_), 3.87 (s, 3H, CO_2_CH_3_), 3.83 (s, 3H, OCH_3_), 3.79 (s, 3H,CH_2_ = CH̲_3_), 3.74 (s, 3H, CH_3_); ^13^C NMR (101 MHz, chloroform-*d*) δ: 169.3, 167.9, 167.9, 160.3, 160.1, 148.8, 147.0 (d, *J* = 99.0 Hz), 145.0, 135.5, 131.6, 131.6, 131.3, 131.2, 128.2, 127.9, 127.5, 120.6 (q, *J* = 258.7 Hz), 115.3 (2C), 109.9, 104.1, 68.2, 62.1, 51.8, 38.0, 37.7; ^19^F NMR (377 MHz, chloroform-*d*) *δ*: −61.81. HRMS calcd for C_28_H_26_F_3_N_3_NaO_6_ [M + Na]^+^ 580.1666, found 580.1672.

##### 3.2.6.5 2-[2-(4-(4-(Benzoyloxyimino)methyl)-1-methyl-3-trifluoromethyl-1*H*-pyrazol-5-ylphenoxymethyl)phenyl]-3-methoxyacrylic acid methyl ester methyl (7e)

White solid, yield 83.6%. m.p. 132.5–134.2 °C; ^1^H NMR (400 MHz, chloroform-*d*) *δ*: 8.35 (s, 1H, CH = N), 8.07–8.02 (m, 2H, Ar–H), 7.63 (s, 1H, C=CH), 7.57 (d, *J* = 6.2 Hz, 2H, Ar–H), 7.45 (t, *J* = 7.8 Hz, 2H, Ar–H), 7.35 (m, 4H, Ar–H), 7.23–7.19 (m, 1H, Ar–H), 7.07 (d, *J* = 8.7 Hz, 2H, Ar–H), 5.05 (s, 2H, ArCH_2_), 3.85 (s, 3H, CO_2_CH_3_), 3.82 (s, 3H, OCH_3_), 3.72 (s, 3H, CH_3_); ^13^C NMR (101 MHz, chloroform-*d*) *δ*: 167.9, 163.5, 160.3, 148.2, 146.3, 140.2, 139.8, 135.5, 133.3, 131.7, 131.3, 131.2, 130.5, 129.7, 128.6, 128.5, 128.2, 127.8, 127.5, 118.9, 118.1 (q, *J* = 315.1 Hz), 109.9, 109.0, 68.2, 622, 51.8, 38.0; ^19^F NMR (377 MHz, chloroform-*d*) *δ*: −61.52. HRMS calcd for C_31_H_26_F_3_N_3_NaO_6_ [M + Na]^+^ 616.1666, found 616.1674.

##### 3.2.6.6 2-[2-(4-(4-(4-Methylbenzoyloxyimino)methyl)-1-methyl-3-trifluoromethyl-1*H*-pyrazol-5-ylphenoxymethyl)phenyl]-3-methoxyacrylic acid methyl ester methyl (7f)

White solid, yield 71.2%. m.p. 153.2–154.3 °C; ^1^H NMR (400 MHz, chloroform-*d*) *δ*: 8.37 (s, 1H, CH = N), 7.96 (d, *J* = 8.1 Hz, 2H, Ar–H), 7.65 (s, 1H, C=CH), 7.61–7.58 (m, 1H, Ar–H), 7.38 (m, 4H, Ar–H), 7.28–7.23 (m, 3H, Ar–H), 7.10 (d, *J* = 8.4 Hz, 2H, Ar–H), 5.07 (s, 2H, ArCH_2_), 3.87 (s, 3H, CO_2_CH_3_), 3.84 (s, 3H, OCH_3_), 3.74 (s, 3H, CH_3_), 2.43 (s, 3H, Ar-CH_3_). ^13^C NMR (101 MHz, chloro-form-*d*) *δ*: 167.9, 163.6, 160.4, 160.3, 148.0, 146.2, 1441, 140.0 (d, *J* = 37.4 Hz), 135.5, 1317, 131.3, 131.2, 129.7, 129.3, 128.2, 127.8, 127.5, 125.8, 121.0 (q, *J* = 270.7 Hz), 119.0, 115.2, 109.9, 109.1, 68.2, 62.2, 51.8, 38.0, 21.7; ^19^F NMR (377 MHz, chloroform-*d*) *δ*: −61.49. HRMS calcd for C_32_H_28_F_3_N_3_NaO_6_ [M + Na]^+^ 630.1822, found 630.1826.

##### 3.2.6.7 2-[2-(4-(4-(2-Methylbenzoyloxyimino)methyl)-1-methyl-3-trifluoromethyl-1*H*-pyrazol-5-ylphenoxymethyl)phenyl]-3-methoxyacrylic acid methyl ester methyl (7g)

White solid, yield 88.7%. m.p. 166.9–168.6 °C; ^1^H NMR (400 MHz, chloroform-*d*) *δ*: 8.33 (s, 1H, CH = N), 7.91–7.87 (m, 1H, Ar–H), 7.65 (s, 1H, C=CH), 7.61–7.57 (m, 1H, Ar–H), 7.39–7.39 (m, 4H, Ar–H), 7.29–7.21 (m, 4H, Ar–H), 7.09 (d, *J* = 8.4 Hz, 2H, Ar–H), 5.07 (s, 2H, ArCH_2_), 3.87 (s, 3H, CO_2_CH_3_), 3.84 (s, 3H, OCH_3_), 3.74 (s, 3H, CH_3_), 2.63 (s, 3H, Ar-CH_3_); ^13^C NMR (101 MHz, chloroform-*d*) *δ*: 167.9, 164.3, 160.3, 148.0, 146.3, 140.6, 139.9 (d, *J* = 38.4 Hz), 135.4, 132.3, 131.8, 131.6, 131.4, 131.3, 131.2, 130.3, 128.2, 128.0, 127.8, 127.5, 125.7, 121.0 (q, *J* = 270.7 Hz), 118.9, 115.3, 109.9, 109.1, 68.2, 62.2, 51.8, 38.0, 21.5; ^19^F NMR (377 MHz, chloroform-*d*) *δ*: −61.65. HRMS calcd for C_32_H_28_F_3_N_3_NaO_6_ [M + Na]^+^ 630.1822, found 630.1824.

##### 3.2.6.8 2-[2-(4-(4-(3-Methylbenzoyloxyimino)methyl)-1-methyl-3-trifluoromethyl-1*H*-pyrazol-5-ylphenoxymethyl)phenyl]-3-methoxyacrylic acid methyl ester methyl (7h)

White solid, yield 73.4%. m.p. 139.8–140.7 °C; ^1^H NMR (400 MHz, chloroform-*d*) *δ*: 8.38 (s, 1H, CH = N), 7.87 (d, *J* = 9.3 Hz, 2H, Ar–H), 7.66 (s, 1H, C=CH), 7.59 (d, *J* = 6.0 Hz, 1H, Ar–H), 7.40–7.36 (m, 4H, Ar–H), 7.23 (d, *J* = 6.6 Hz, 1H, Ar–H), 7.10 (d, *J* = 8.4 Hz, 2H, Ar–H), 5.07 (s, 2H, ArCH_2_), 3.87 (s, 3H, CO_2_CH_3_), 3.84 (s, 3H, OCH_3_), 3.74 (s, 3H, CH_3_), 2.42 (s, 3H, Ar-CH_3_); ^13^C NMR (101 MHz, chloroform-*d*) *δ*: 167.9, 1637, 160.3, 148.1, 146.2, 140.0 (d, *J* = 38.4 Hz), 138.4, 135.5, 134.1, 132.0, 131.7, 131.3, 131.2, 130.2, 128.5, 128.4 (2C), 128.2, 127.8, 127.5, 126.8, 121.0 (q, *J* = 270.7 Hz), 119.0, 115.3 (2C), 109.9, 109.1, 68.2, 62.2, 51.8, 38.0, 21.3; ^19^F NMR (377 MHz, chloroform-*d*) *δ*: −61.80. HRMS calcd for C_32_H_28_F_3_N_3_NaO_6_ [M + Na]^+^ 630.1822, found 630.1829.

##### 3.2.6.9 2-[2-(4-(4-(4-Methoxybenzoyloxyimino)methyl)-1-methyl-3-trifluoromethyl-1*H*-pyrazol-5-ylphenoxymethyl)phenyl]-3-methoxyacrylic acid methyl ester methyl (7i)

White solid, yield 81.8%. m.p. 144.8–146.9 °C; ^1^H NMR (400 MHz, chloroform-*d*) *δ*: 8.35 (s, 1H, CH = N), 8.02 (d, *J* = 8.4 Hz, 2H, Ar–H), 7.65 (s, 1H, C=CH), 7.59 (d, *J* = 7.7 Hz, 1H, Ar–H), 7.38 (m, 4H, Ar–H), 7.23 (d, *J* = 7.0 Hz, 1H, Ar–H), 7.12–7.06 (m, 2H, Ar–H), 6.94 (d, *J* = 8.5 Hz, 2H, Ar–H), 5.07 (s, 2H, ArCH_2_), 3.87 (m, 6H, CO_2_CH_3_ and Ar-OCH_3_), 3.84 (s, 3H, OCH_3_), 3.74 (s, 3H, CH_3_); ^13^C NMR (101 MHz, chloroform-*d*) *δ*: 167.9, 163.7, 1633, 160.4, 160.3, 147.9, 146.2, 139.9 (d, *J* = 38.4 Hz), 135.5, 131.8, 131.7, 131.3, 131.2, 128.2, 127.8, 127.5, 121.0 (q, *J* = 270.7 Hz), 120.8, 119.0, 115.2, 113.8, 109.9, 109.2, 68.2, 62.2, 55.5, 51.8, 38.00; ^19^F NMR (377 MHz, chloroform-*d*) *δ*: −62.12. HRMS (ESI): calculated for C_32_H_28_F_3_N_3_NaO_7_ [M + Na]^+^ 646.1772, found 646.1778.

##### 3.2.6.10 2-[2-(4-(4-(4-Chlorobenzoyloxyimino)methyl)-1-methyl-3-trifluoromethyl-1*H*-pyrazol-5-ylphenoxymethyl)phenyl]-3-methoxyacrylic acid methyl ester methyl (7j)

White solid, yield 71.3%. m.p. 166.2–167.1 °C; ^1^H NMR (400 MHz, chloroform-*d*) *δ*: 8.33 (s, 1H, CH = N), 7.98 (d, *J* = 8.6 Hz, 2H, Ar–H), 7.63 (s, 1H, C=CH), 7.58–7.55 (m, 1H, Ar–H), 7.42 (d, *J* = 8.6 Hz, 2H, Ar–H), 7.38–7.32 (m, 4H, Ar–H), 7.22–7.19 (m, 1H, Ar–H), 7.07 (d, *J* = 8.8 Hz, 2H, Ar–H), 5.05 (s, 2H, ArCH_2_), 3.85 (s, 3H, CO_2_CH_3_), 3.82 (s, 3H, OCH_3_), 3.72 (s, 3H, CH_3_); ^13^C NMR (101 MHz, chloroform-*d*) *δ*: 167.9, 162.7, 160.4, 160.3, 148.4, 146.4, 139.8, 135.4, 131.6, 131.3, 131.2, 131.1, 128.9, 128.2, 127.8, 127.5, 127.1, 121.0 (q, *J* = 270.7 Hz), 118.9, 115.8, 115.3, 109.9, 108.9, 68.2, 62.2, 51.8, 38.0; ^19^F NMR (377 MHz, chloroform-*d*) *δ*: −61.58. HRMS calcd for C_31_H_25_ClF_3_N_3_NaO_6_ [M + Na]^+^ 650.1276, found 650.1276.

##### 3.2.6.11 2-[2-(4-(4-(4-Nitrobenzoyloxyimino)methyl)-1-methyl-3-trifluoromethyl-1*H*-pyrazol-5-ylphenoxymethyl)phenyl]-3-methoxyacrylic acid methyl ester methyl (7k)

White solid, yield 82.5%. m.p. 182.2–184.5 °C; ^1^H NMR (400 MHz, chloroform-*d*) *δ*: 8.39 (s, 1H, CH = N), 8.32 (d, *J* = 8.9 Hz, 1H, Ar–H), 8.24 (d, *J* = 8.9 Hz, 1H, Ar–H), 7.92 (s, 1H, Ar–H), 7.65 (s, 1H, C=CH), 7.58–7.56 (m, 1H, Ar–H), 7.39–7.36 (m, 2H, Ar–H), 7.23 (d, *J* = 8.7 Hz, 3H, Ar–H), 7.10 (d, *J* = 8.7 Hz, 1H, Ar–H), 7.04 (d, *J* = 8.7 Hz, 2H, Ar–H), 5.05 (s, 2H, ArCH_2_), 3.87 (s, 3H, CO_2_CH_3_), 3.79 (s, 3H, OCH_3_), 3.74 (s, 3H, CH_3_); ^13^C NMR (101 MHz, chloroform-*d*) *δ*: 168.0, 167.9, 160.3, 160.1, 150.7, 149.1, 146.6, 145.0, 141.2, 138.5 (d, *J* = 38.4 Hz), 135.4, 134.2, 131.6, 131.3, 131.2, 130.8, 128.3, 127.9, 127.5, 123.7, 120.6 (q, *J* = 384.8 Hz), 119.6, 115.4, 110.9, 109.9, 68.2, 62.1, 51.8, 38.0; ^19^F NMR (377 MHz, chloroform-*d*) *δ*: −61.98. HRMS calcd for C_31_H_25_F_3_N_4_NaO_8_ [M + Na]^+^ 661.1517, found 661.1522.

##### 3.2.6.12 2-[2-(4-(4-(4-Trifluoromethylbenzoyloxyimino)methyl)-1-methyl-3-trifluoromethyl-1*H*-pyrazol-5-ylphenoxymethyl)phenyl]-3-methoxyacrylic acid methyl ester methyl (7l)

White solid, yield 72.9%. m.p. 114.5–116.1 °C; ^1^H NMR (400 MHz, chloroform-*d*) *δ*: 8.37 (s, 1H, CH = N), 8.29 (s, 1H, Ar–H), 8.24 (d, *J* = 7.9 Hz, 1H, Ar–H), 7.83 (d, *J* = 7.9 Hz, 1H, Ar–H), 7.65 (s, 1H C=CH), 7.60–7.59 (m, 2H, Ar–H), 7.38–7.31 (m, 4H, Ar–H), 7.20 (d, *J* = 7.1 Hz, 1H, Ar–H), 7.10–7.05 (m, 2H, Ar–H), 5.04 (s, 2H, ArCH_2_), 3.85 (s, 3H, CO_2_CH_3_), 3.83 (s, 3H, OCH_3_), 3.72 (s, 3H, CH_3_); ^13^C NMR (101 MHz, chloroform-*d*) *δ*: 167.9, 162.3, 160.4, 160.3, 148.8, 146.4, 140.1 (d, *J* = 37.4 Hz), 135.4, 131.6, 131.3, 131.2, 131.0, 129.8 (q, *J* = 4.0 Hz), 129.3, 128.2, 127.8, 127.5, 126.5 (q, *J* = 4.0 Hz), 124.9, 122.3, 118.8, 115.3, 109.9, 108.8, 68.2, 62.1, 51.8, 38.0; ^19^F NMR (377 MHz, chloroform-*d*) *δ*: −62.18, −63.13. HRMS calcd for C_32_H_25_F_6_N_3_NaO_6_ [M + Na]^+^ 684.1540, found 684.1542.

### 3.3 Herbicidal activity test

The herbicidal activity of compounds 7a–7l was evaluated using pot culture, specifically through post-seedling stem and leaf spray treatments. Six target weeds (*E*. *crusgalli*, *D*. *sanguinalis*, *S*. *viridis*, *A*. *theophhrasti*, *A*. *retroflexus*, and *E*. *stratege*) were directly sown, achieving a germination rate of over 85%. A 0.2 cm layer of soil was then added on top, and the pots were cultivated in a greenhouse until the weeds developed approximately three leaves. The experimental product was dispersed in DMF with 0.1% Tween 80 as an emulsifier, and distilled water was added to reach the desired concentration. The control group was treated with only distilled water, while sulfonamide served as a positive control. After 20 days, the weed control activity was assessed, and the results are presented in Tables 1.^53–56^ The seeds of six plants were collected in November 15, 2021 at Tianjin, and authenticated by Prof. Qiang Bian (Nankai University, Email: bianqiang@nankai.edu.cn). The voucher samples of these seeds had been deposited in National Engineering Research Center of Pesticide, Nankai University (EC-20211115-001, DS-20211115-001, SV-20211115-001, AT-20211115-001, AR-20211115-001, EP-20211115-001). The weeds selected for herbicidal activity testing were obtained by cultivating germinated seeds.

## 4 Conclusions

In this paper, multiple innovative phenylpyrazole derivatives with strobilurin moieties were designed and prepared. Specifically, compound 7f exhibited an 89.0% fresh weight inhibition rate against *A. retroflexus*, which was comparable to that of fomesafen and has the potential to continue research and development as a candidate compound for herbicides. In addition, through molecular docking simulation, compound 7f can spontaneously bind with PPO from *N*. *tabacum* with high binding energy. There were numerous binding sites between the test compound and PPO. It is speculated that compound 7f is a PPO inhibitor. And its different active sites lead to differences in herbicidal activity compared to fomesafen. A comparison of the chemical structures revealed that fomesafen is an herbicide containing a diphenyl ether structure, but the title compounds are a series of phenylpyrazole derivatives with strobilurin moieties. According to molecular docking simulations, the strobilurin moieties interact with numerous amino acid residues. Different amino acid residues are caused by different functional groups. From the perspective of herbicide activity, this is the reason why the herbicidal spectra of the title compounds were different from those of fomesafen.

## Data availability

The data supporting this article have been included as part of the ESI.†

## Author contributions

Wenliang Zhang, Xiaodong Jin, and Wenwu Cheng conducted the experiments. Wenliang Zhang drafted the manuscript. Shulin Hao carried out the docking simulation and analyzed the data. Xiaohua Du supervised the project and revised the paper. All authors have read and agreed to the published version of the manuscript.

## Conflicts of interest

No potential conflict of interest was reported by the authors.

## Supplementary Material

RA-015-D5RA02377G-s001
