# Supplementary material for: Design, synthesis, herbicidal activity, and the molecular docking study of novel phenylpyrazole derivatives with strobilurin moieties
Source: RSC Adv. 2025 May 14;15(20):16088–96. doi: 10.1039/d5ra02377g (PMC12076197; doi:10.1039/d5ra02377g)
Supplement: RA-015-D5RA02377G-s001 [file RA-015-D5RA02377G-s001.pdf]

Supplementary Information (SI) for RSC Advances.
This journal is © The Royal Society of Chemistry 2025

Supplementary Material

Contents:

1.The ADME properties of compound a 2

2.^{1}H NMR, ^{13}C NMR and ^{19}F NMR spectra of target compounds 2-6 and 7a – 7l.........3

3. IR spectroscopy of target compounds 7a – 7l 54

4.Figure A and Figure B..66

1.The ADME properties of compound a

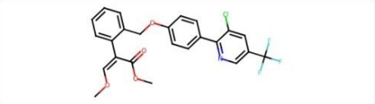

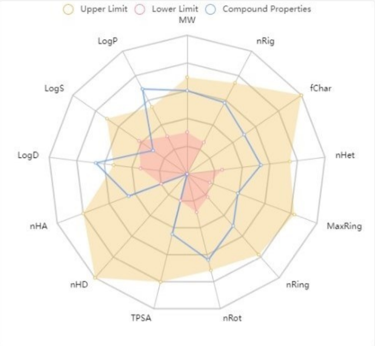

Physicochemical Property		
Molecular Weight (MW)	477.100	
Volume	448.561	
Density	1.064	
nHA	5	
nHD	0	
nRot	9	
nRing	3	
MaxRing	6	
nHet	9	
fChar	0	
nRig	20	
Flexibility	0.450	
Stereo Centers	0	
TPSA	57.650	
logS	-5.954	
logP	4.818	
logD	4.316	

Medicinal Chemistry		
QED	0.232	
SAscore	2.567	
Fsp^3	0.167	
MCE-18	21.000	
NPscore	-0.683	
Lipinski Rule	Accepted	
Pfizer Rule	Rejected	
GSK Rule	Rejected	
Golden Triangle	Accepted	
PAINS	0 alert(s)	
ALARM NMR Rule	2 alert(s)	DETAIL
BMS Rule	0 alert(s)	
Chelator Rule	0 alert(s)	
**Absorption**		
Caco-2 Permeability	-4.742	
MDCK Permeability	1.4e-05	
Pgp-inhibitor	+	
Pgp-substrate	---	
HIA	---	
$F_{20\%}$	---	
$F_{30\%}$	---	
**Distribution**		
PPB	99.443%	
VD	0.934	
BBB Penetration	---	
Fu	0.984%	
**Metabolism**		
CYP1A2 inhibitor	+	
CYP1A2 substrate	+++	
CYP2C19 inhibitor	++	
CYP2C19 substrate	--	
CYP2C9 inhibitor	++	
CYP2C9 substrate	-	
CYP2D6 inhibitor	---	
CYP2D6 substrate	--	
CYP3A4 inhibitor	-	
CYP3A4 substrate	++	
**Excretion**		
CL	9.008	
$T_{1/2}$	0.012	

Toxicity		
hERG Blockers	--	
H-HT	+++	
DILI	+++	
AMES Toxicity	---	
Rat Oral Acute Toxicity	---	
FDAMDD	++	
Skin Sensitization	---	
Carcinogencity	-	
Eye Corrosion	---	
Eye Irritation	---	
Respiratory Toxicity	--	
**Environmental Toxicity**		
Bioconcentration Factors	2.134	
IGC_{50}	5.033	
$LC_{50}FM$	6.536	
$LC_{50}DM$	6.776	
**Tox21 Pathway**		
NR-AR	--	
NR-AR-LBD	-	
NR-AhR	++	
NR-Aromatase	+++	
NR-ER	++	
NR-ER-LBD	+	
NR-PPAR-gamma	---	
SR-ARE	+++	
SR-ATAD5	++	
SR-HSE	---	
SR-MMP	+++	
SR-p53	+++	
**Toxicophore Rules**		
Acute Toxicity Rule	0 alert(s)	
Genotoxic Carcinogenicity Rule	1 alert(s)	DETAIL
NonGenotoxic Carcinogenicity Rule	0 alert(s)	
Skin Sensitization Rule	2 alert(s)	DETAIL
Aquatic Toxicity Rule	6 alert(s)	DETAIL
NonBiodegradable Rule	4 alert(s)	DETAIL
SureChEMBL Rule	0 alert(s)	
FAF-Drugs4 Rule	1 alert(s)	DETAIL

Tip: For the classification endpoints , the prediction probability values are transformed into six symbols: 0-0.1(---), 0.1-0.3(--), 0.3-0.5(-), 0.5-0.7(+), 0.7-0.9(++), and 0.9-1.0(+++).

Figure S1. The ADME properties of compound a

2.^{1}H NMR, ^{13}C N

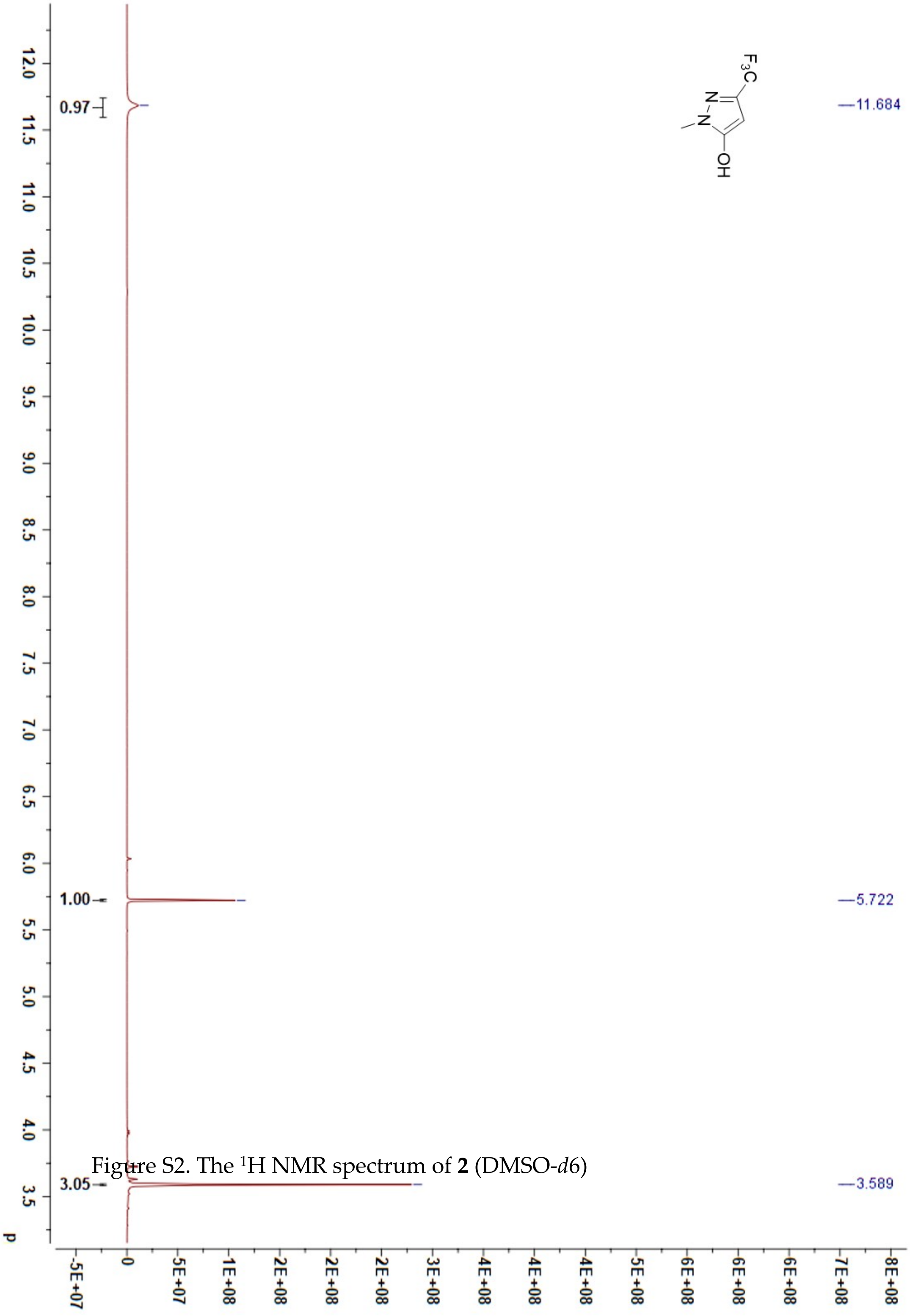

Figure S2. The ^{1}H NMR spectrum of **2** (DMSO-*d*6)

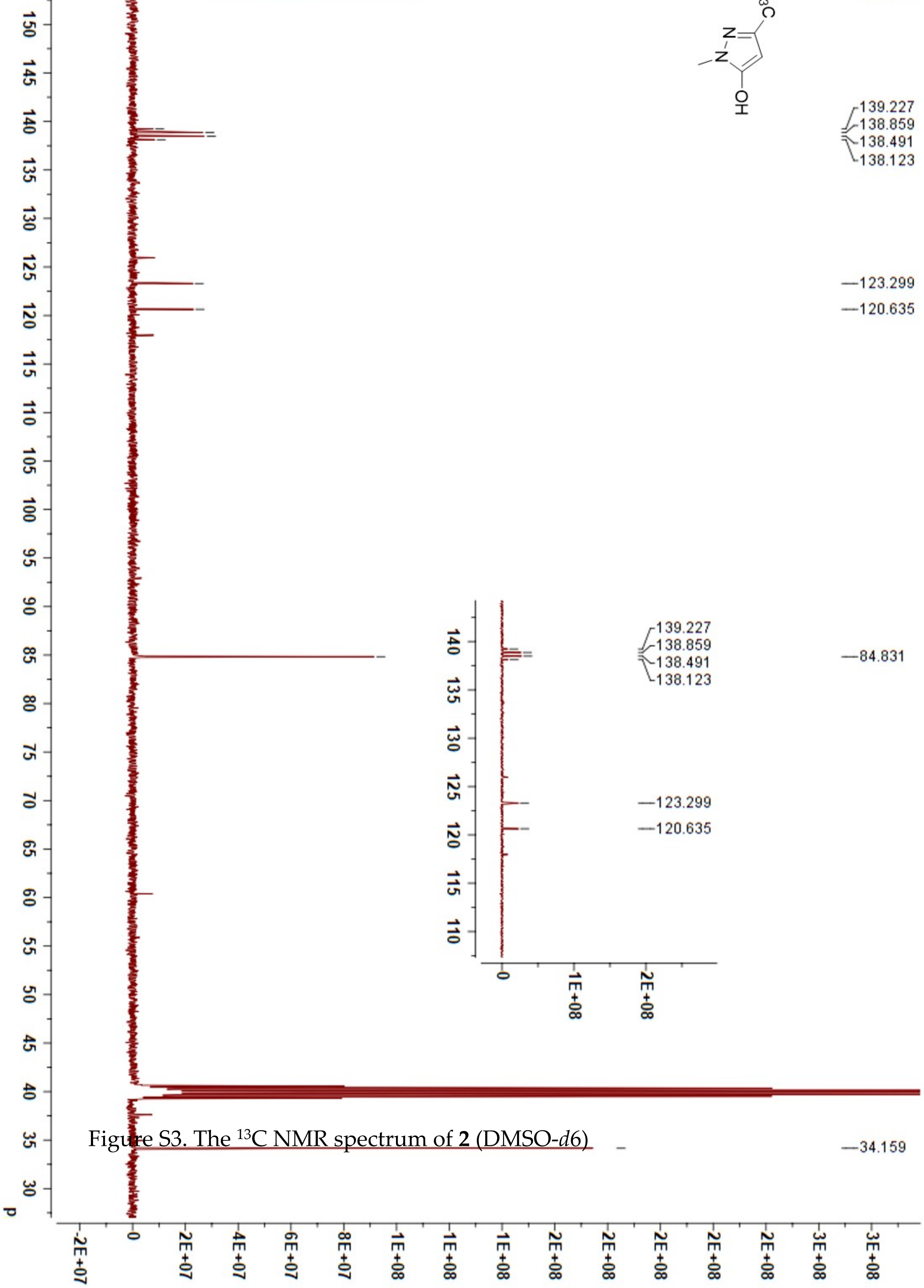

Figure S3. The ^{13}C NMR spectrum of **2** (DMSO-*d*6)

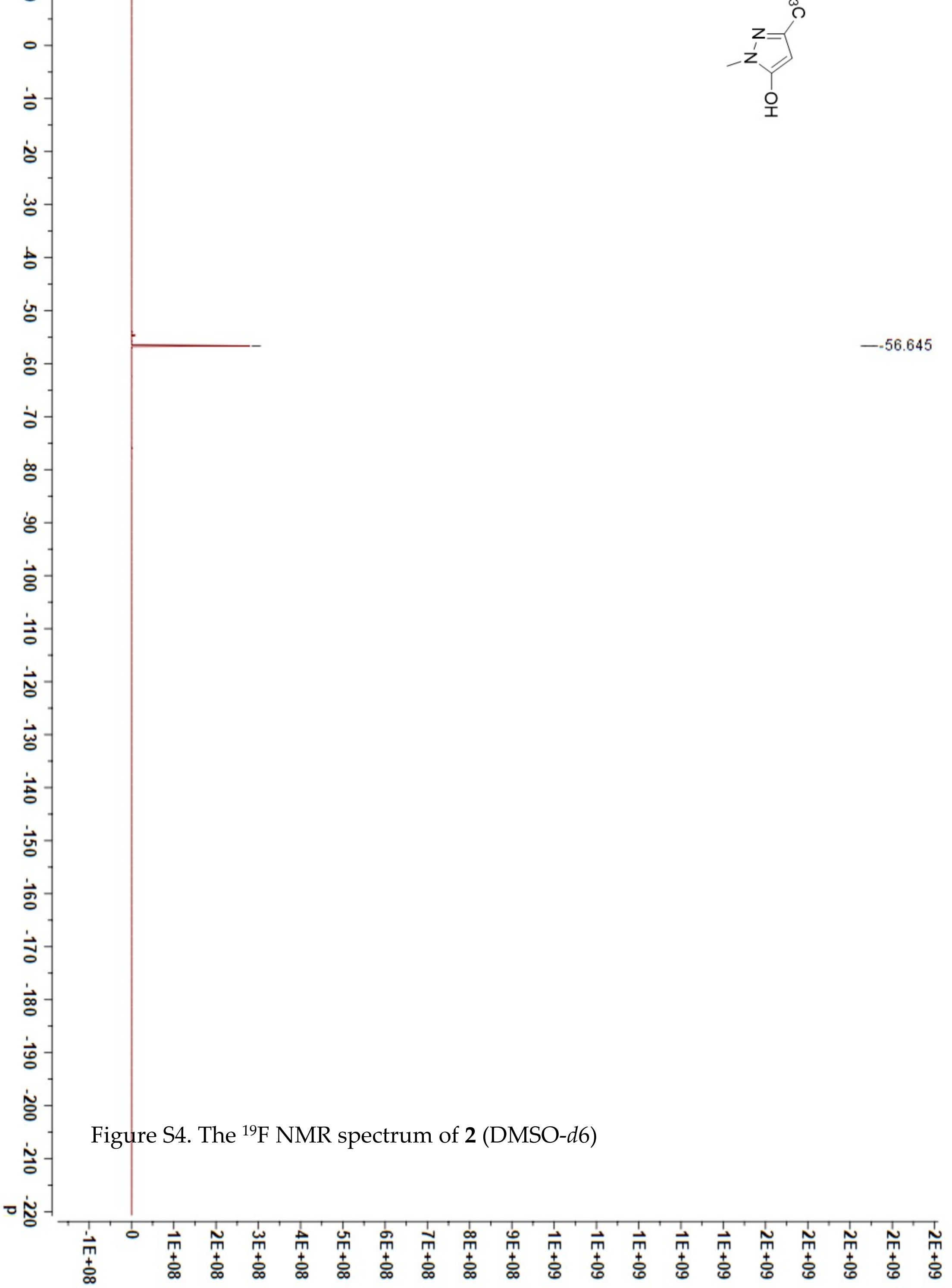

Figure S4. The ^{19}F NMR spectrum of **2** (DMSO-*d*6)

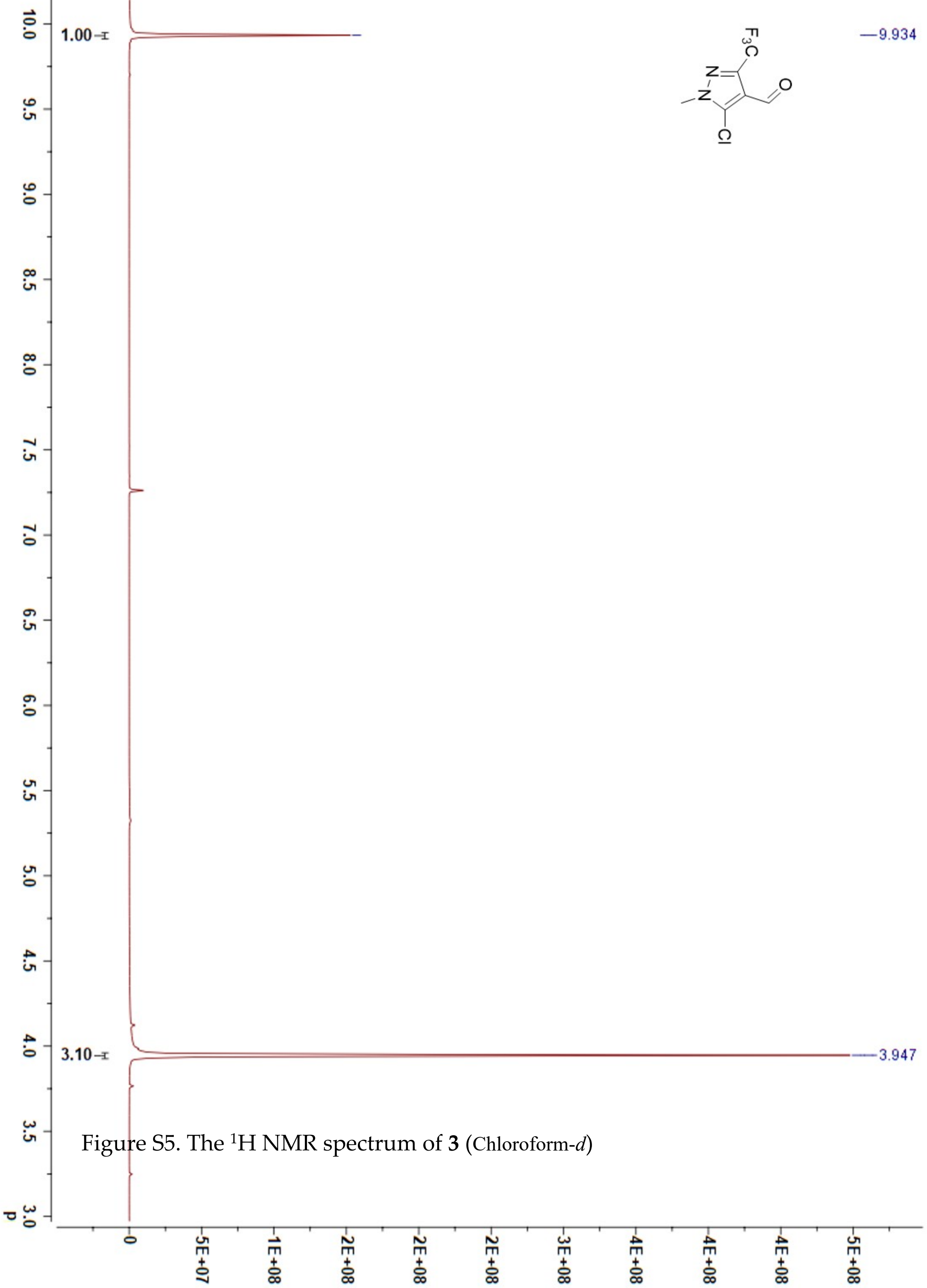

Figure S5. The ^{1}H NMR spectrum of **3** (Chloroform-*d*)

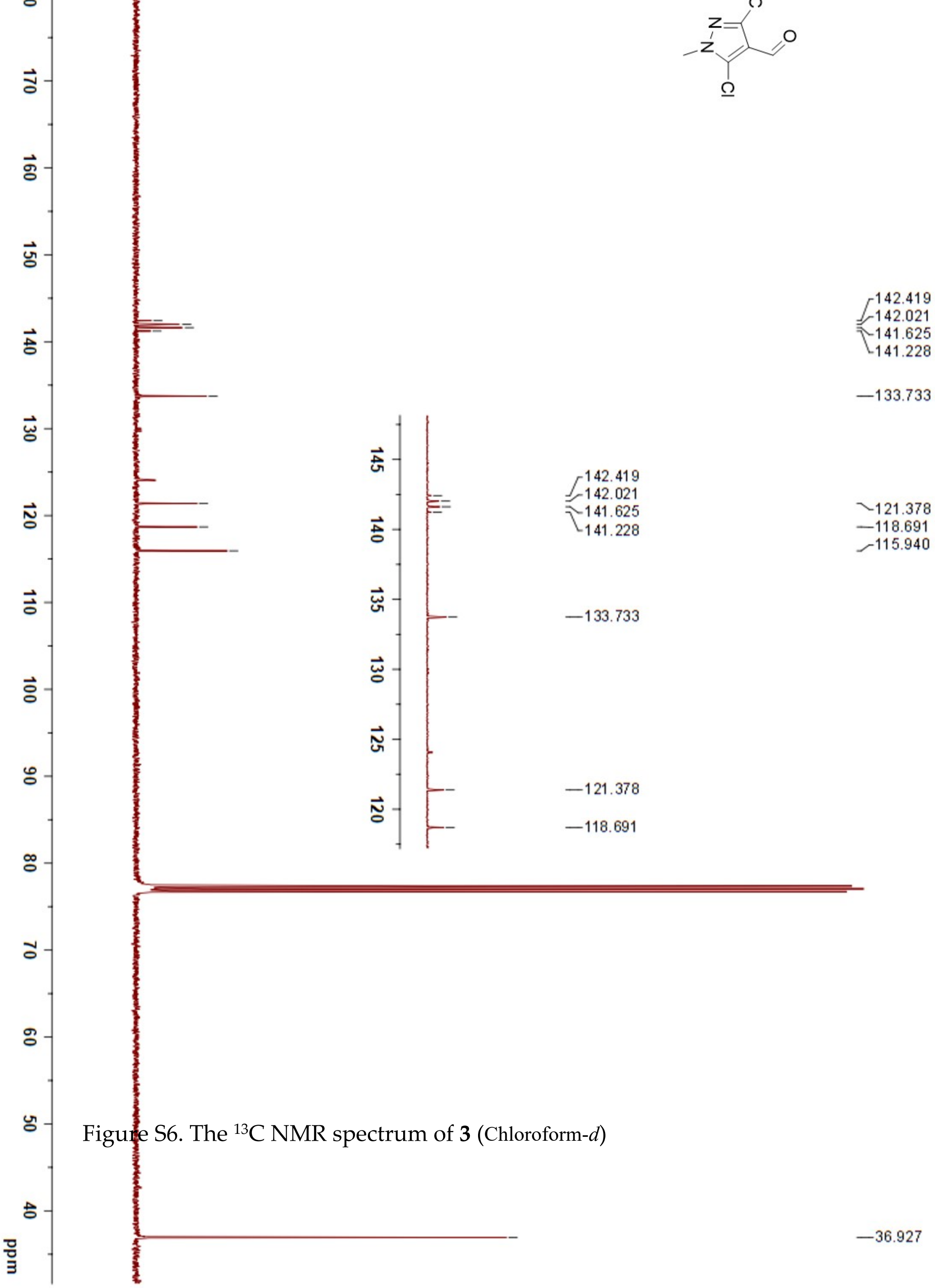

Figure S6. The ^{13}C NMR spectrum of **3** (Chloroform-*d*)

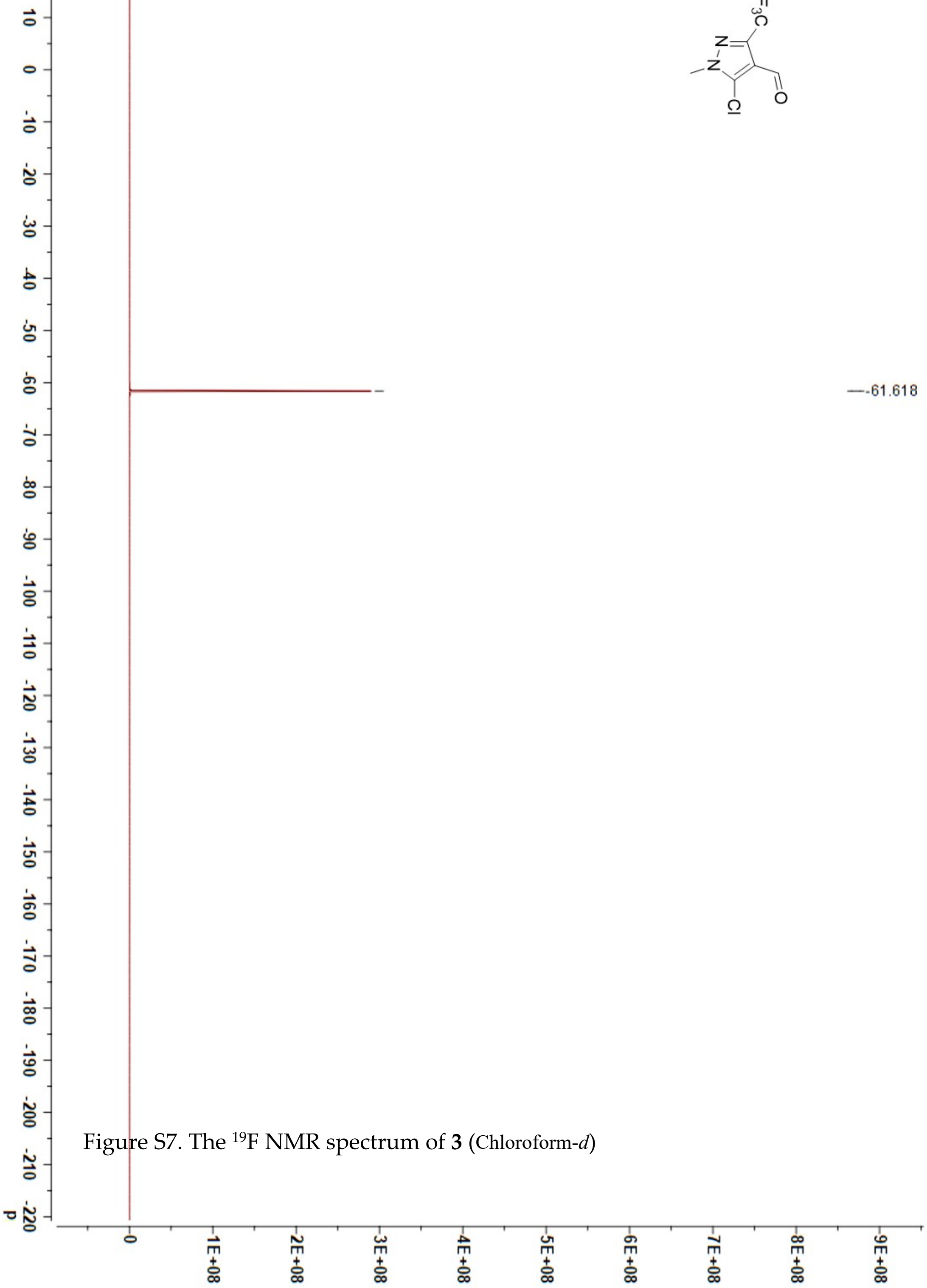

Figure S7. The ^{19}F NMR spectrum of **3** (Chloroform-*d*)

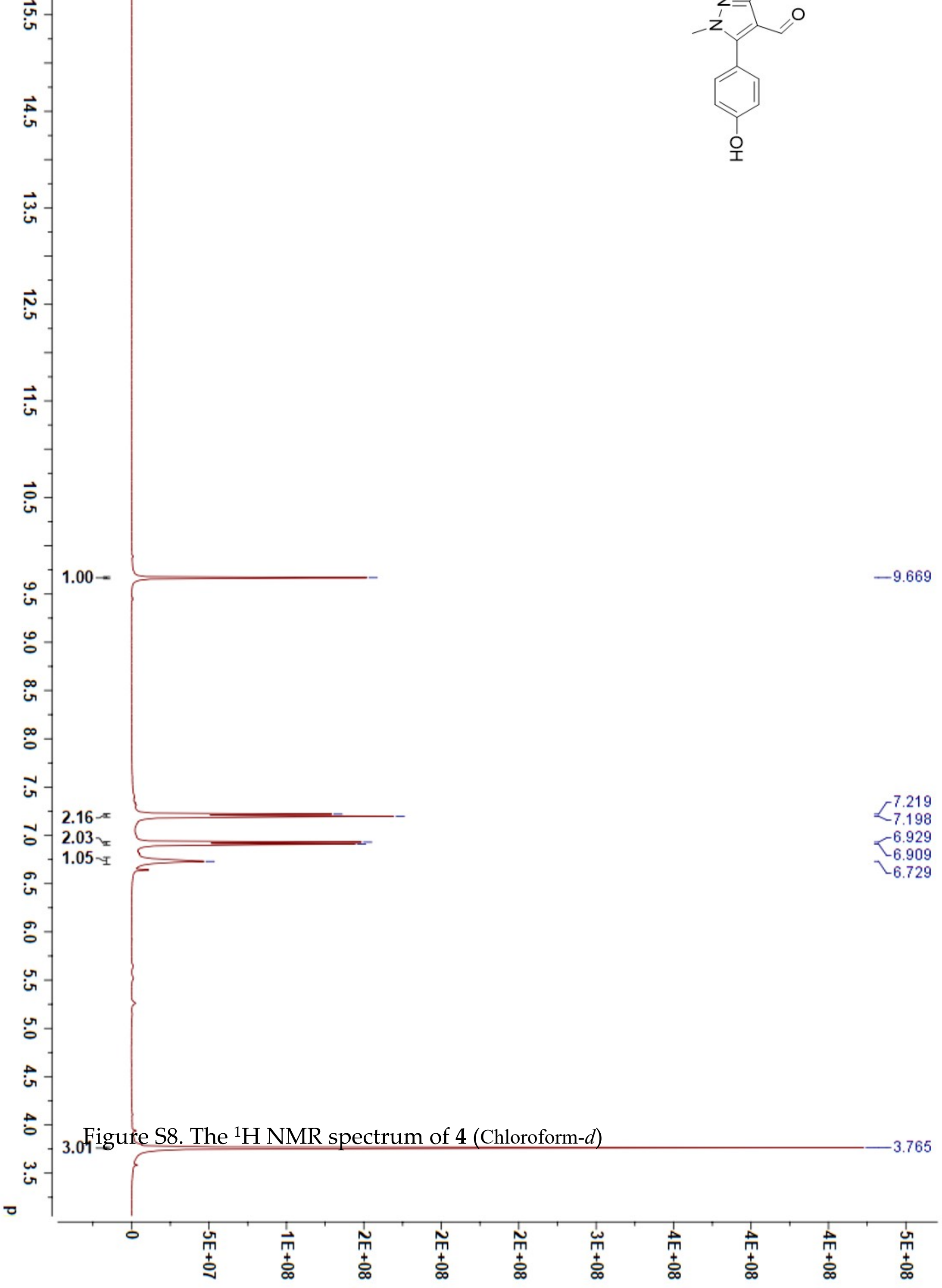

Figure S8. The ¹H NMR spectrum of **4** (Chloroform-*d*)

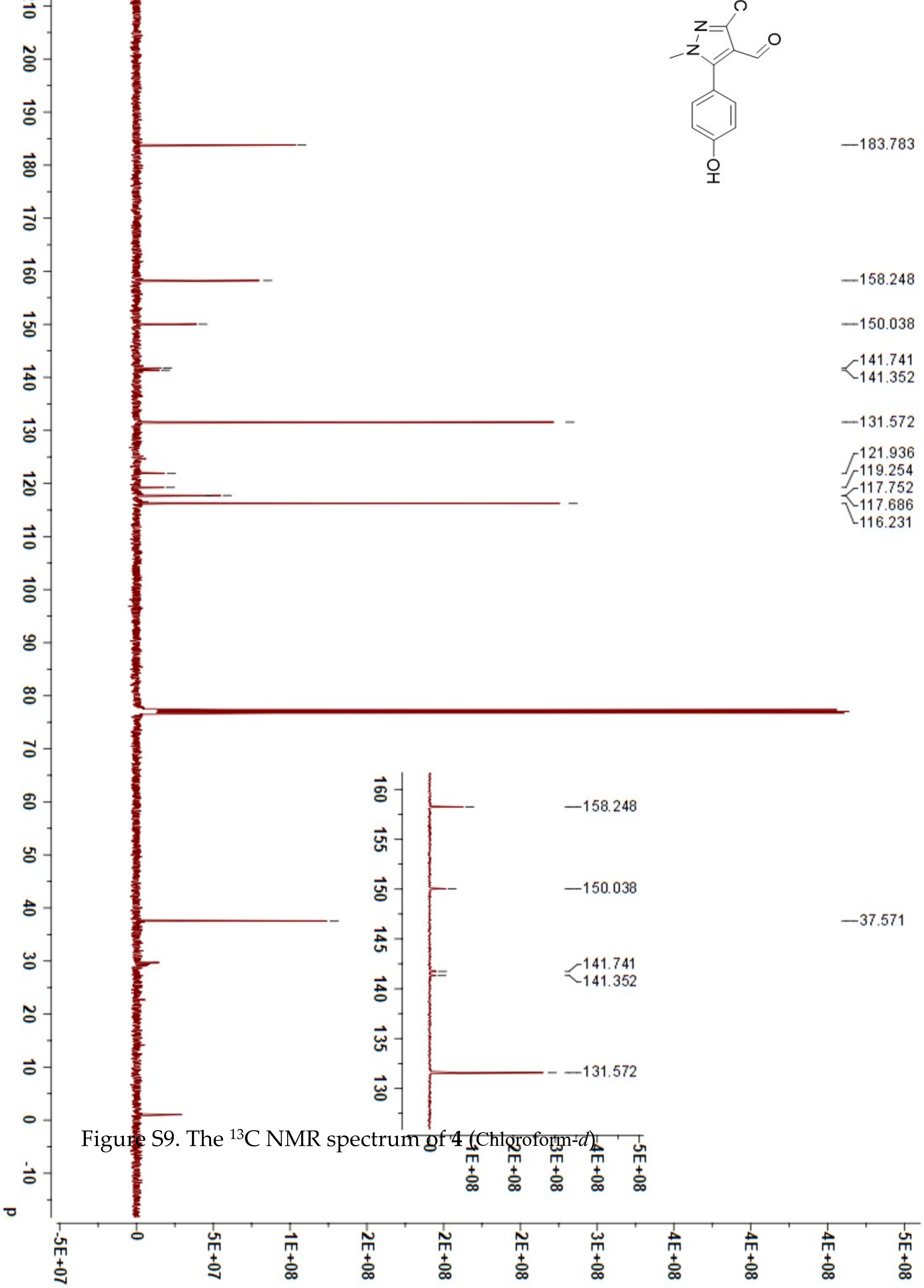

Figure S9. The ^{13}C NMR spectrum of **4** (Chloroform-*d*)

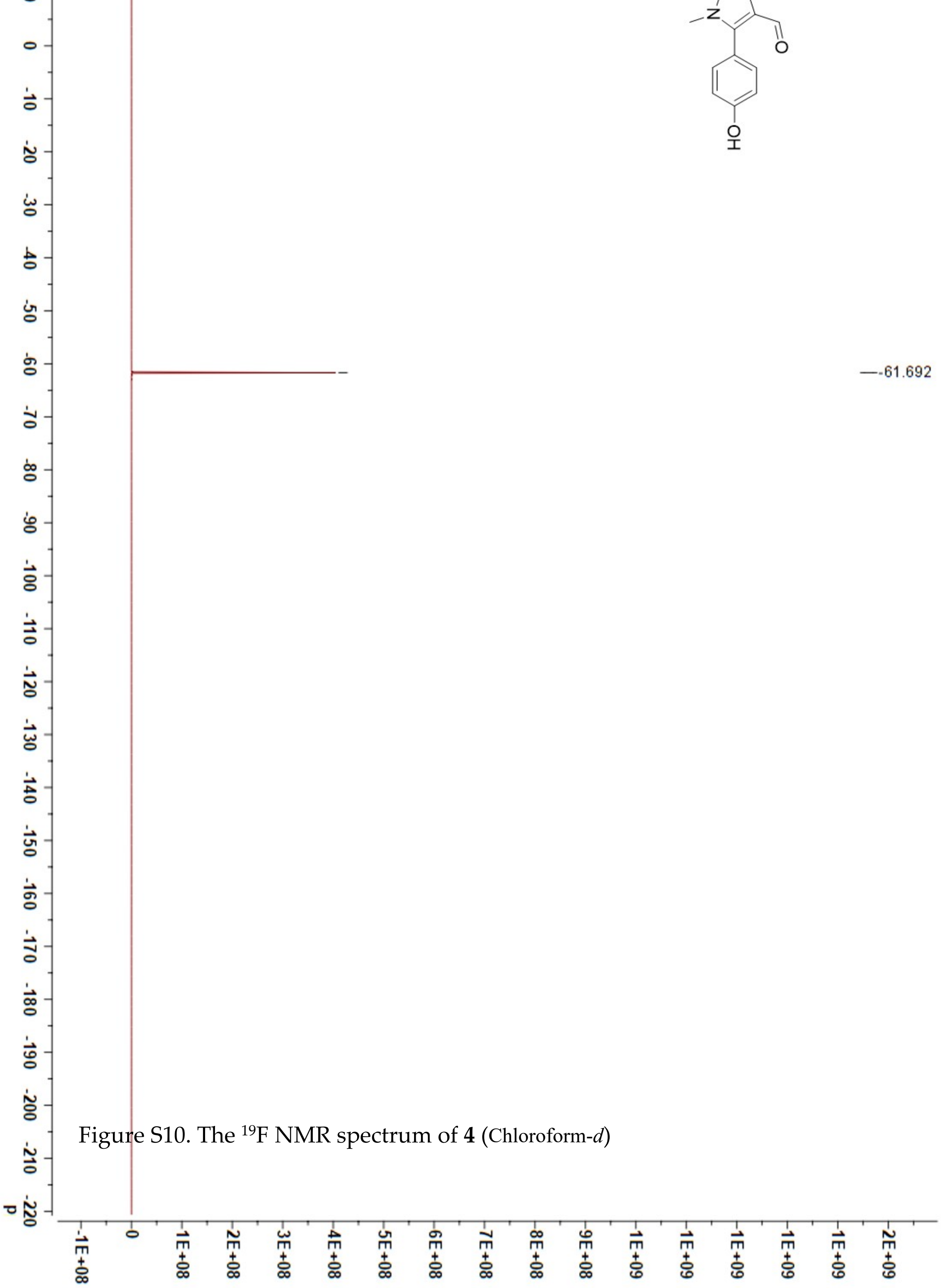

Figure S10. The ^{19}F NMR spectrum of **4** (Chloroform-*d*)

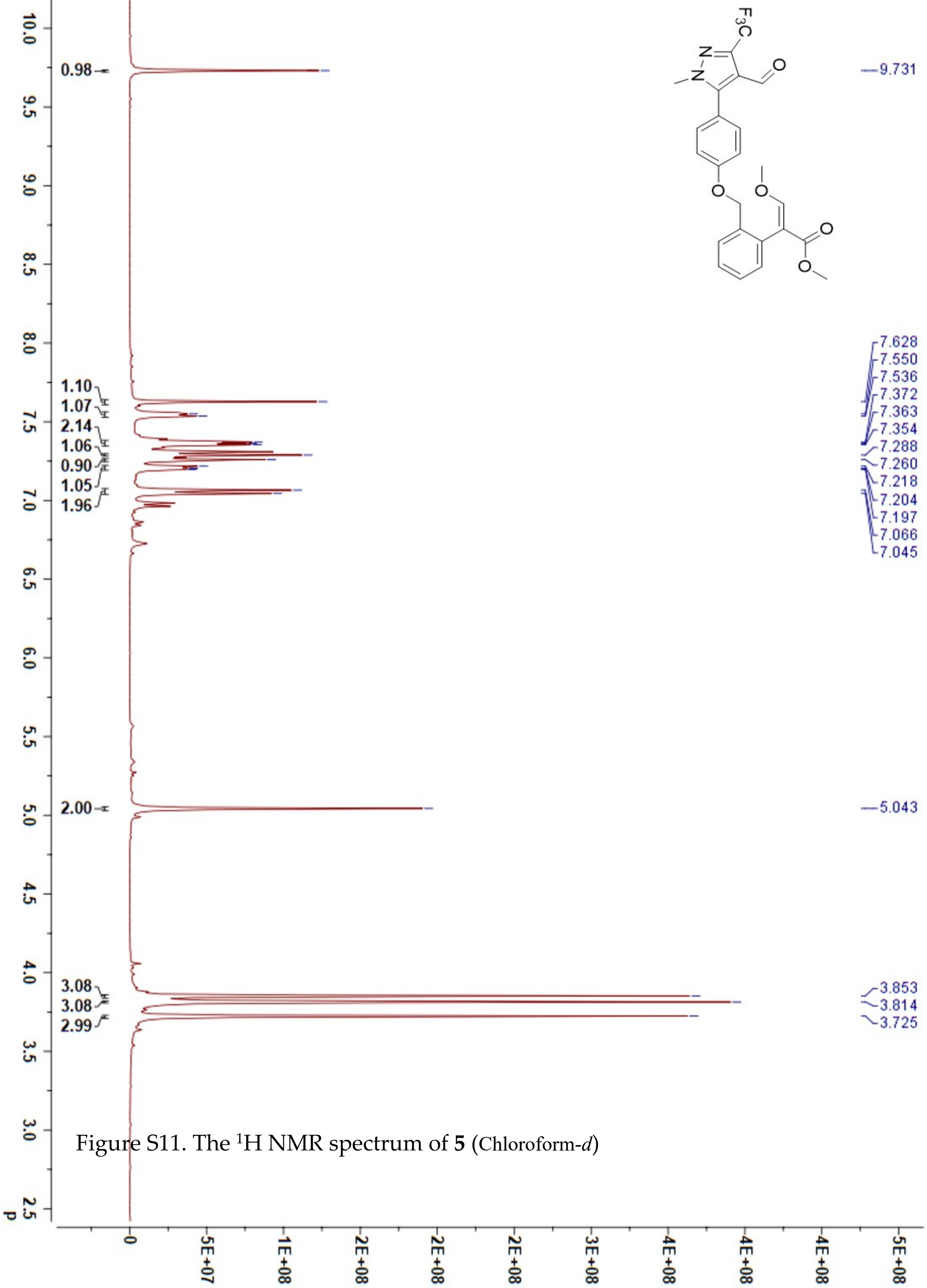

Figure S11. The ^{1}H NMR spectrum of **5** (Chloroform-*d*)

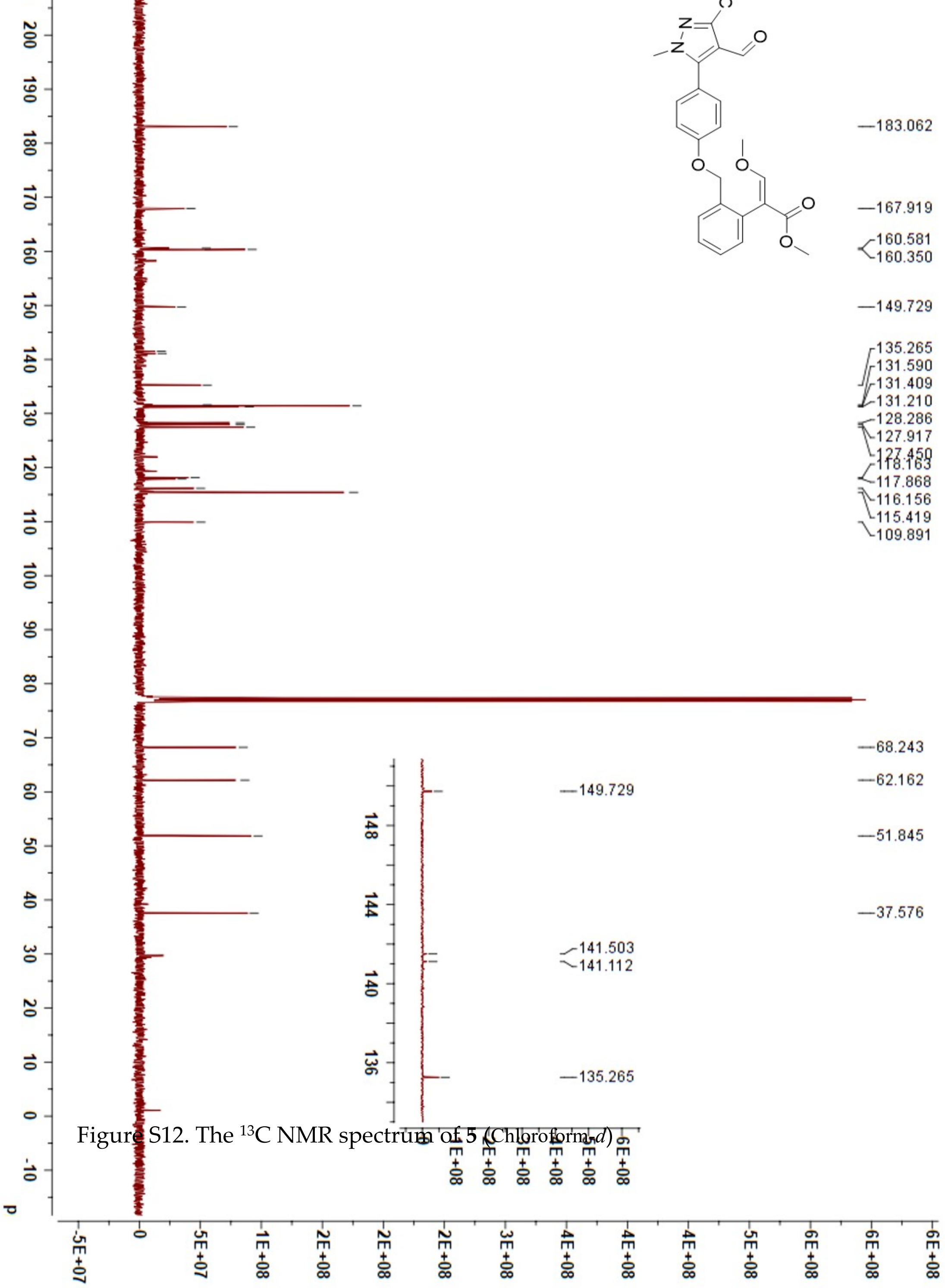

Figure S12. The ^{13}C NMR spectrum of **5** (Chloroform-*d*)

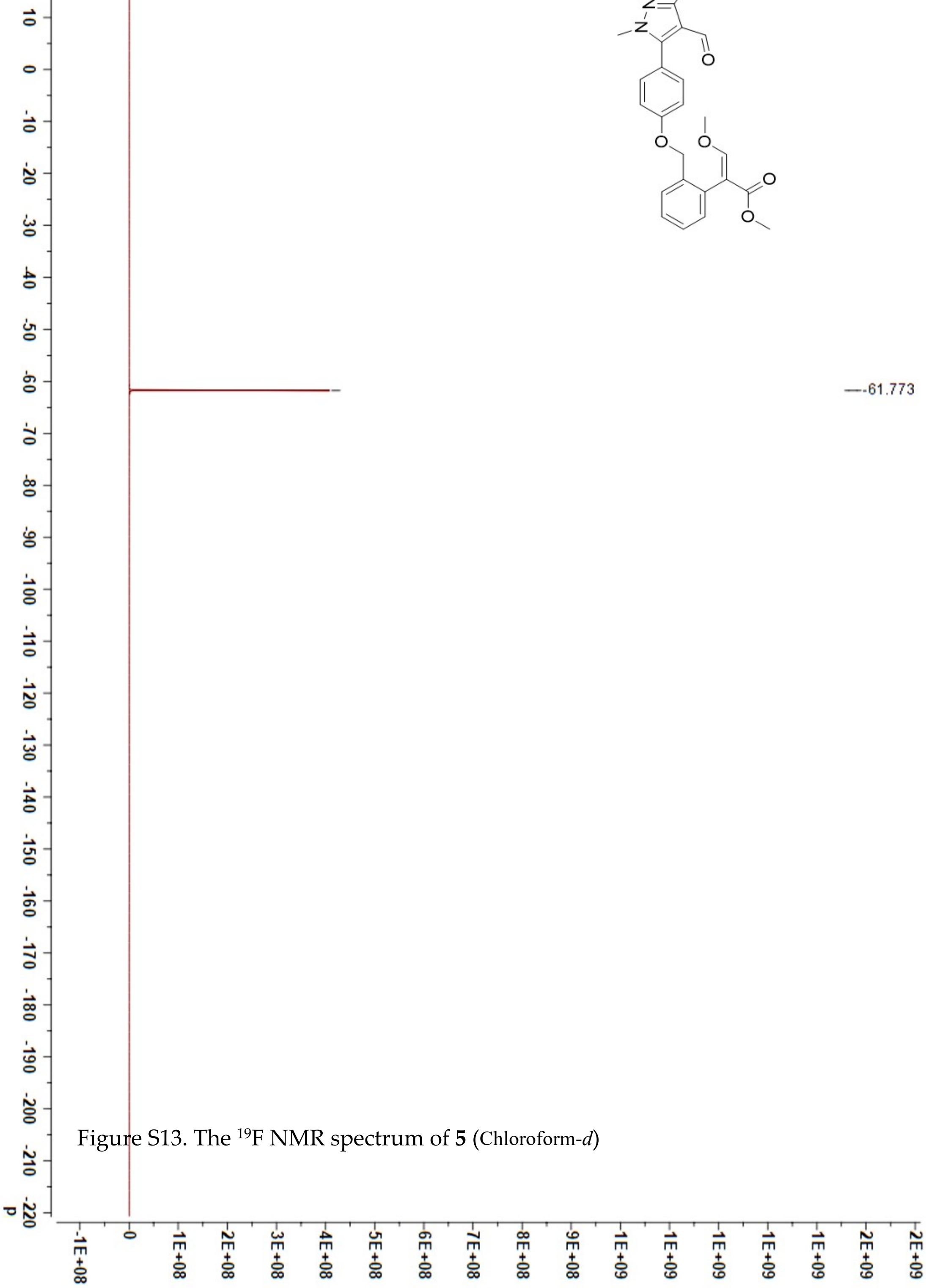

Figure S13. The ^{19}F NMR spectrum of **5** (Chloroform-*d*)

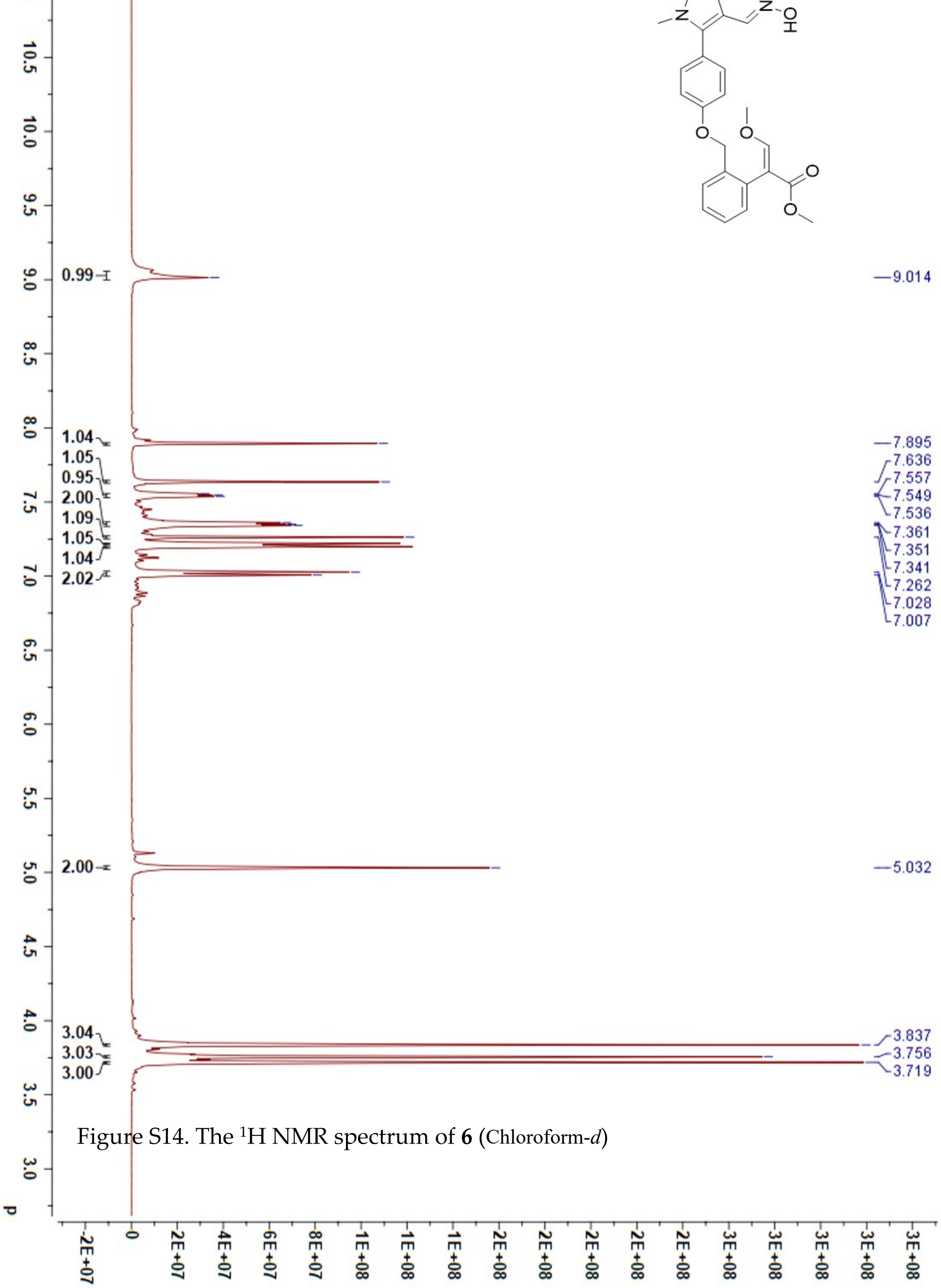

Figure S14. The ^{1}H NMR spectrum of **6** (Chloroform-*d*)

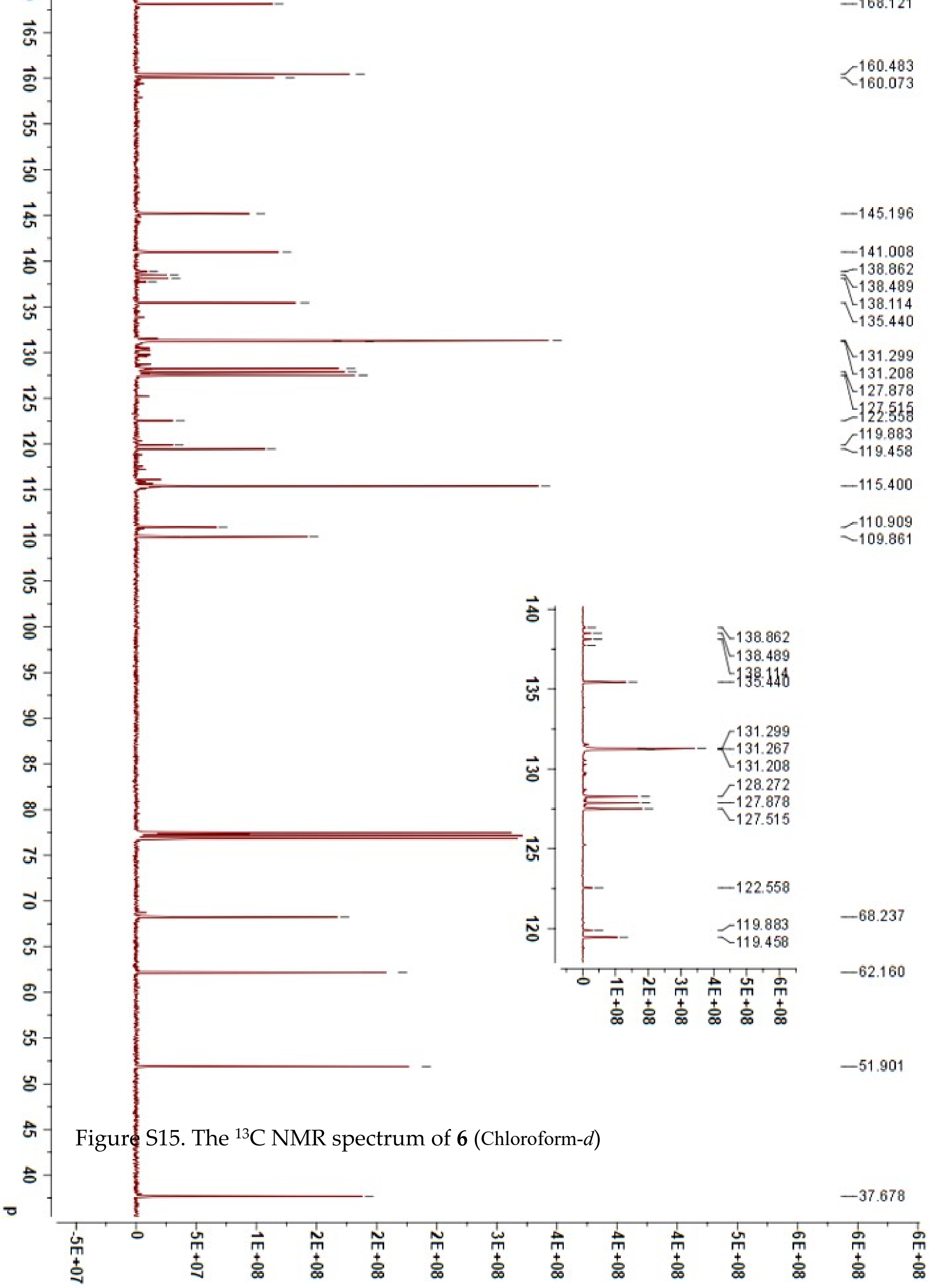

Figure S15. The ^{13}C NMR spectrum of **6** (Chloroform-*d*)

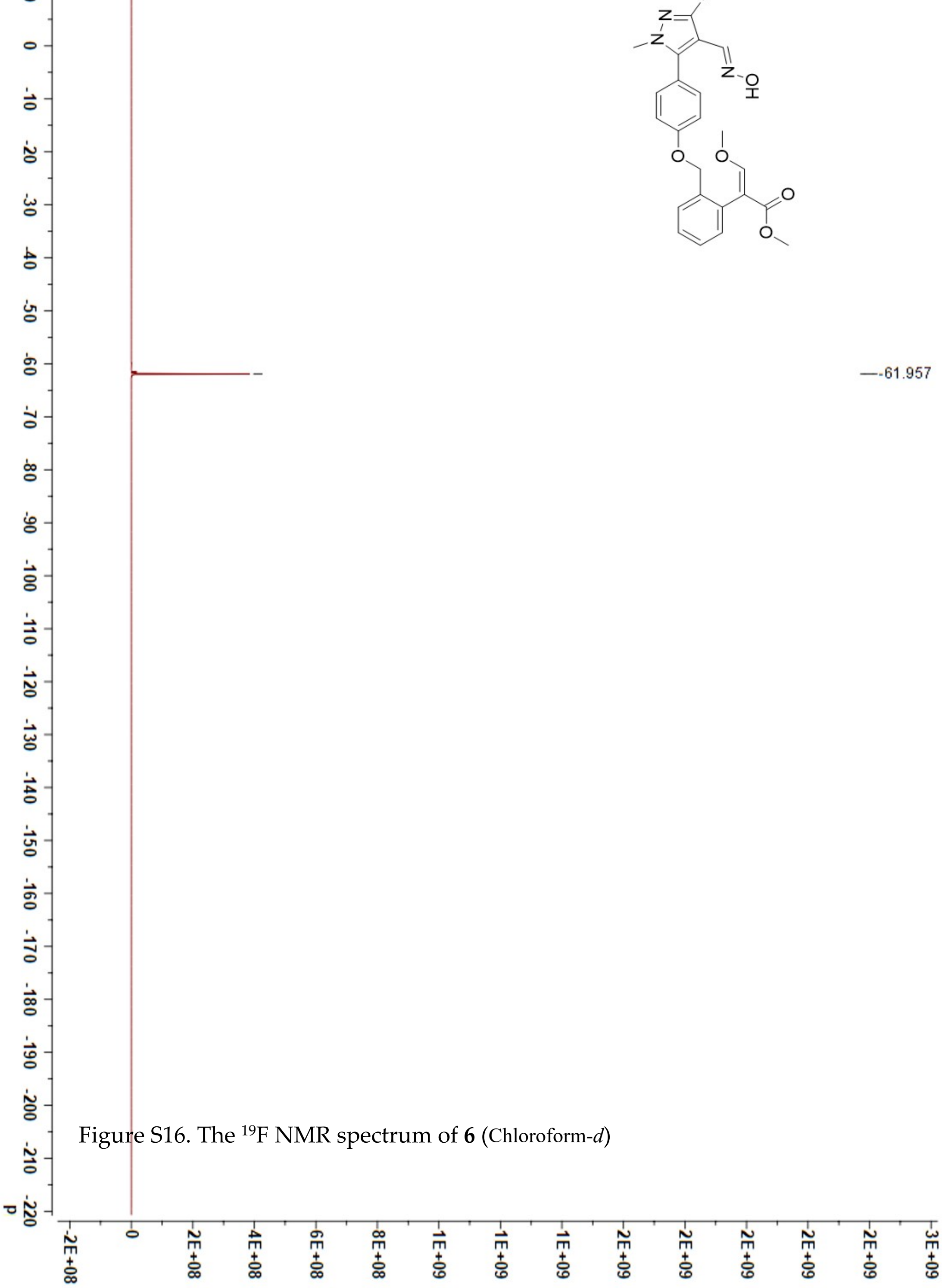

Figure S16. The ^{19}F NMR spectrum of **6** (Chloroform-*d*)

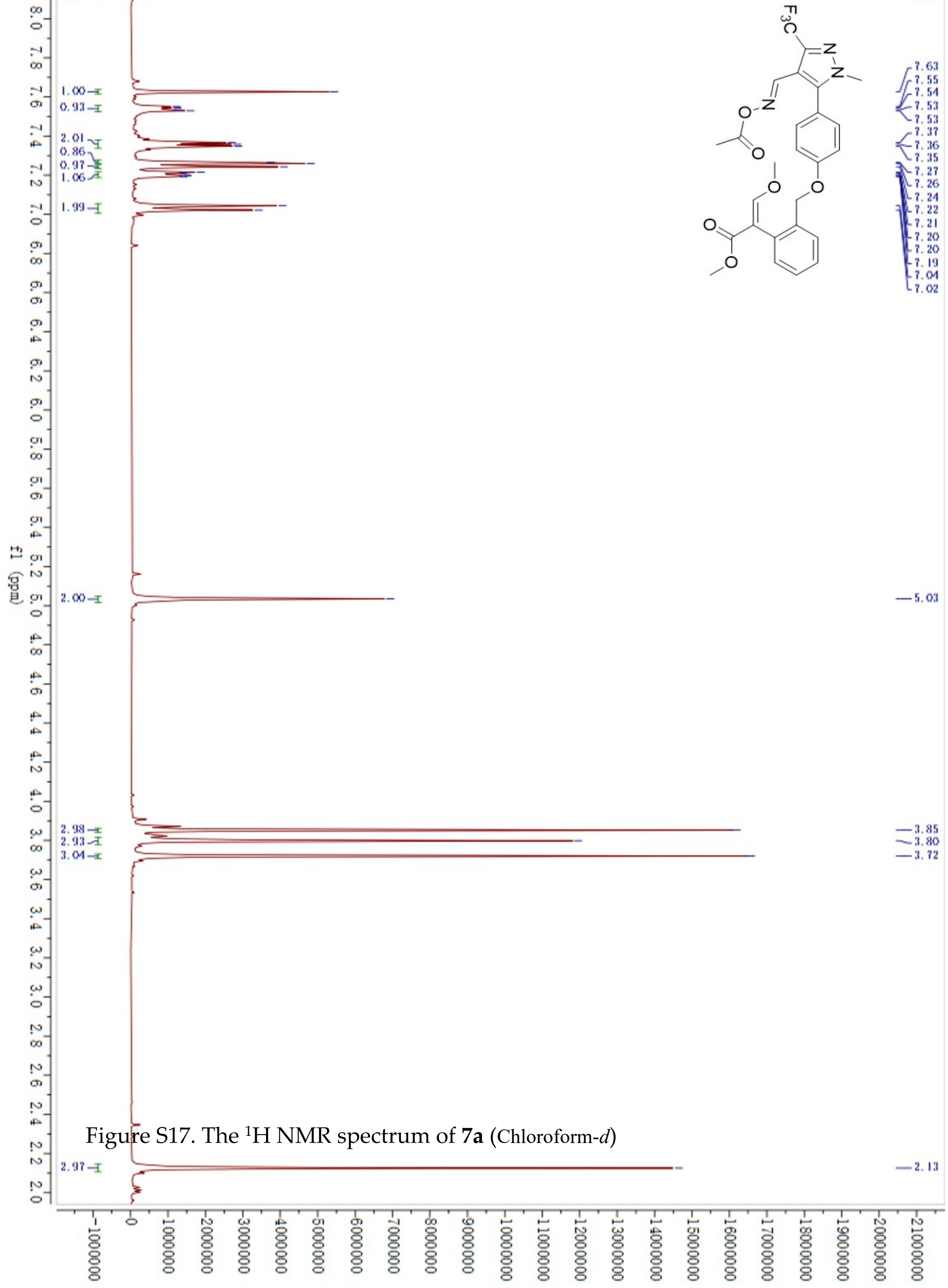

Figure S17. The ^{1}H NMR spectrum of **7a** (Chloroform-*d*)

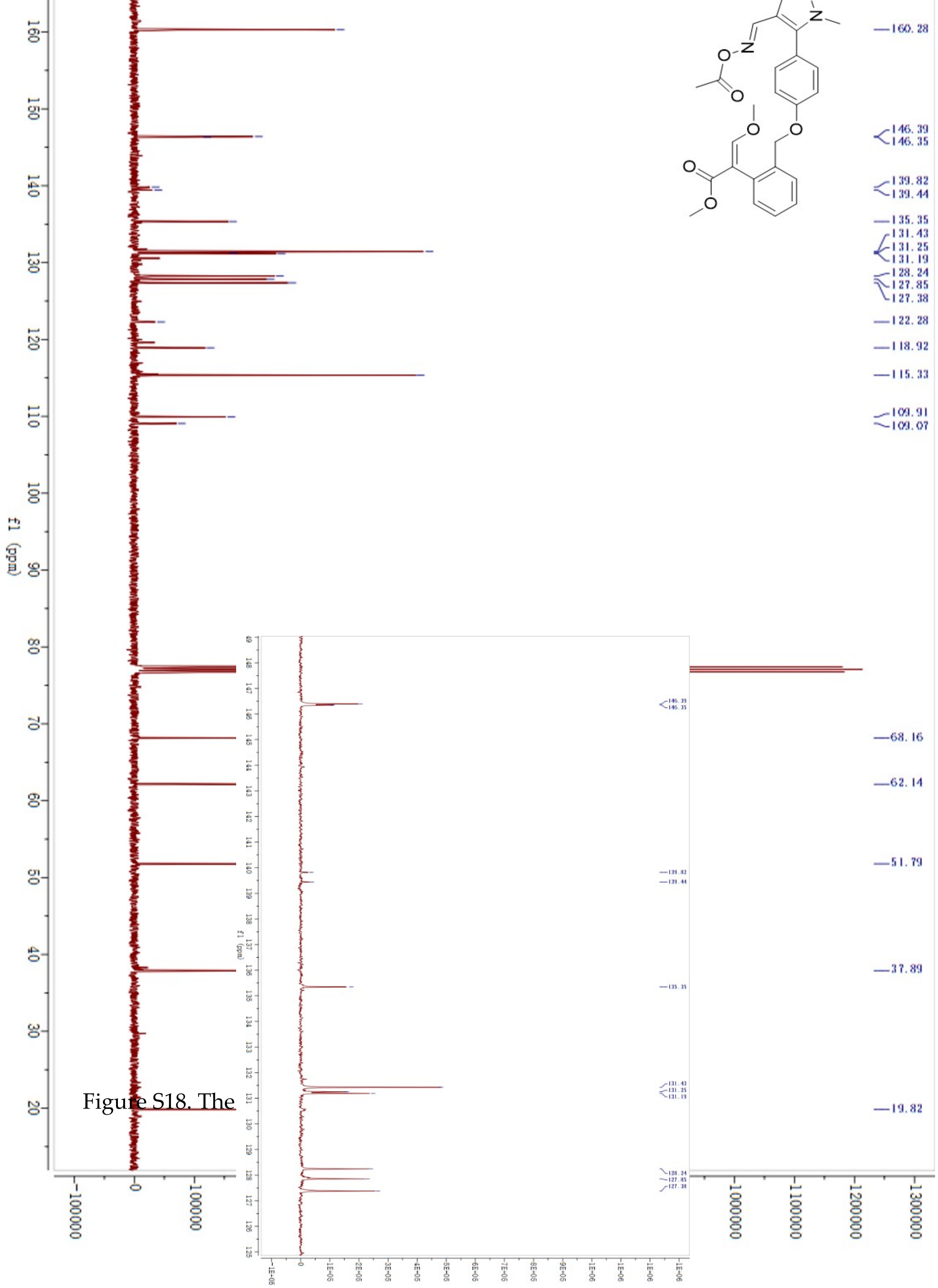

Figure S18. The

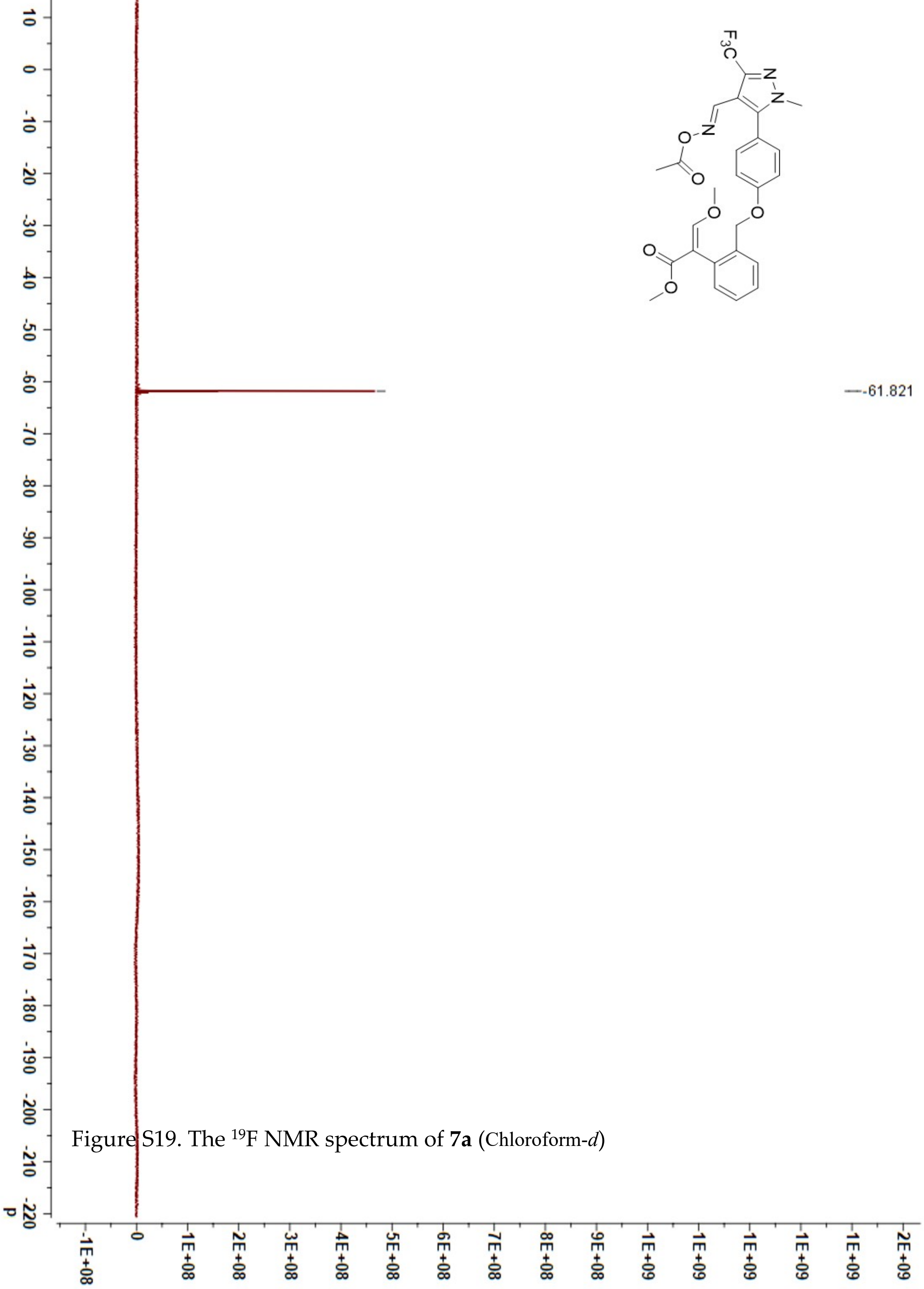

Figure S19. The ^{19}F NMR spectrum of **7a** (Chloroform-*d*)

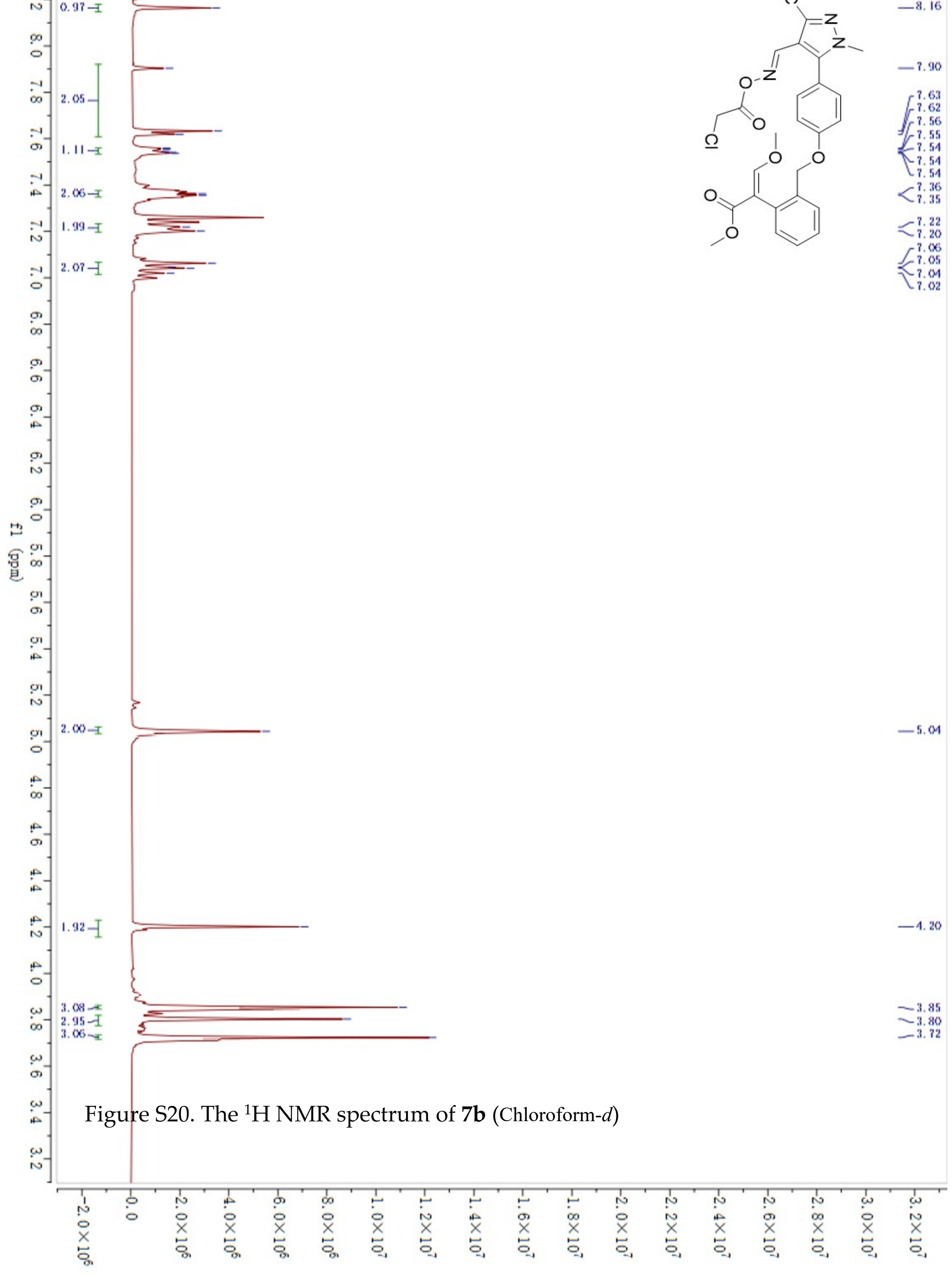

Figure S20. The ¹H NMR spectrum of **7b** (Chloroform-*d*)

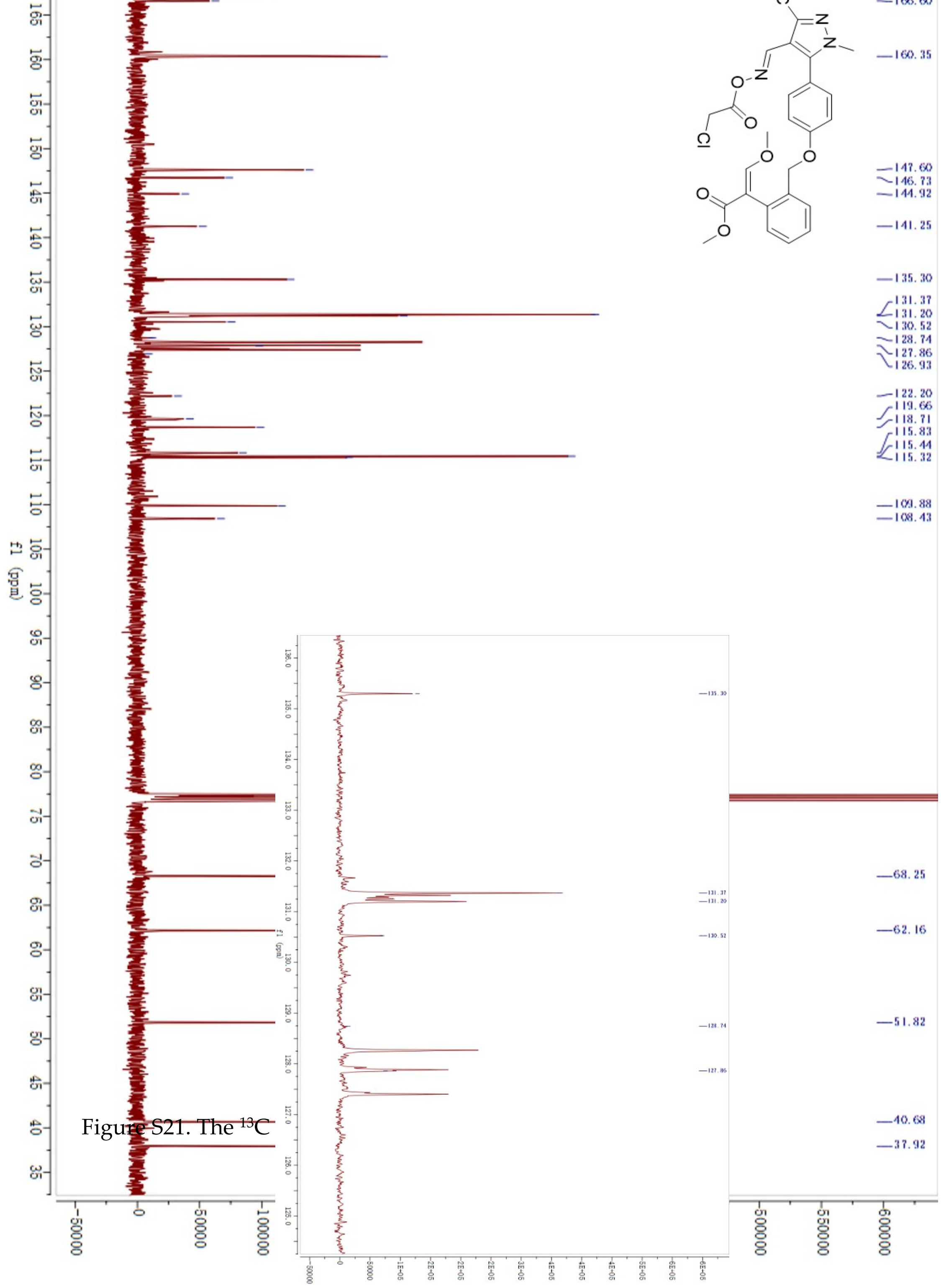

Figure S21. The ^{13}C

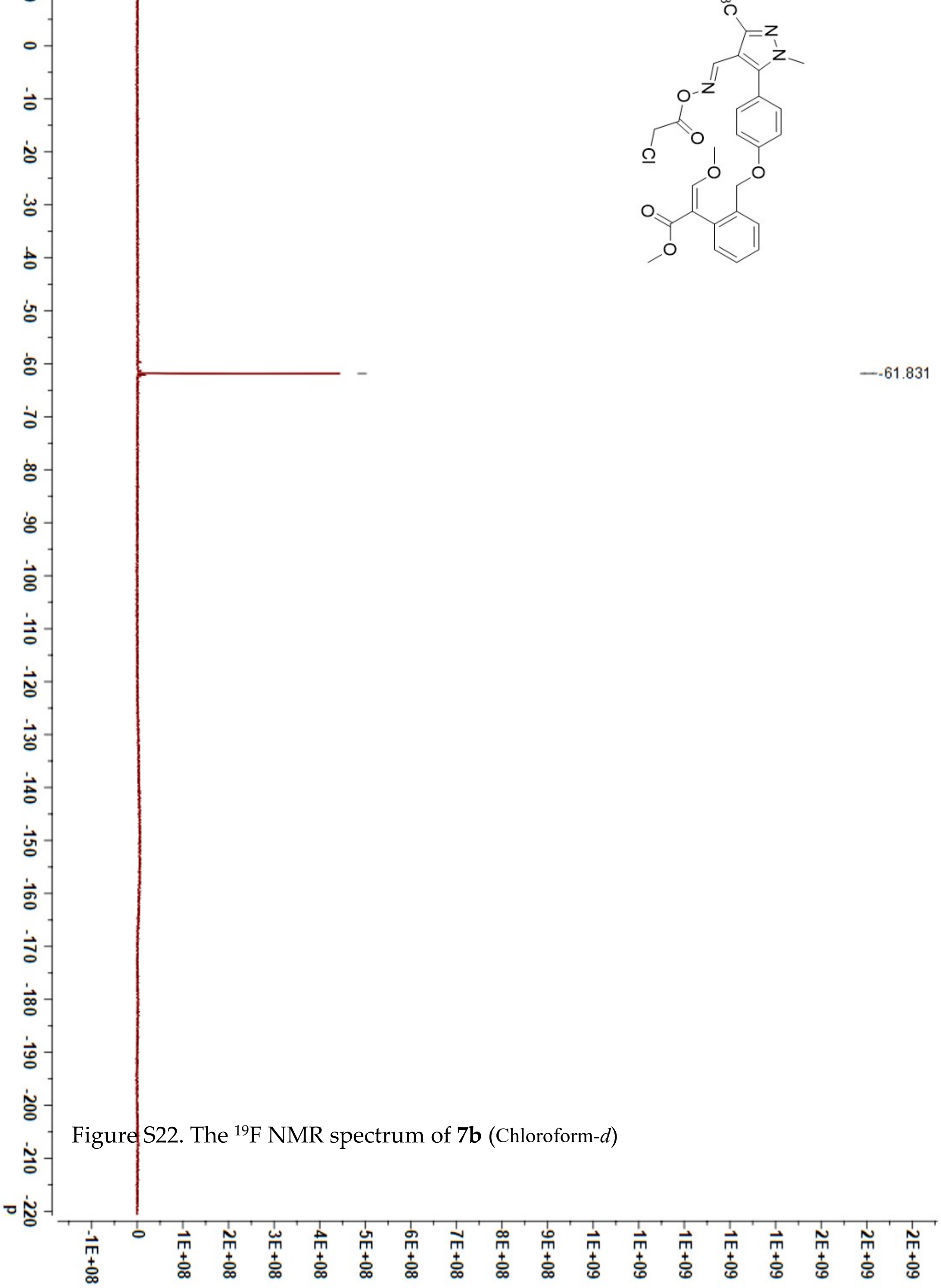

Figure S22. The ^{19}F NMR spectrum of **7b** (Chloroform-*d*)

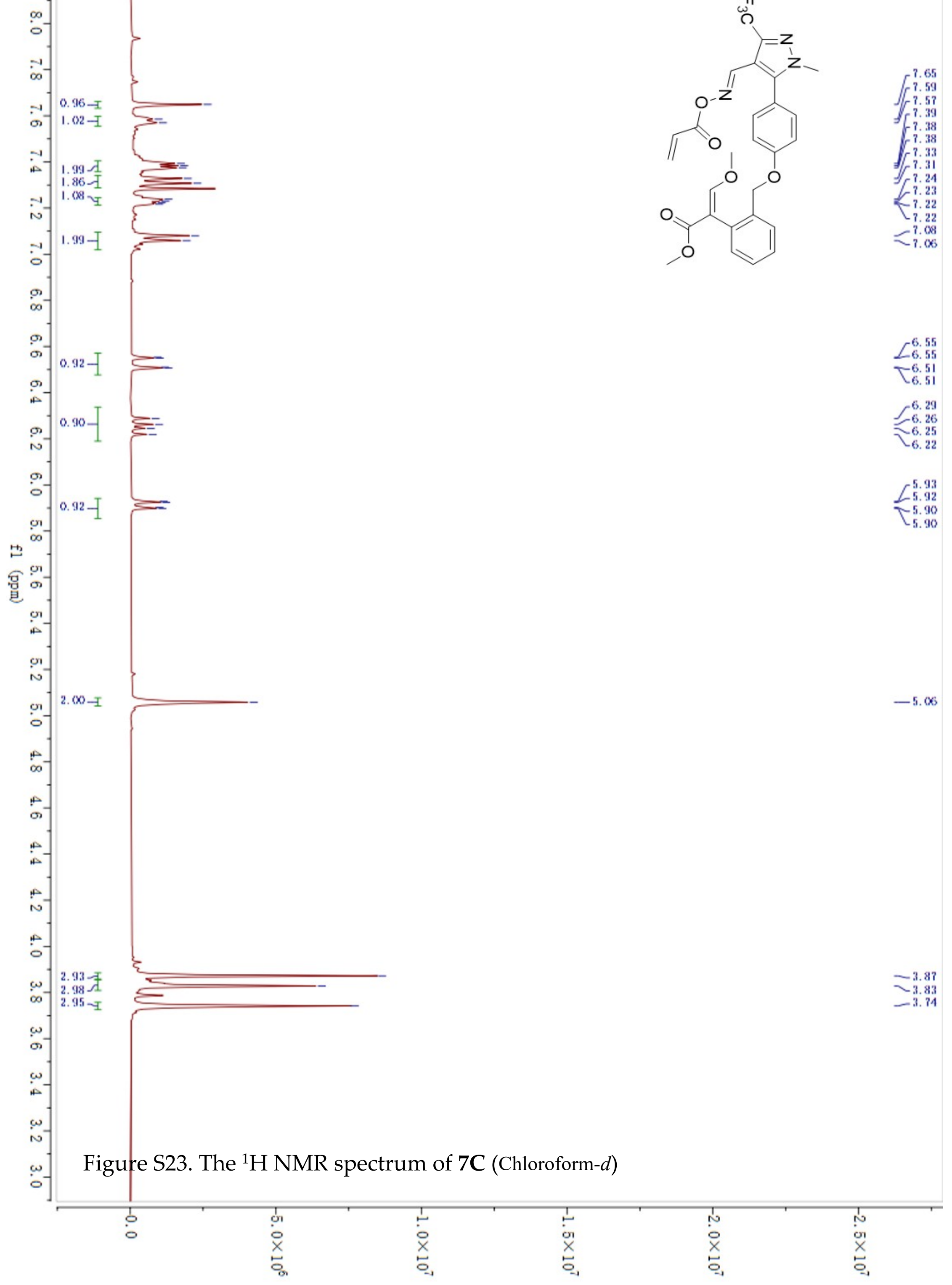

Figure S23. The ^{1}H NMR spectrum of **7C** (Chloroform-*d*)

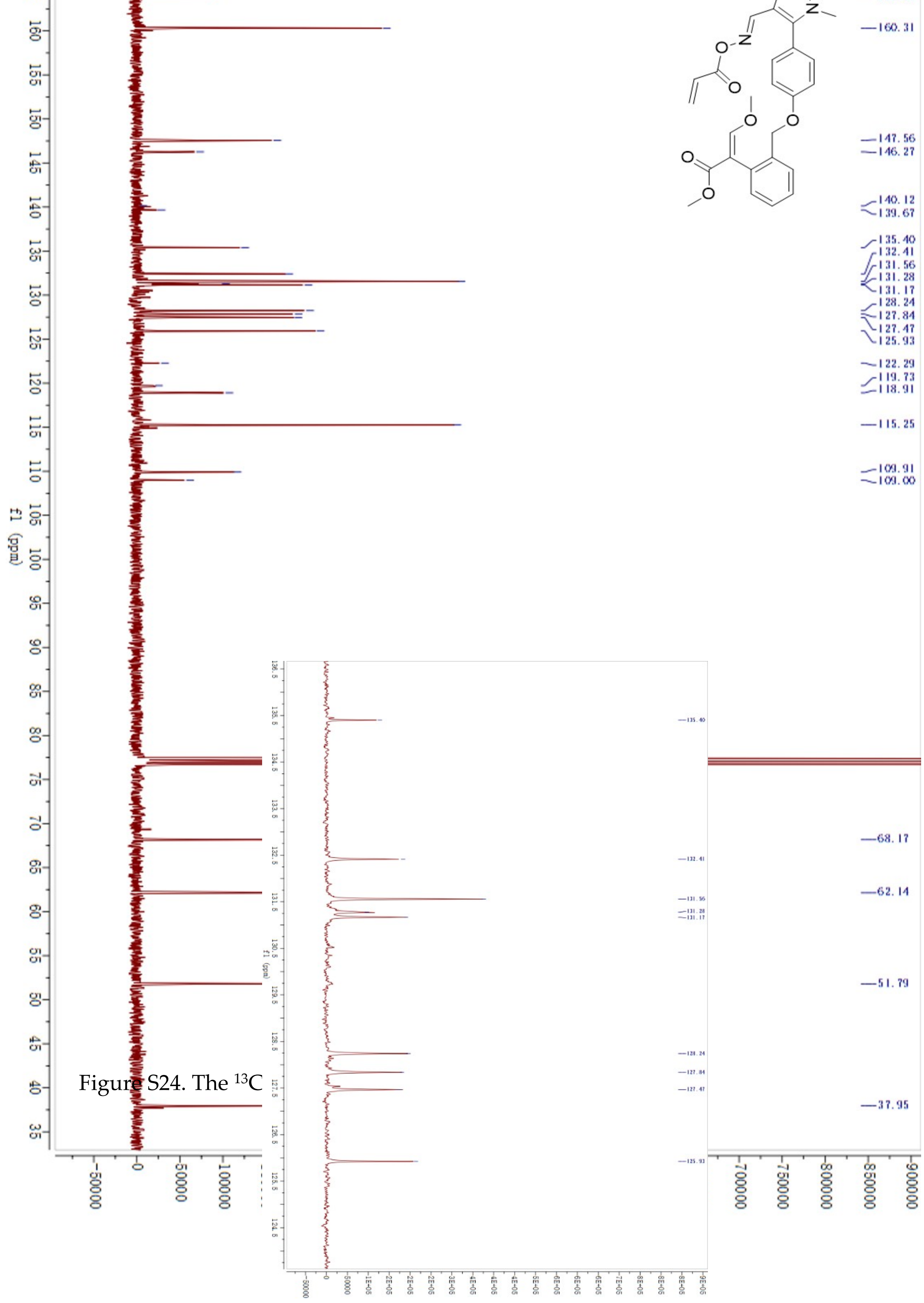

Figure S24. The ^{13}C

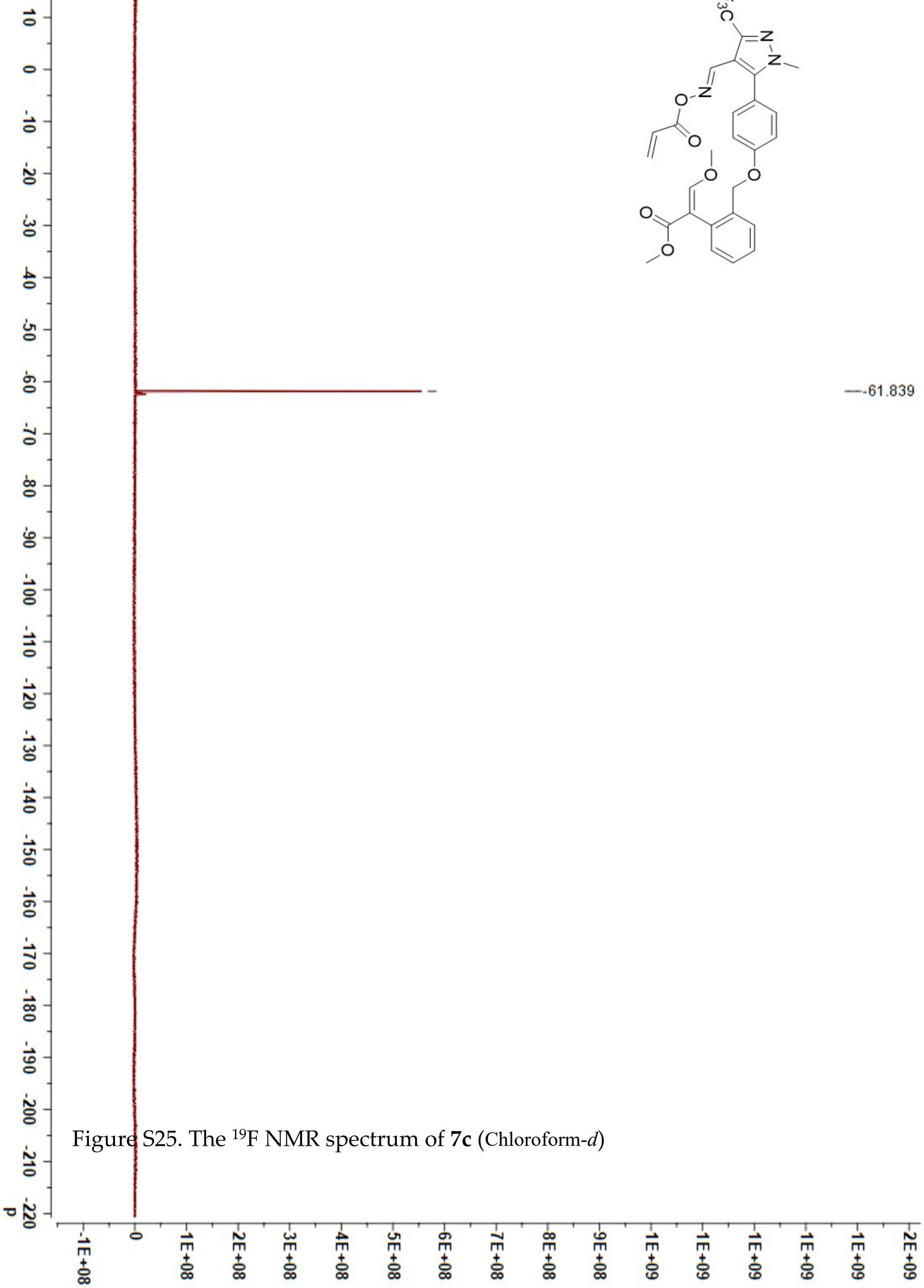

Figure S25. The ^{19}F NMR spectrum of **7c** (Chloroform-*d*)

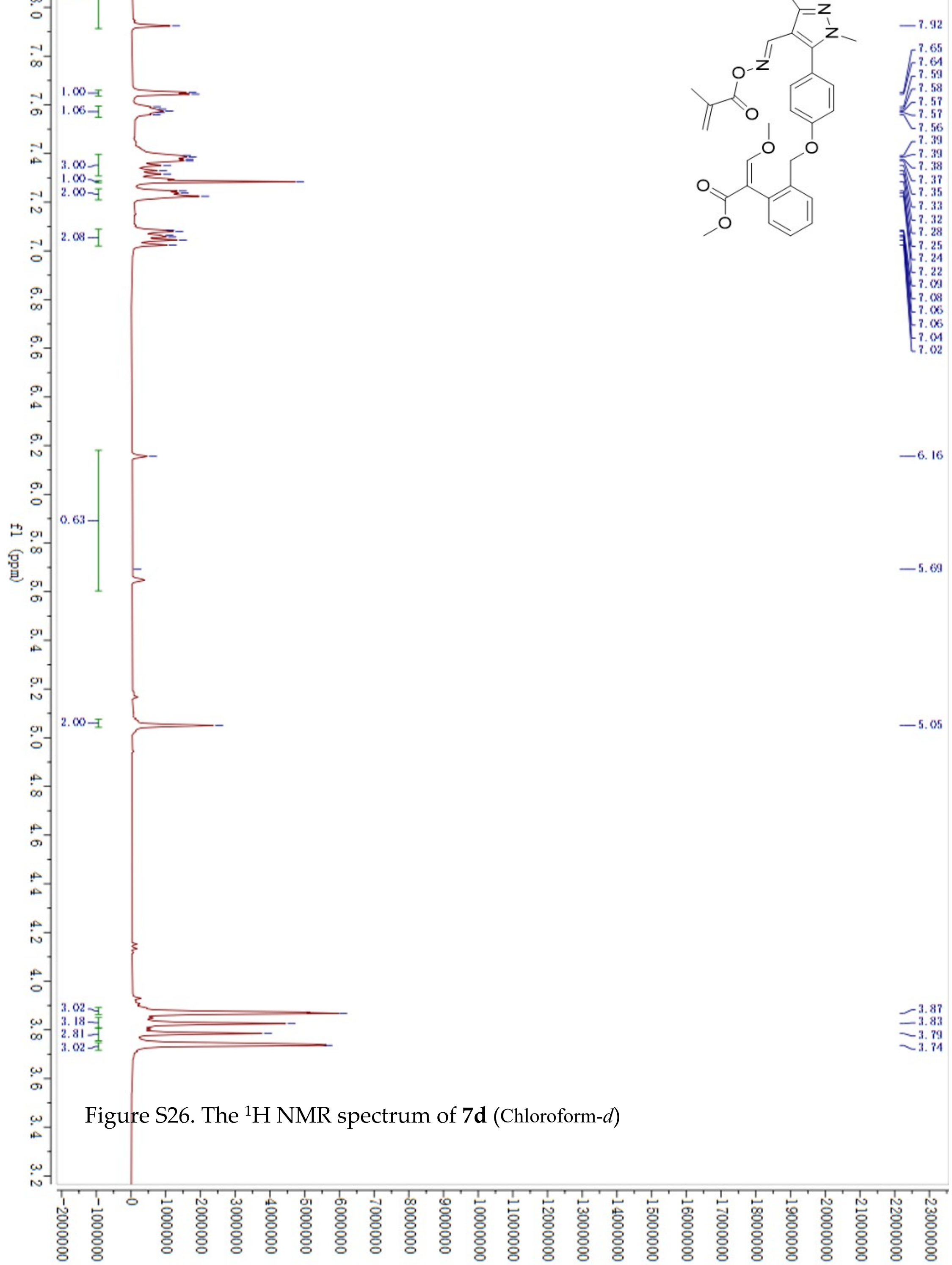

Figure S26. The ^{1}H NMR spectrum of **7d** (Chloroform-*d*)

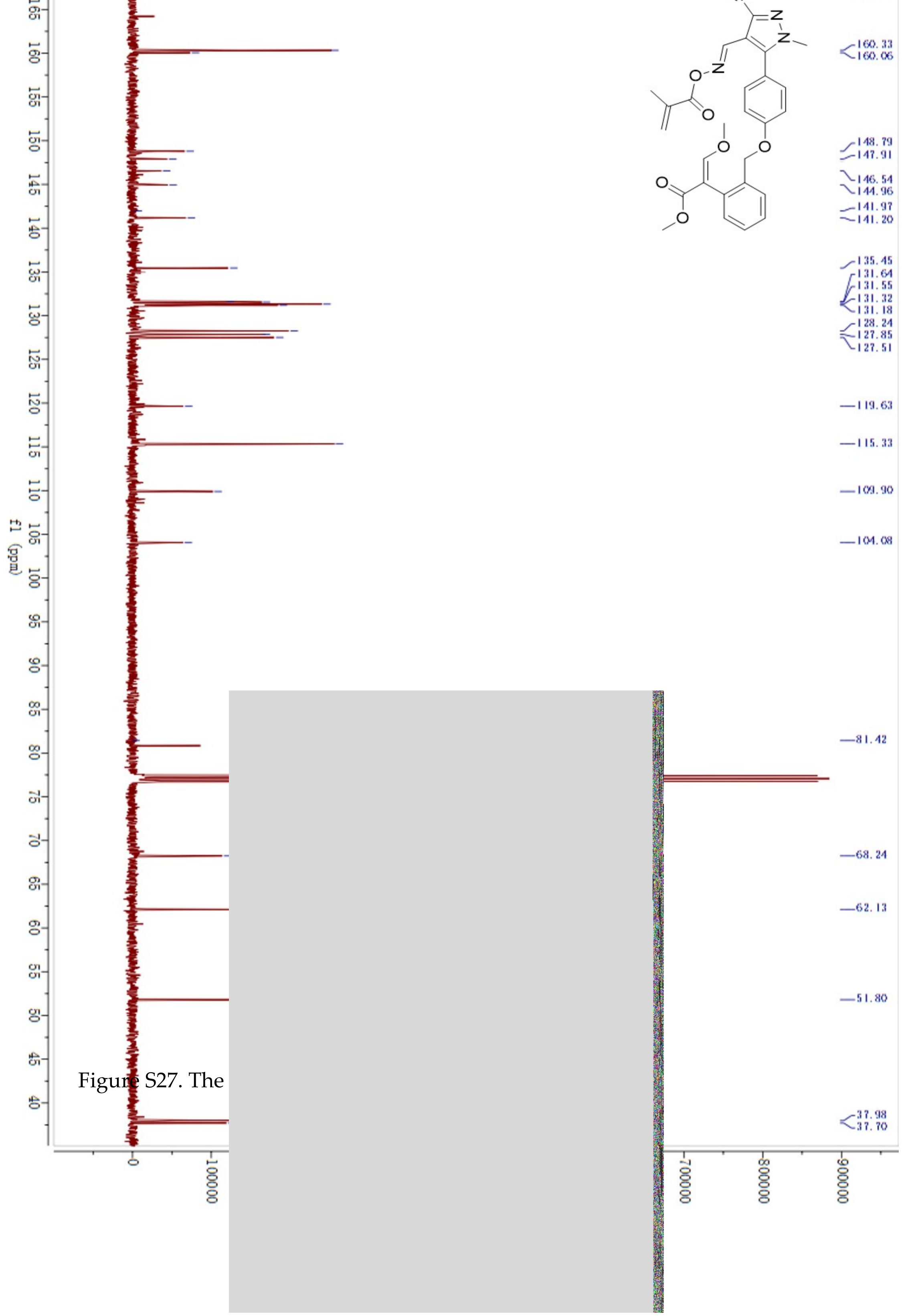

Figure S27. The [illegible]

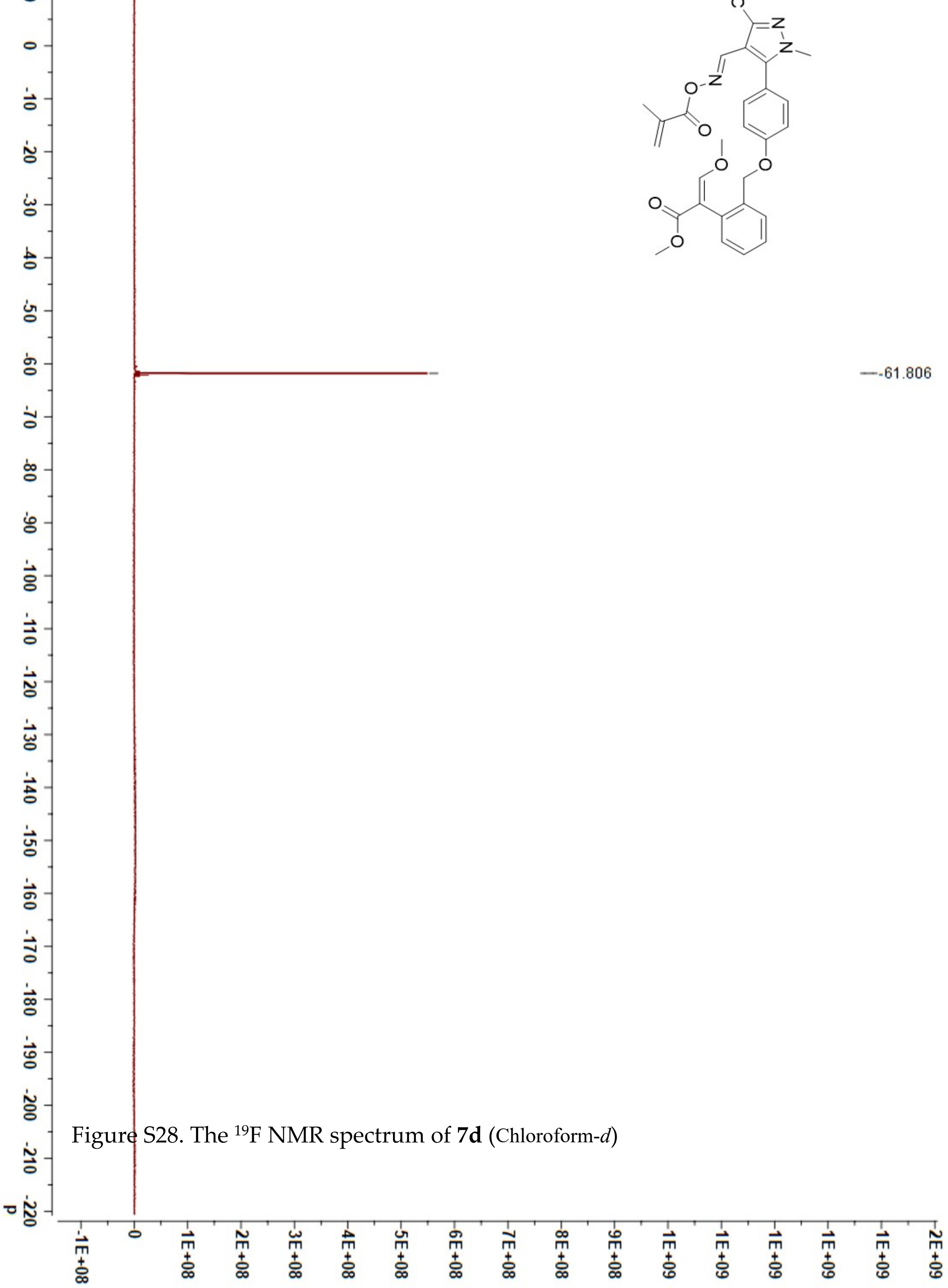

Figure S28. The ^{19}F NMR spectrum of **7d** (Chloroform-*d*)

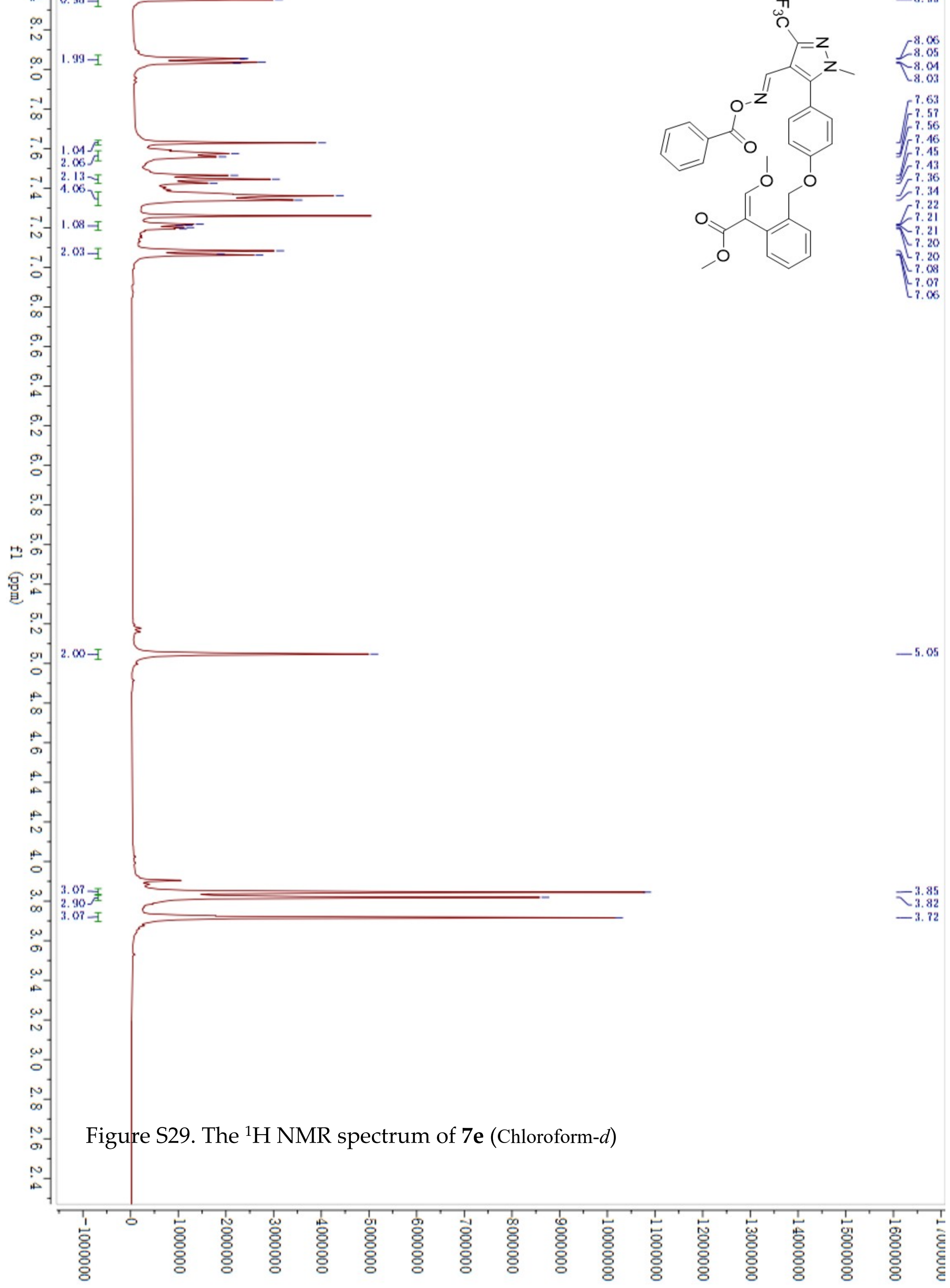

Figure S29. The ^{1}H NMR spectrum of **7e** (Chloroform-*d*)

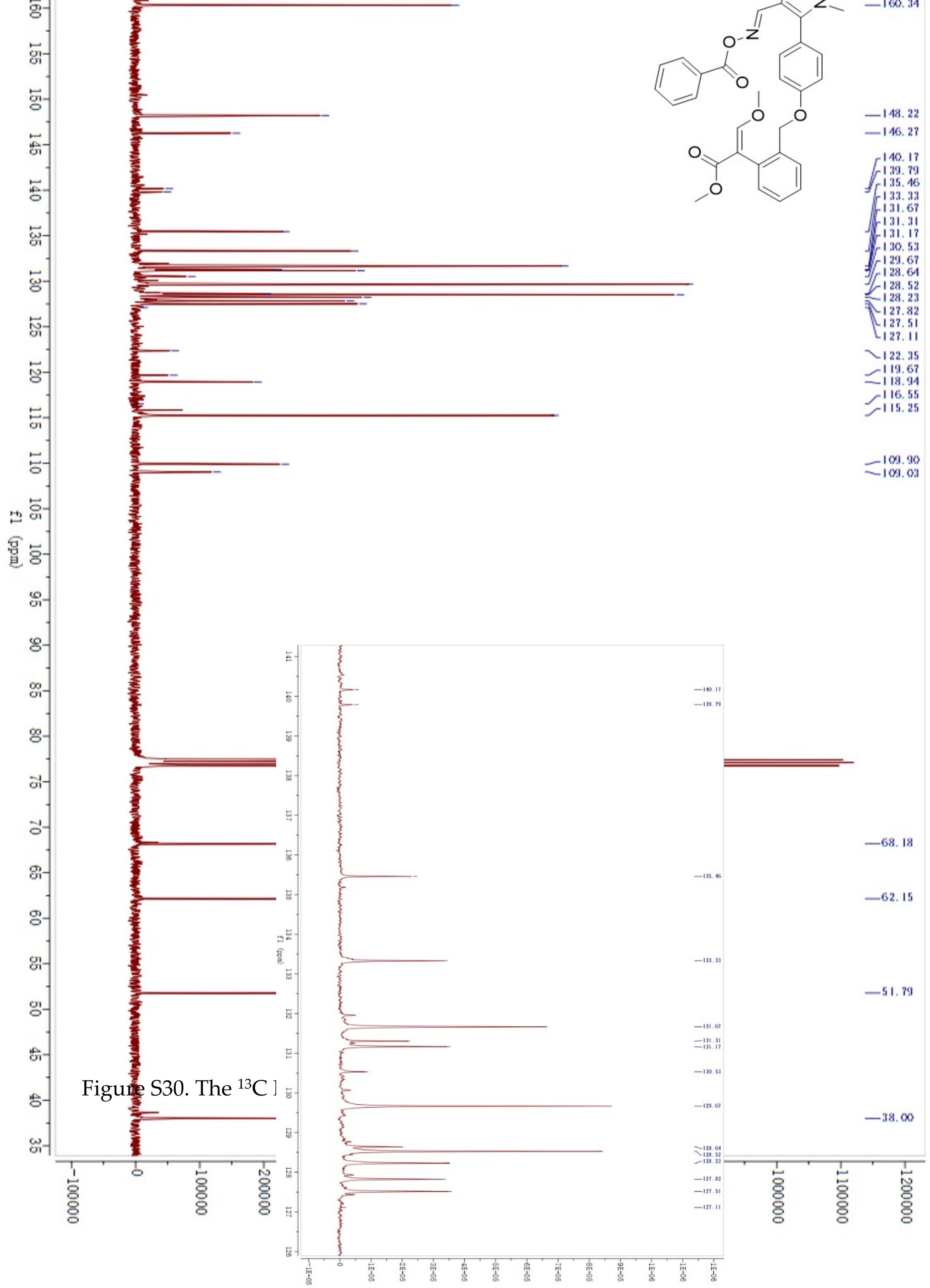

Figure S30. The ^{13}C [illegible]

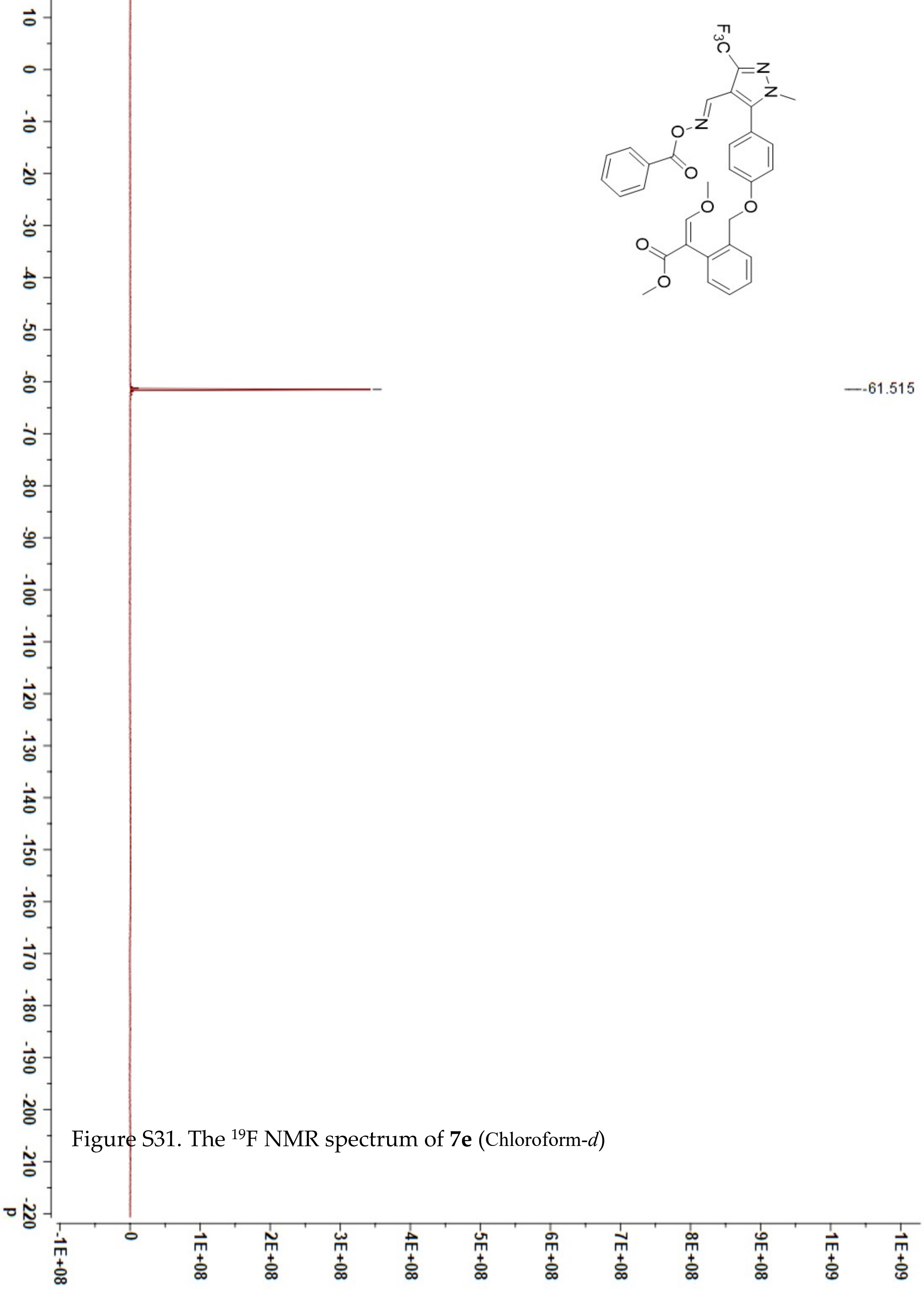

Figure S31. The ^{19}F NMR spectrum of **7e** (Chloroform-*d*)

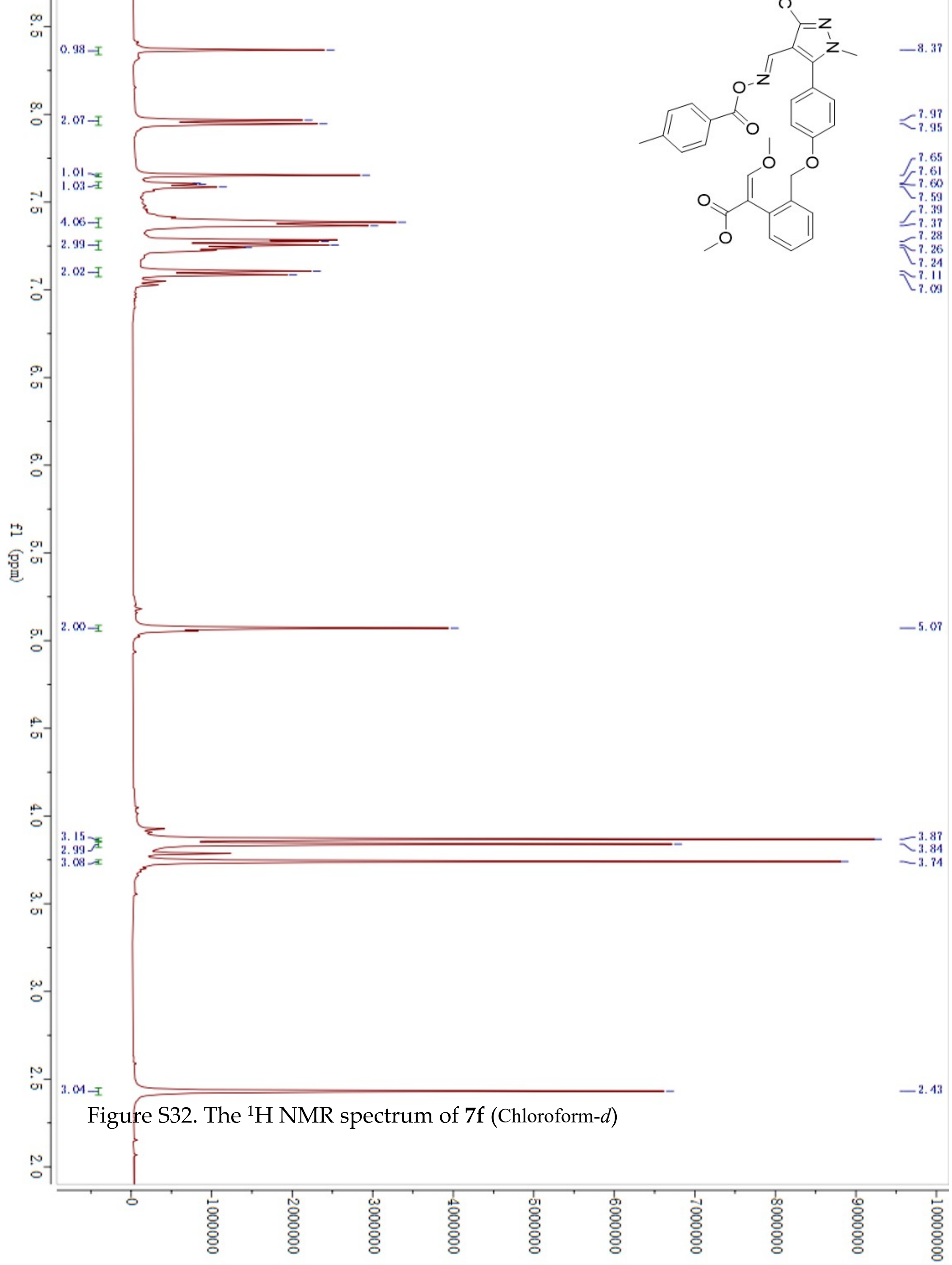

Figure S32. The ^{1}H NMR spectrum of **7f** (Chloroform-*d*)

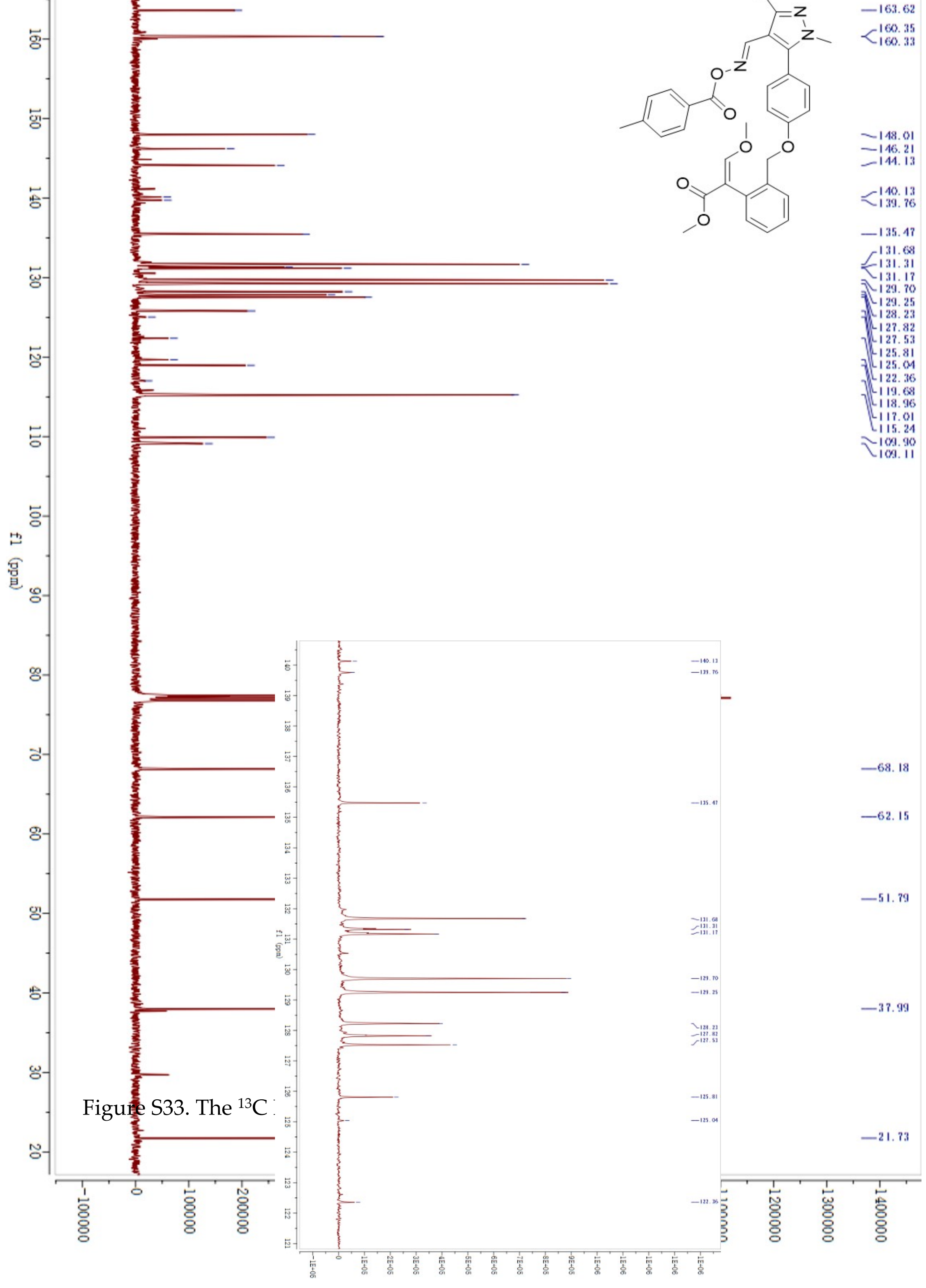

Figure S33. The ^{13}C [illegible]

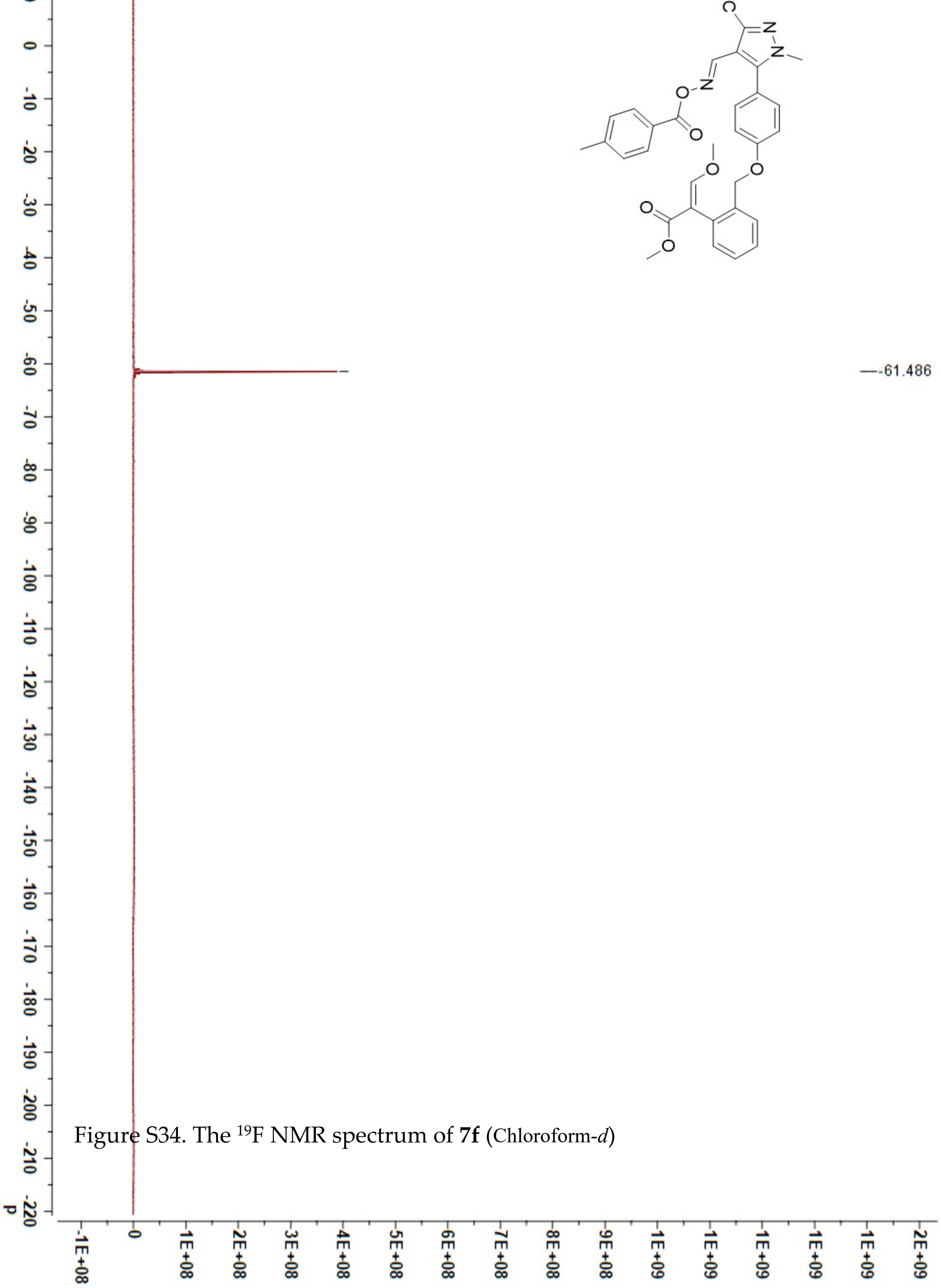

Figure S34. The ^{19}F NMR spectrum of **7f** (Chloroform-*d*)

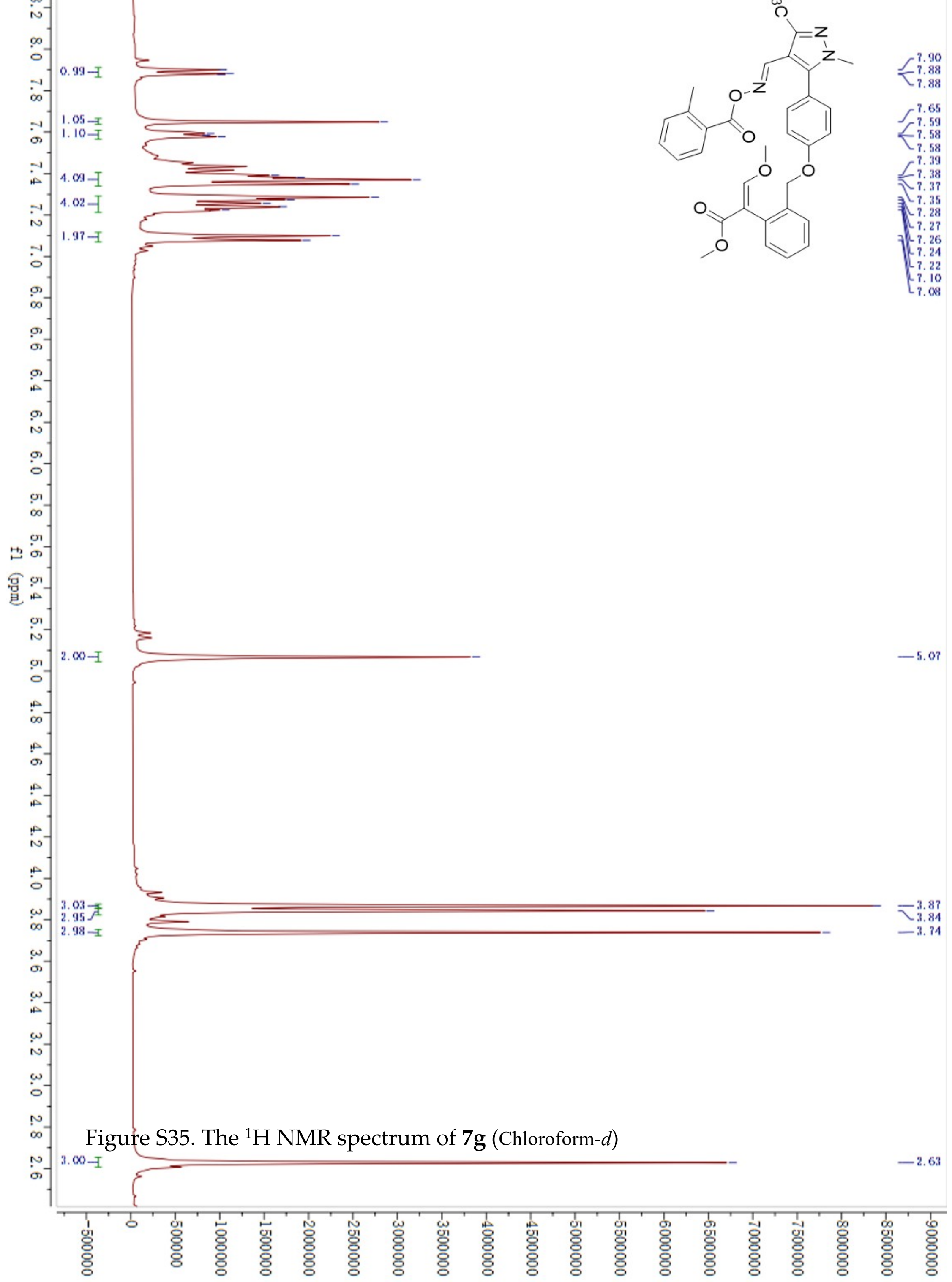

Figure S35. The ^{1}H NMR spectrum of **7g** (Chloroform-*d*)

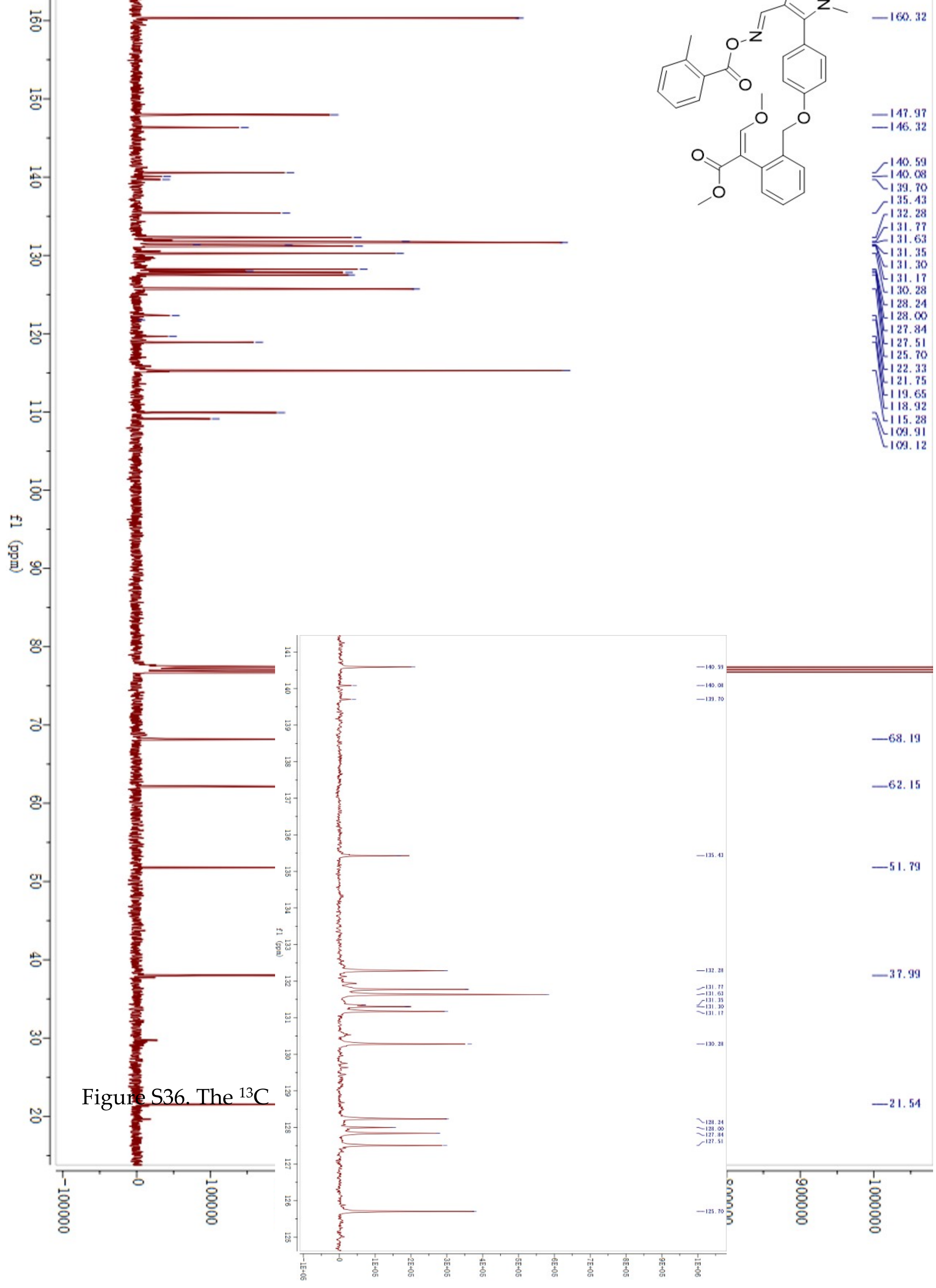

Figure S36. The ^{13}C

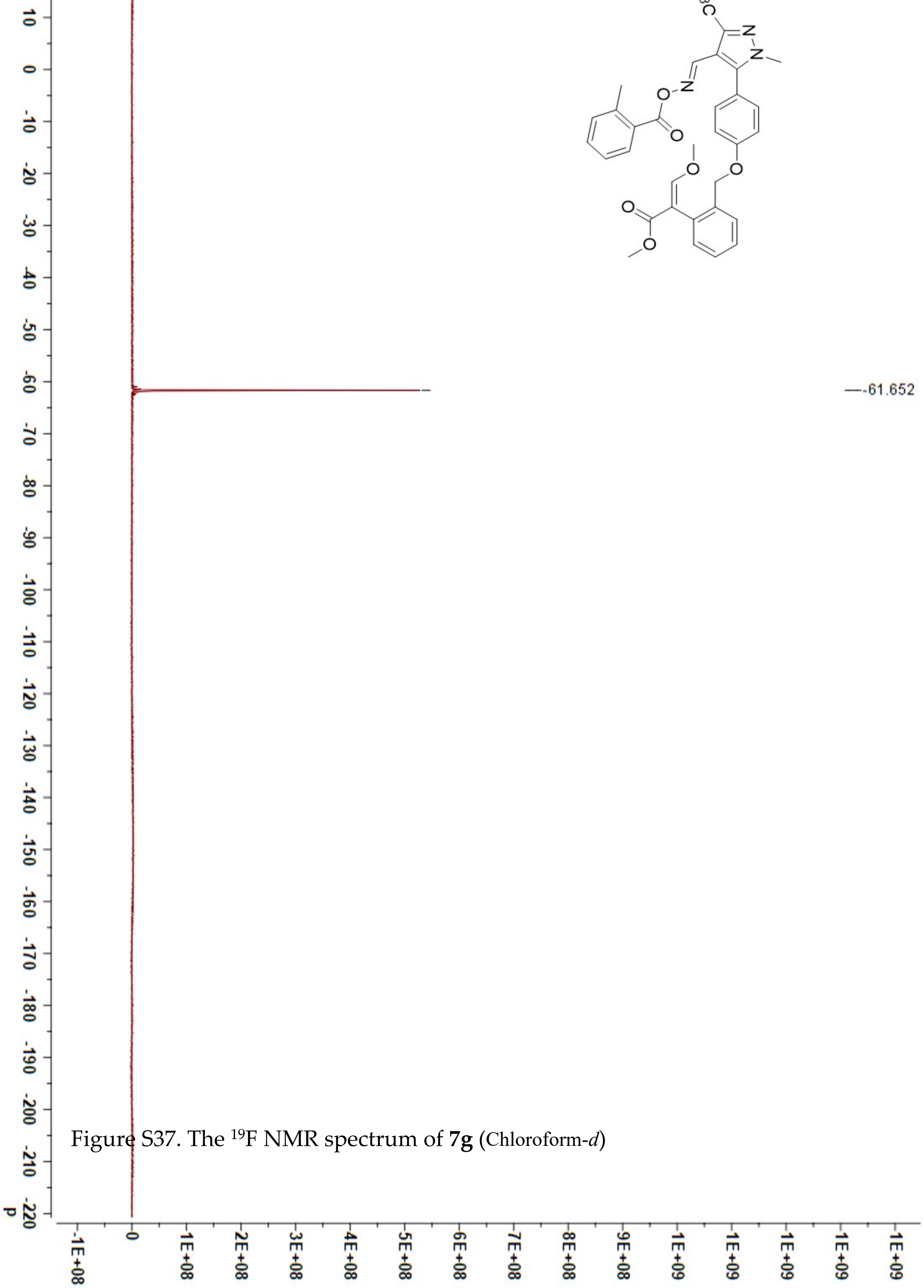

Figure S37. The ^{19}F NMR spectrum of **7g** (Chloroform-*d*)

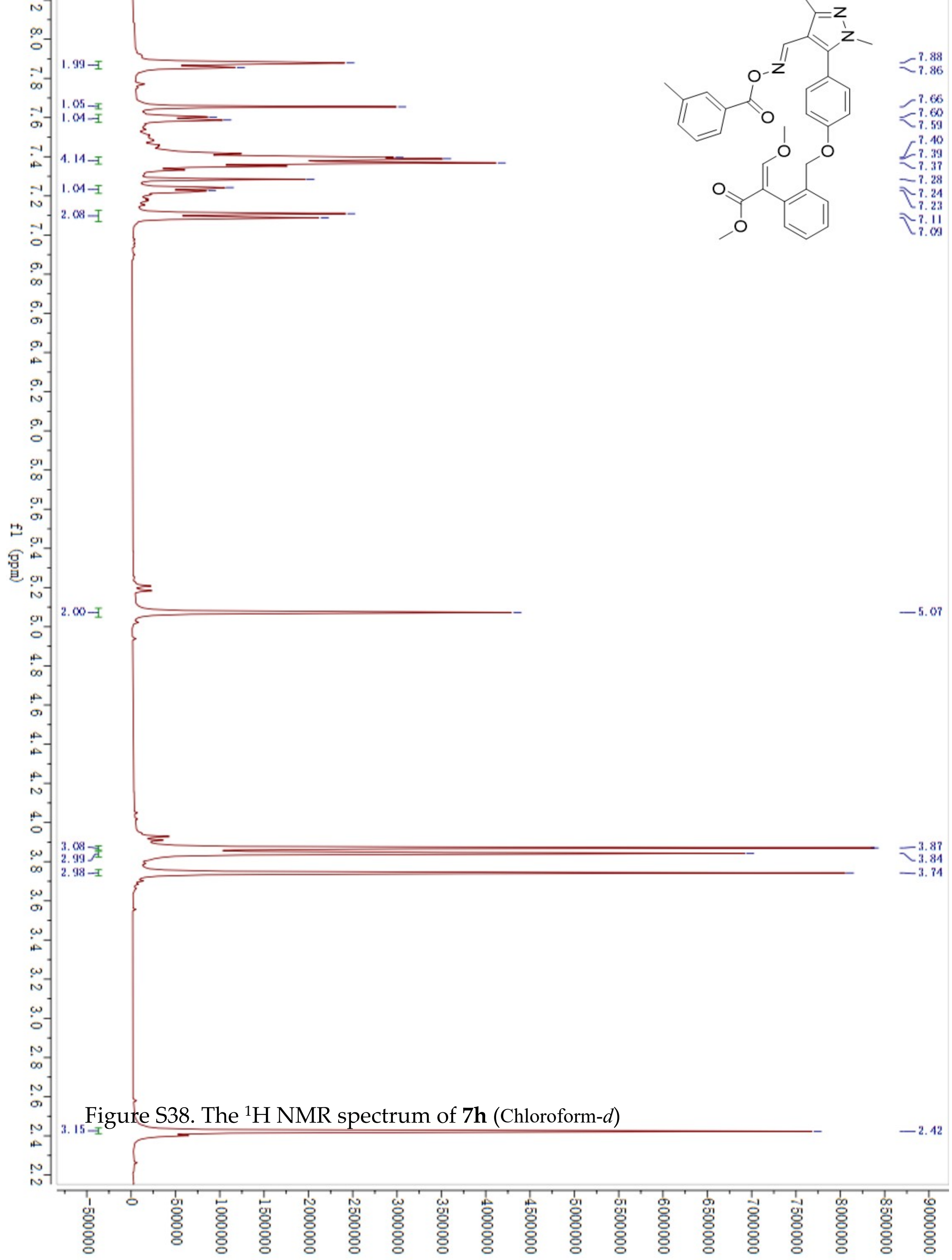

Figure S38. The ^{1}H NMR spectrum of **7h** (Chloroform-*d*)

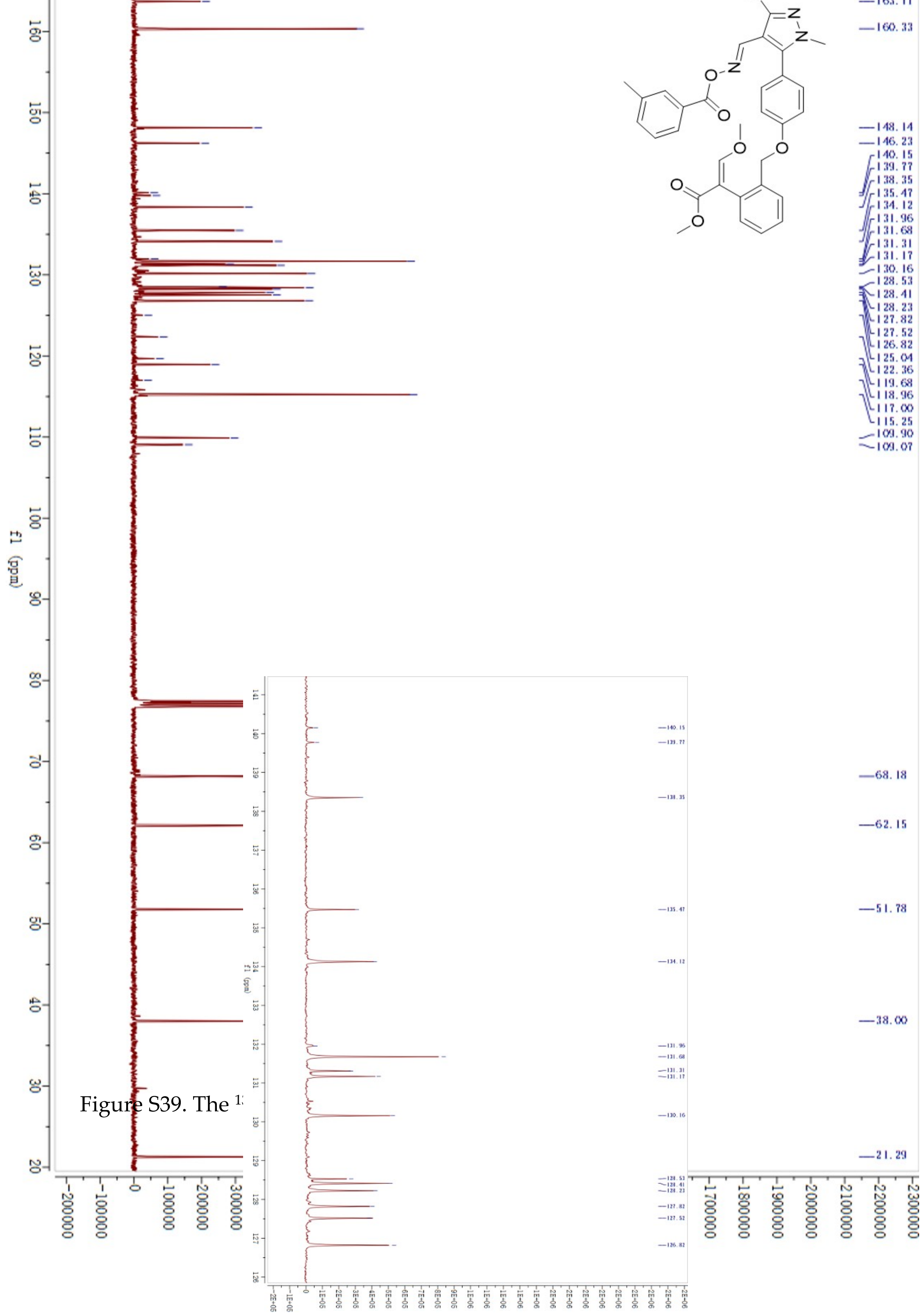

Figure S39. The [13]

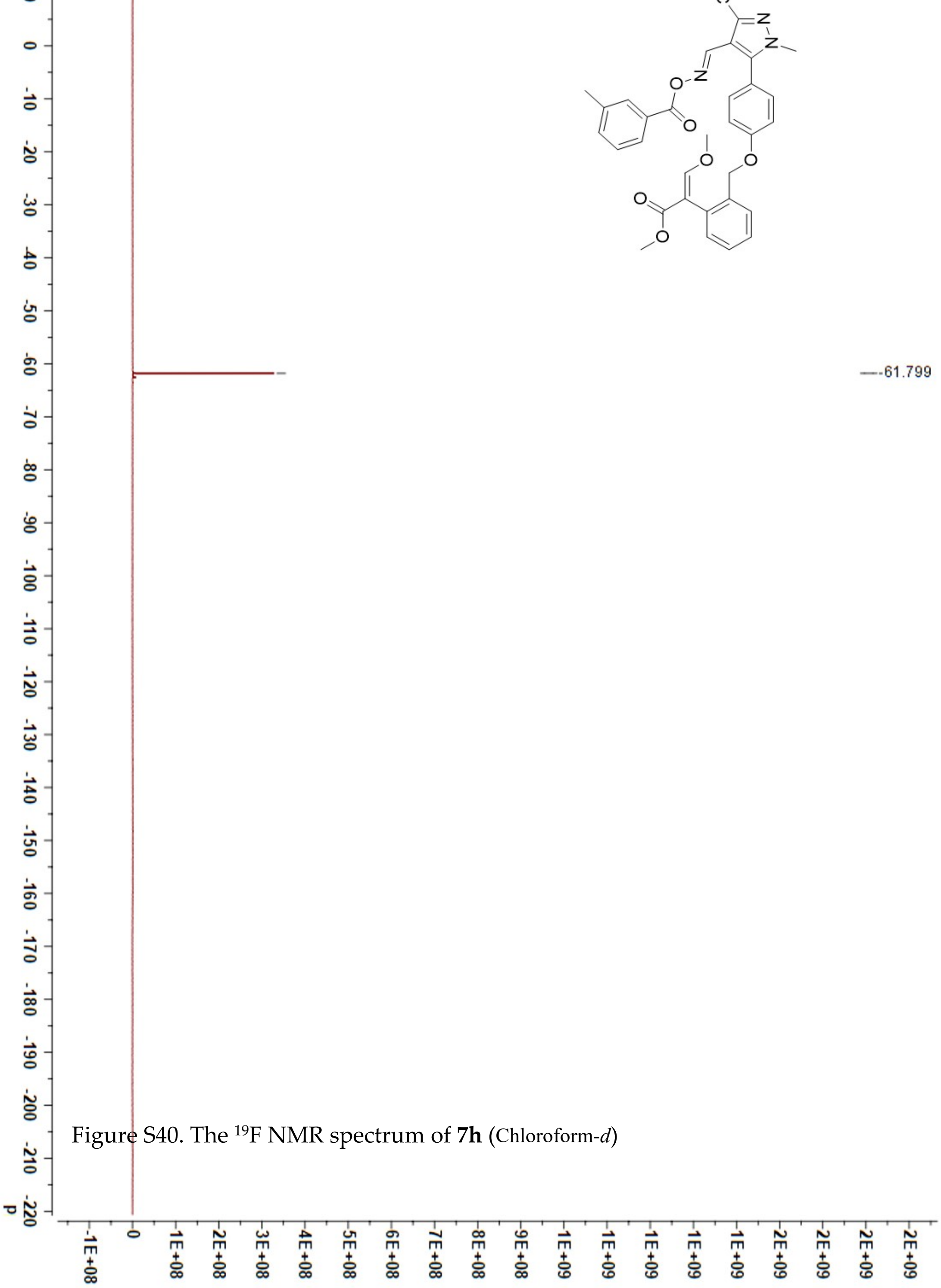

Figure S40. The ^{19}F NMR spectrum of **7h** (Chloroform-*d*)

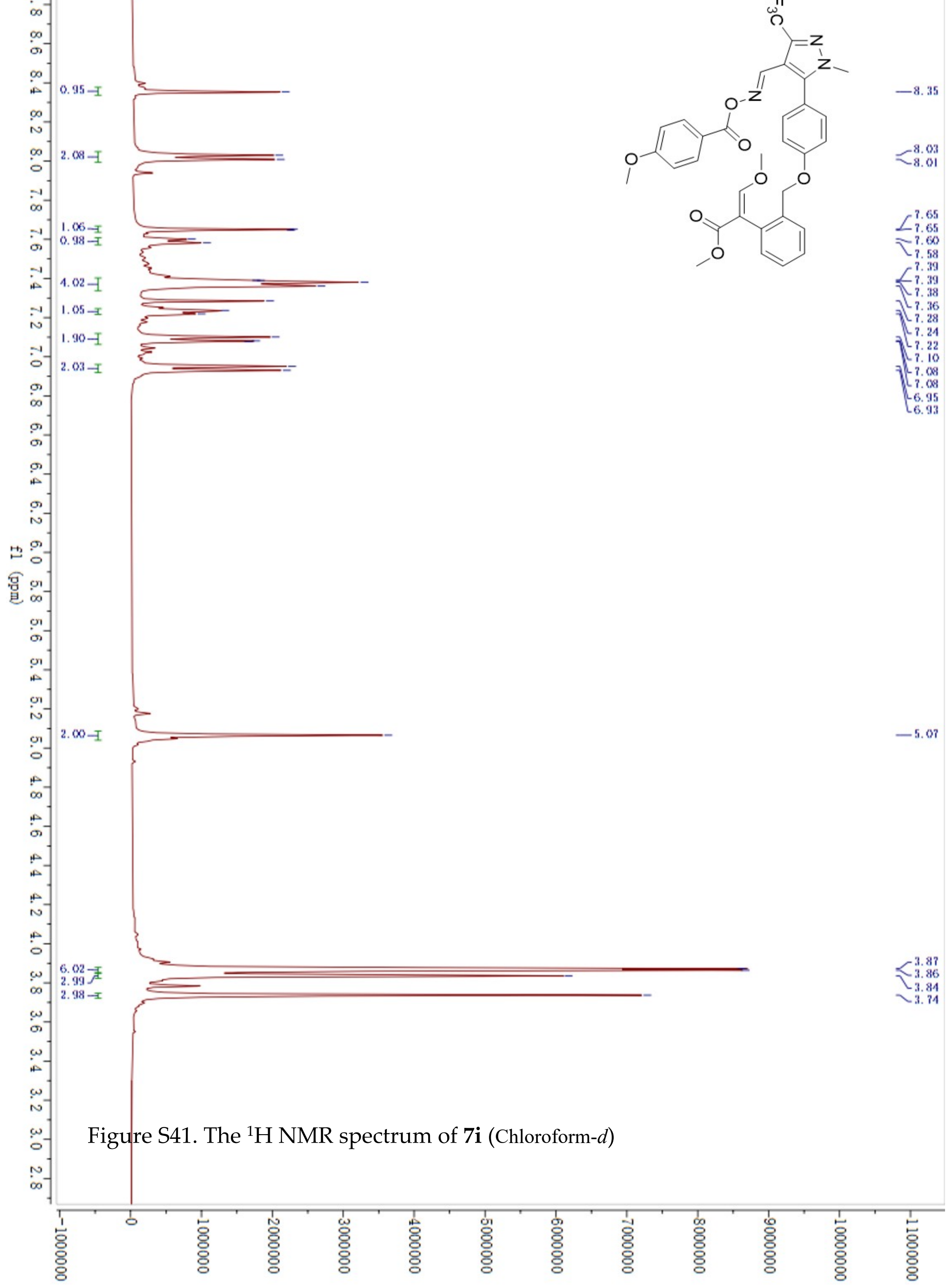

Figure S41. The ^{1}H NMR spectrum of **7i** (Chloroform-*d*)

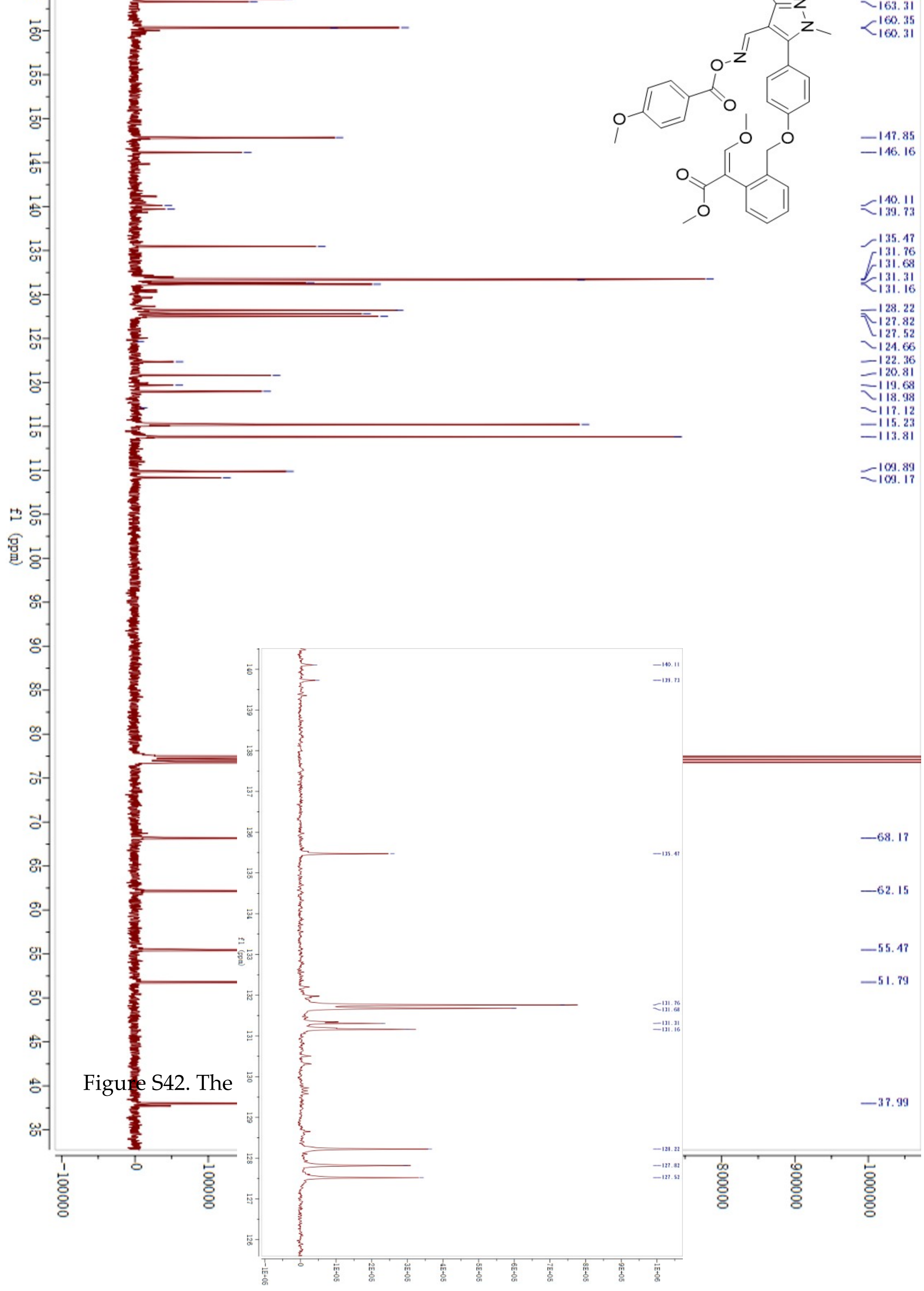

Figure S42. The

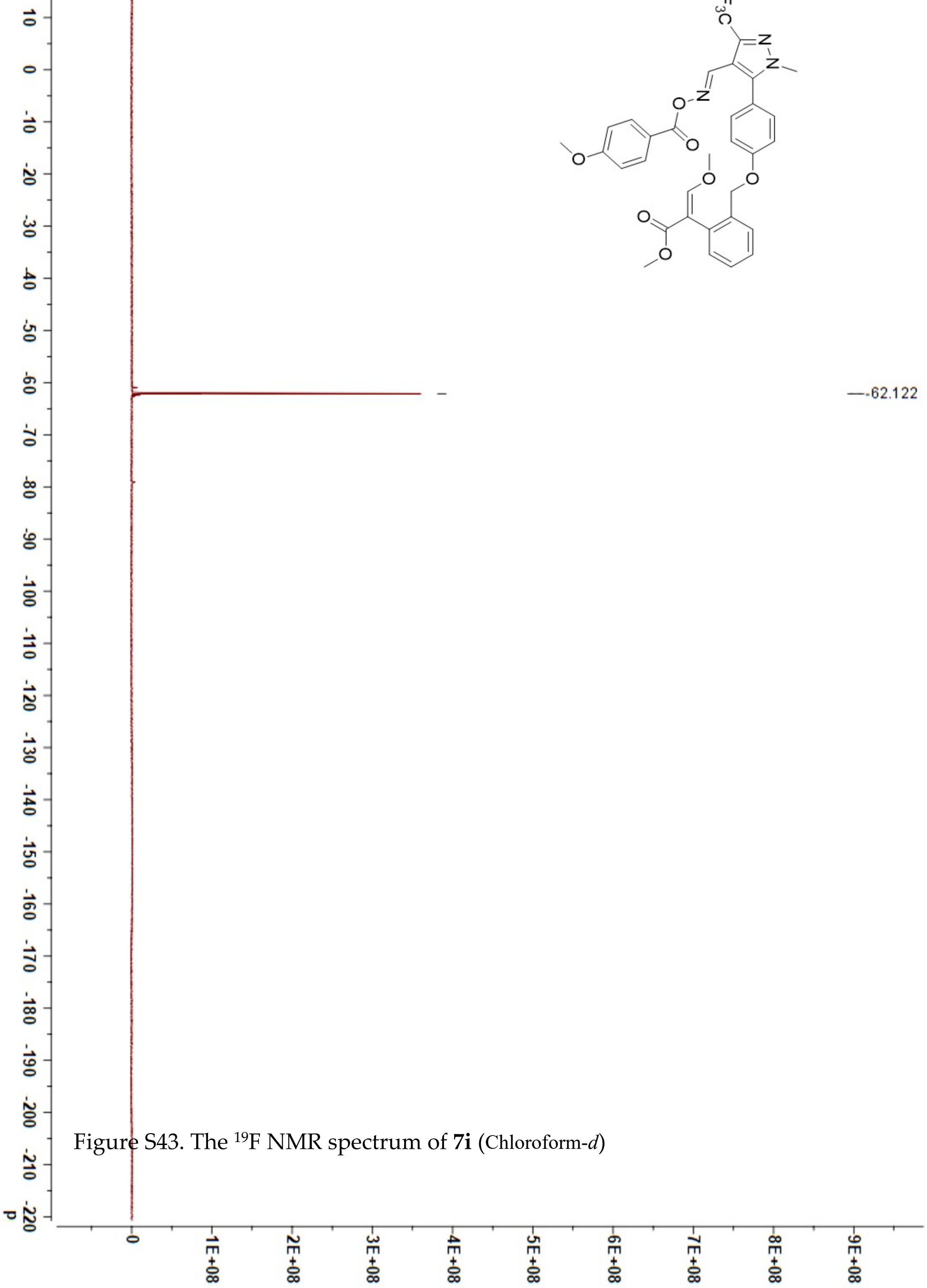

Figure S43. The ^{19}F NMR spectrum of **7i** (Chloroform-*d*)

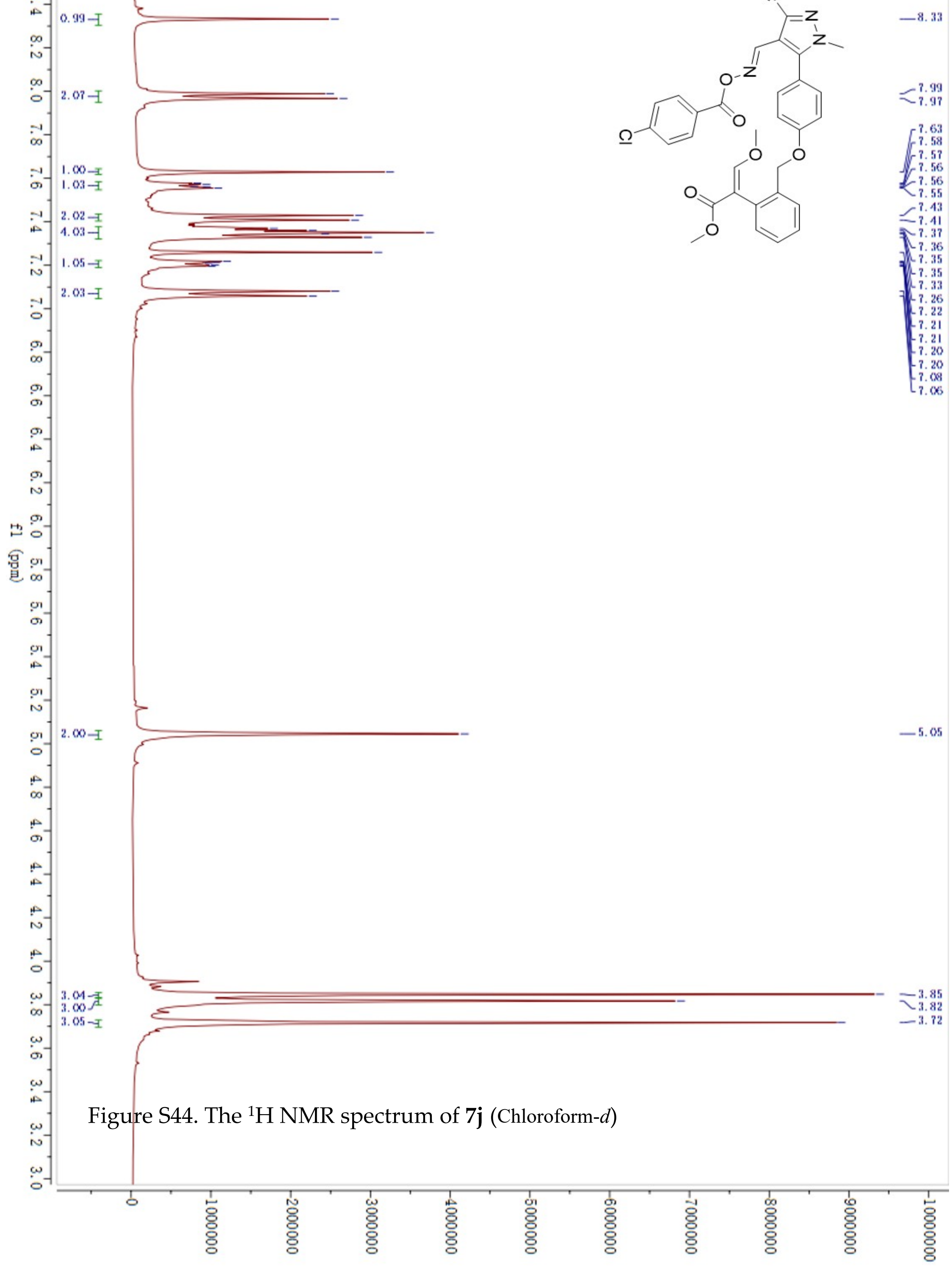

Figure S44. The ^{1}H NMR spectrum of **7j** (Chloroform-*d*)

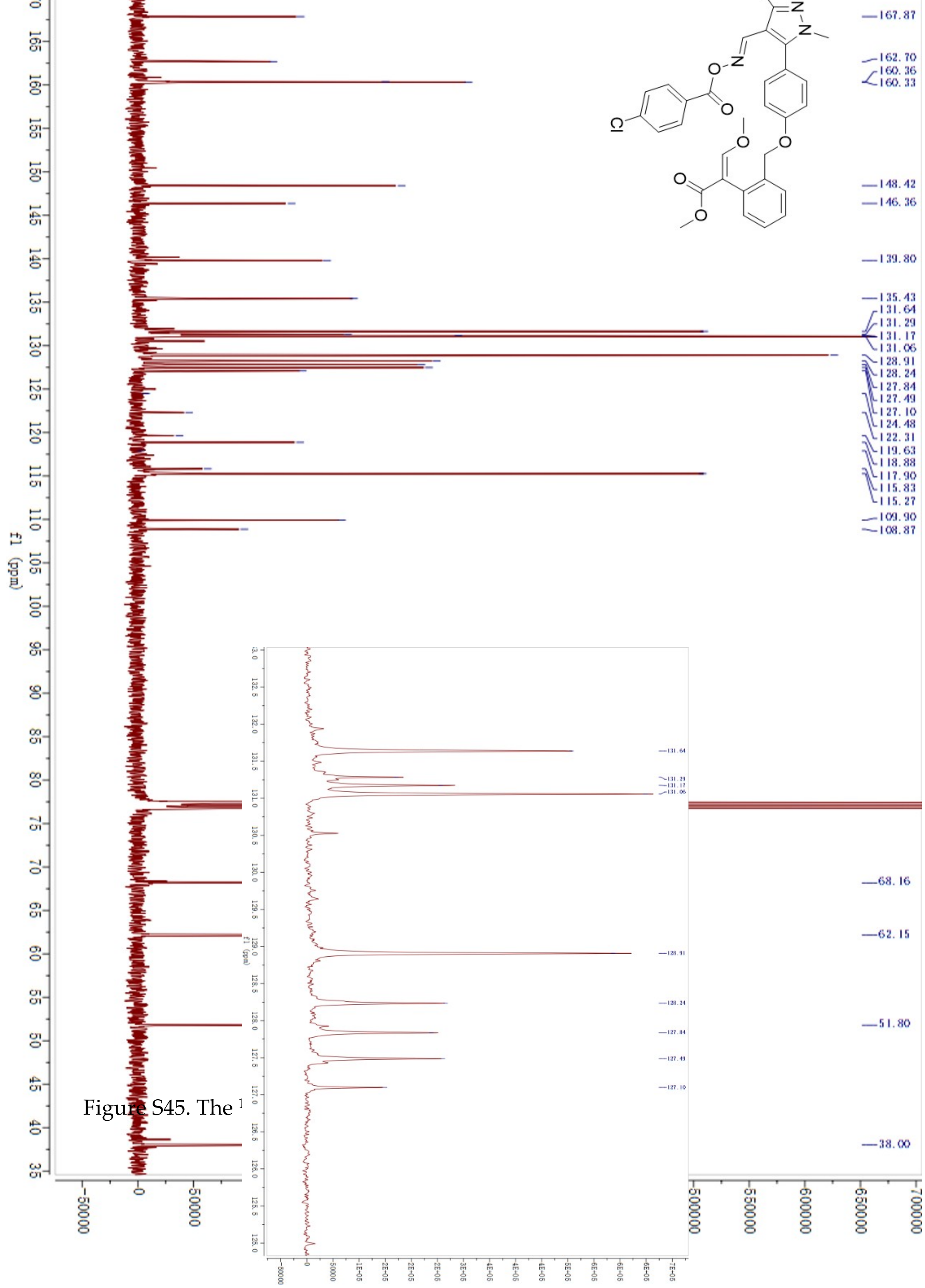

Figure S45. The [illegible]

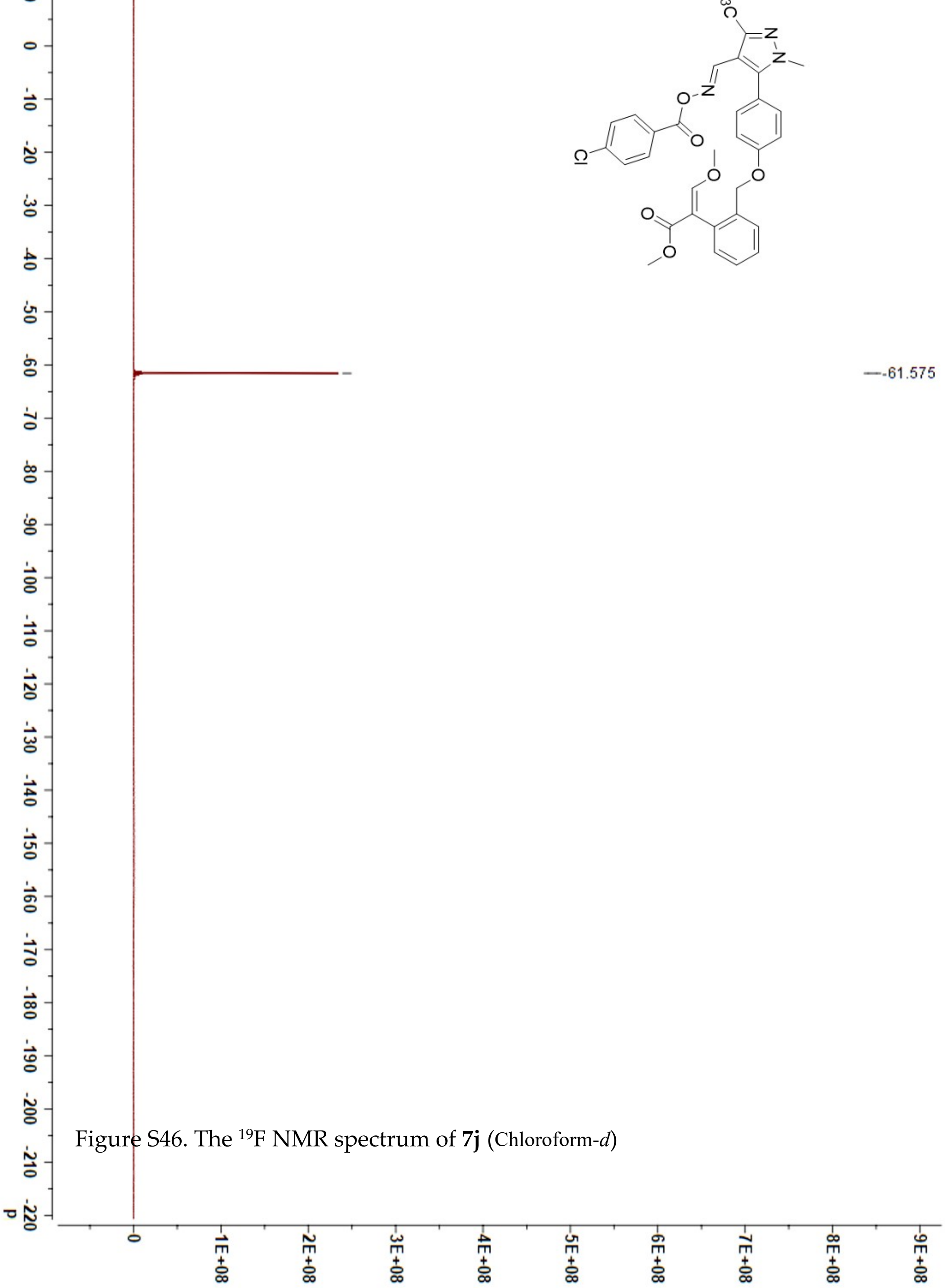

Figure S46. The ^{19}F NMR spectrum of **7j** (Chloroform-*d*)

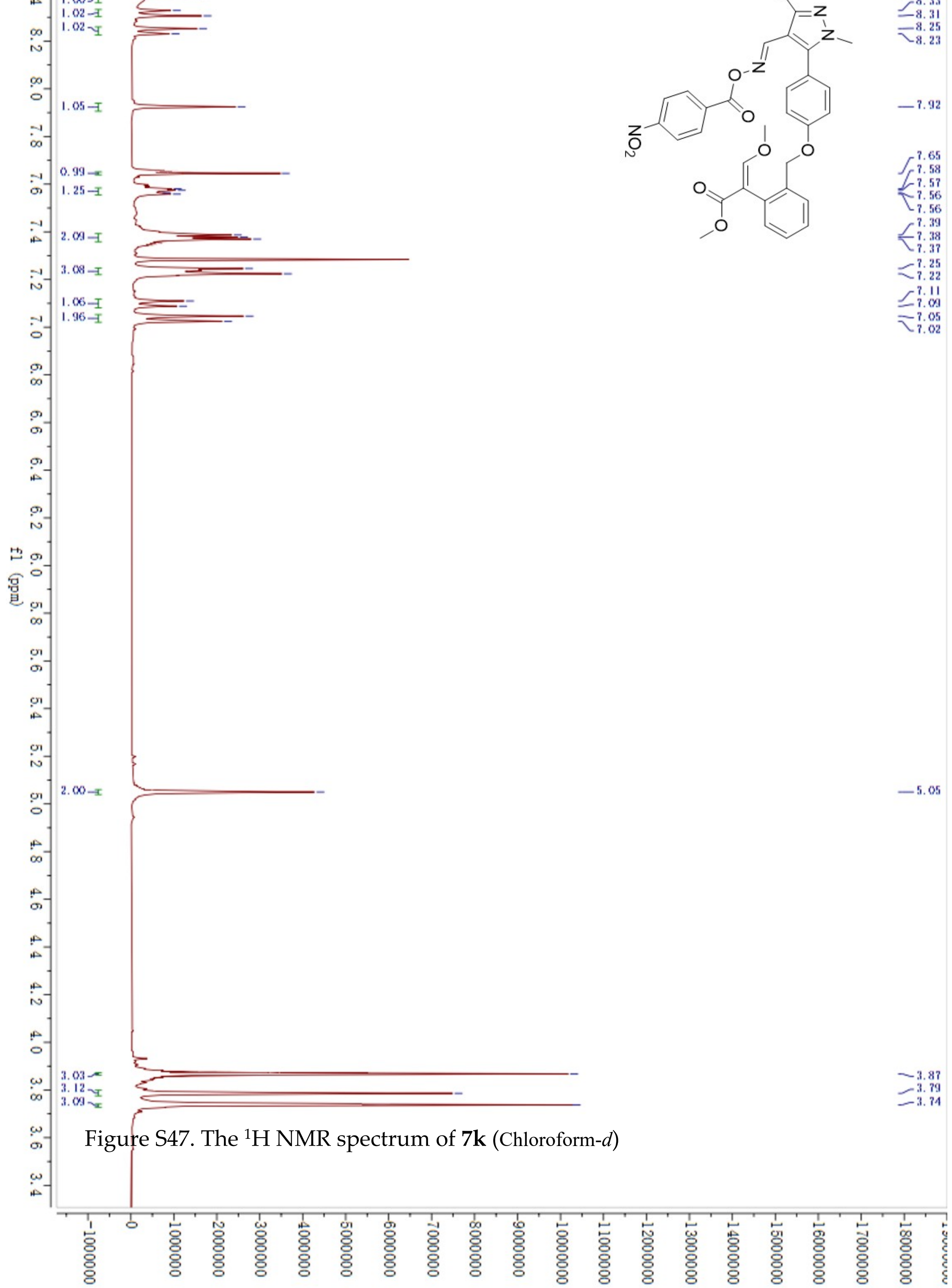

Figure S47. The ¹H NMR spectrum of **7k** (Chloroform-*d*)

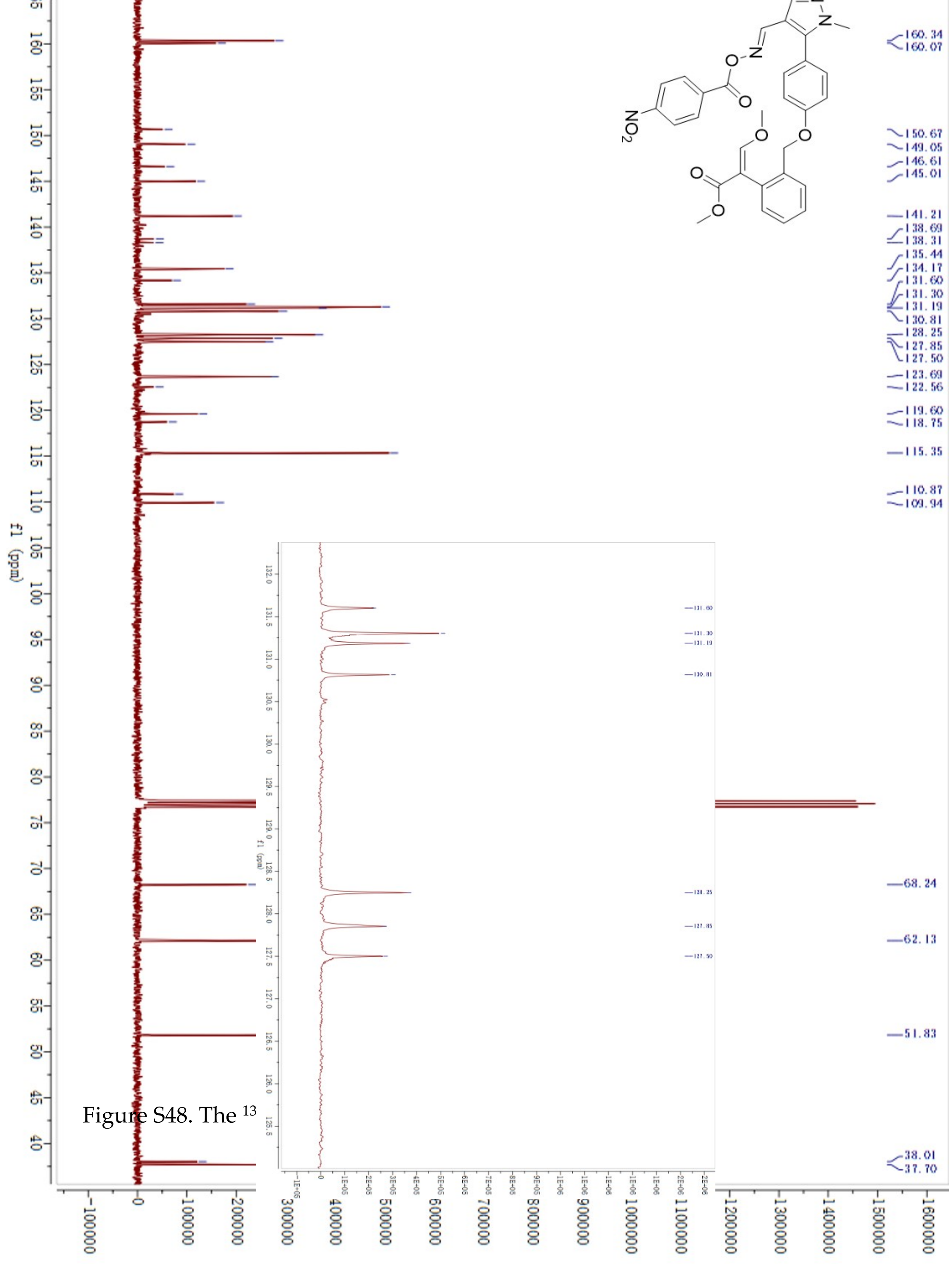

Figure S48. The [13]

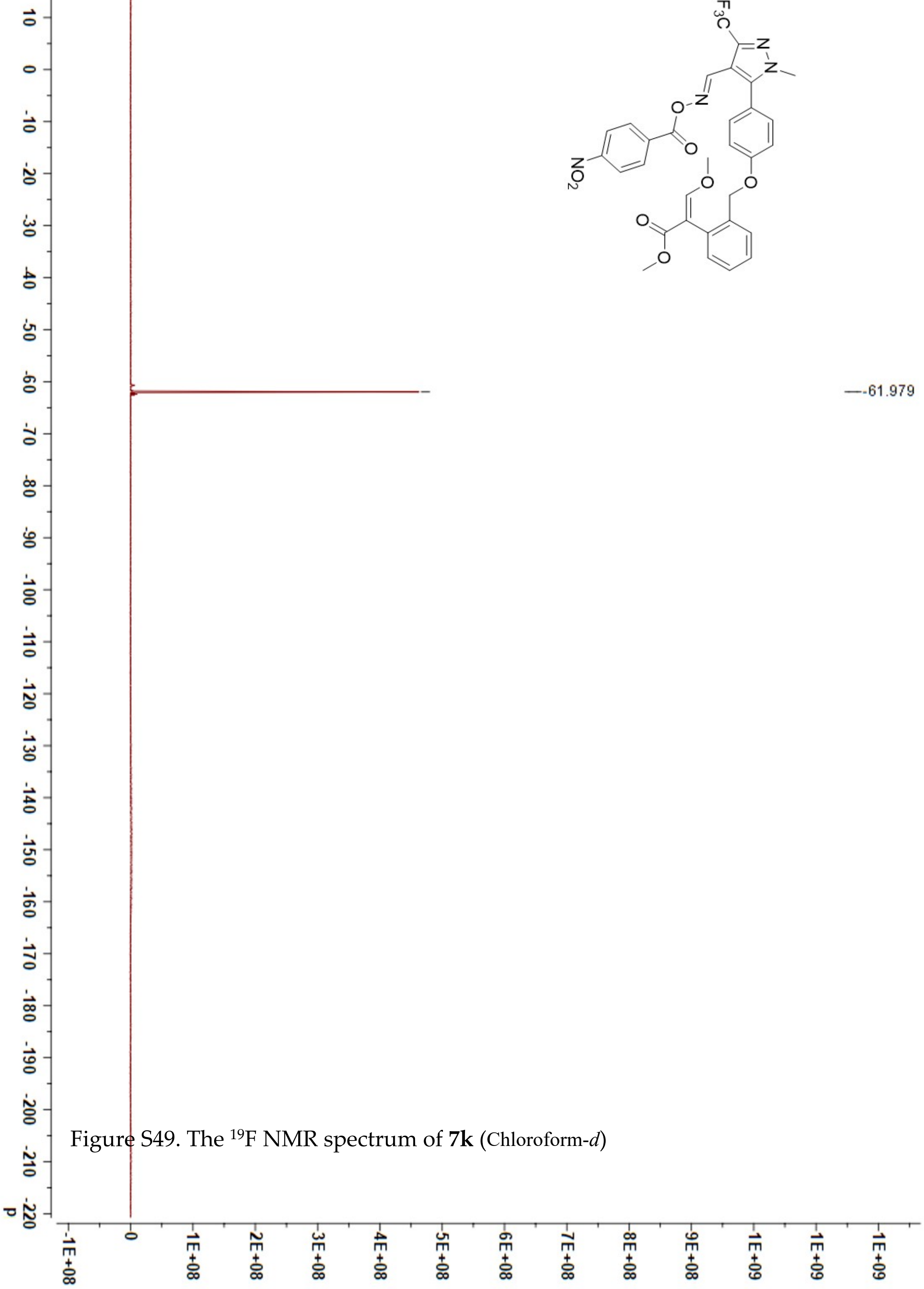

Figure S49. The ^{19}F NMR spectrum of **7k** (Chloroform-*d*)

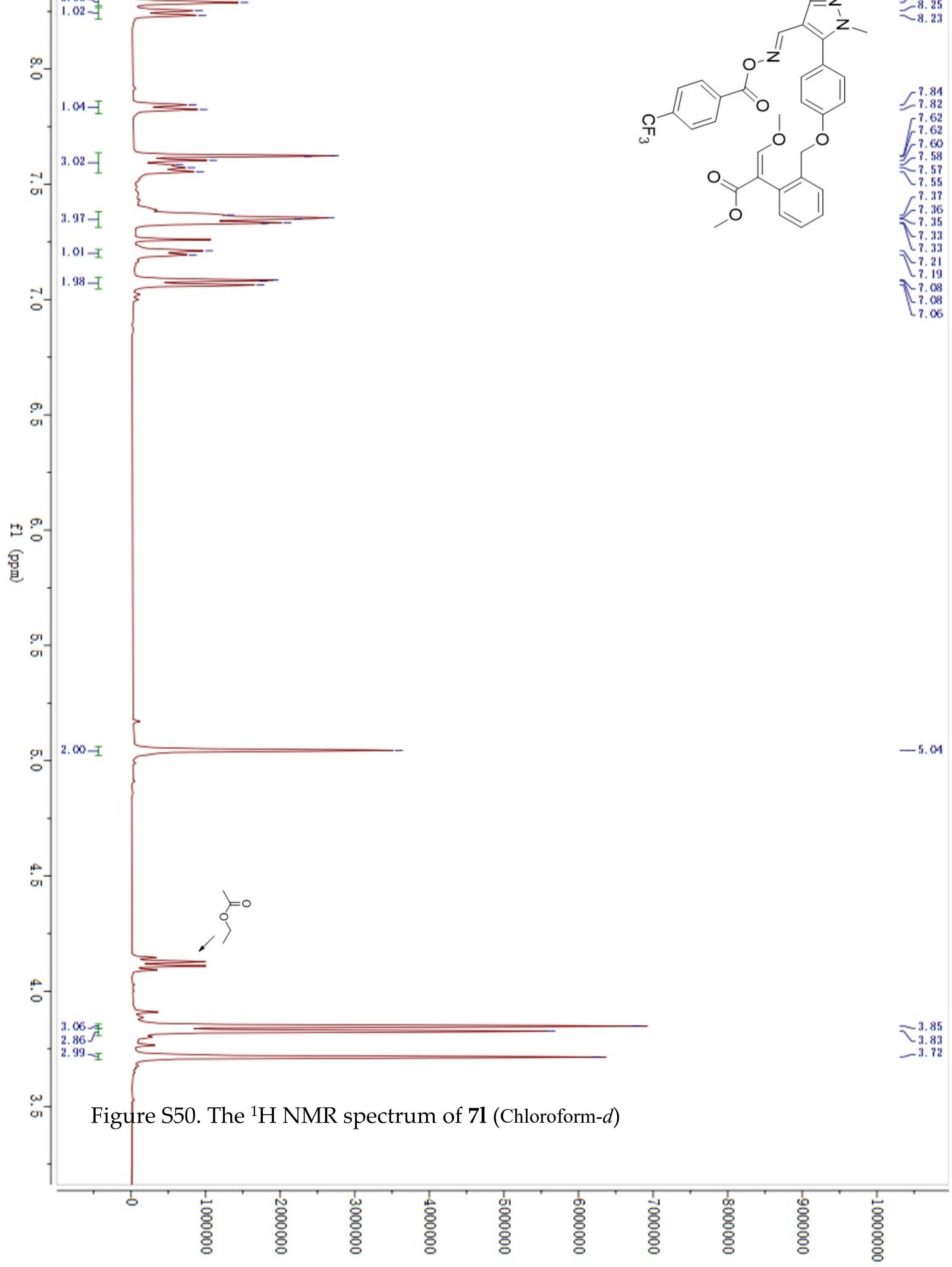

Figure S50. The ^{1}H NMR spectrum of **7l** (Chloroform-*d*)

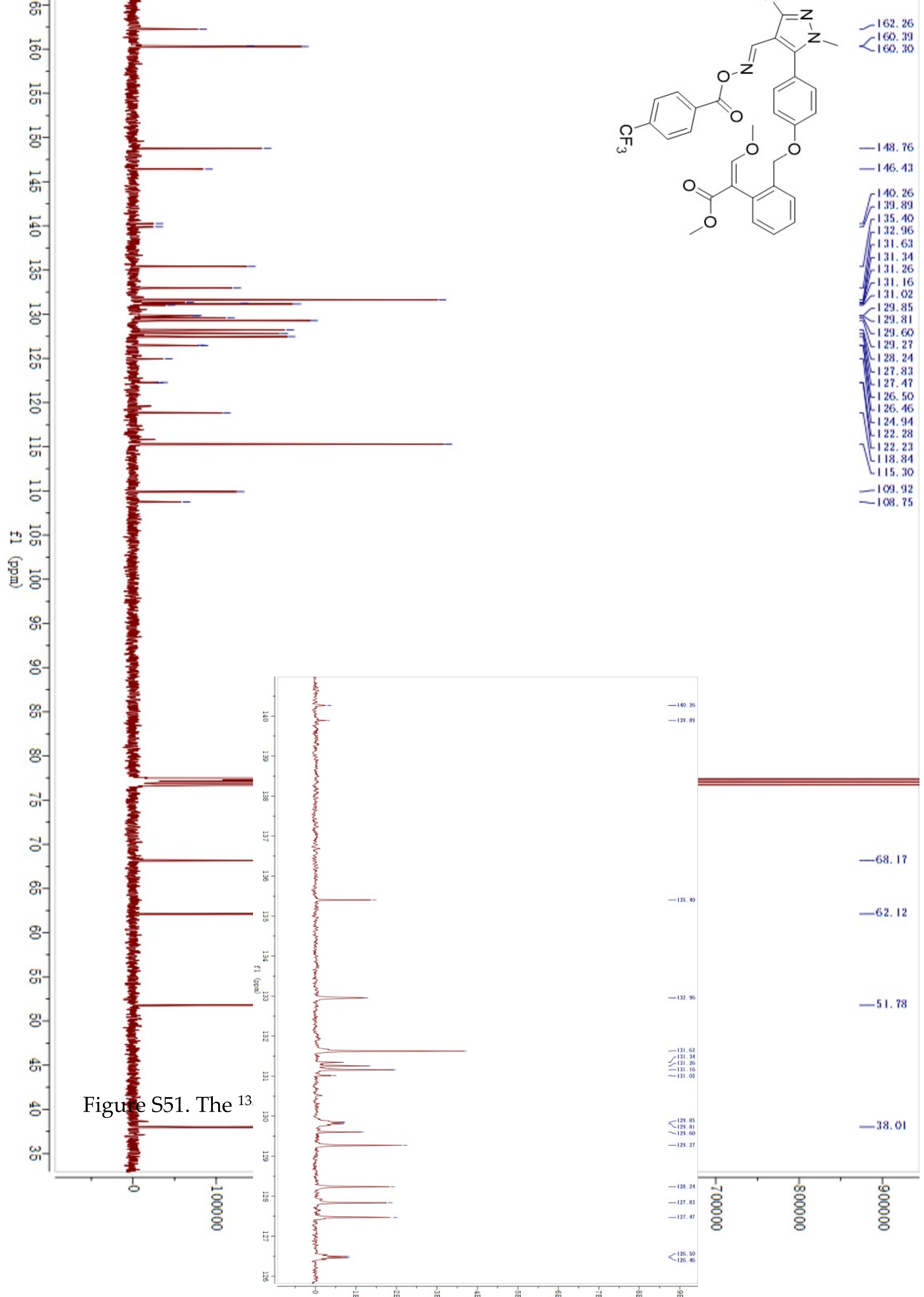

Figure S51. The [illegible]

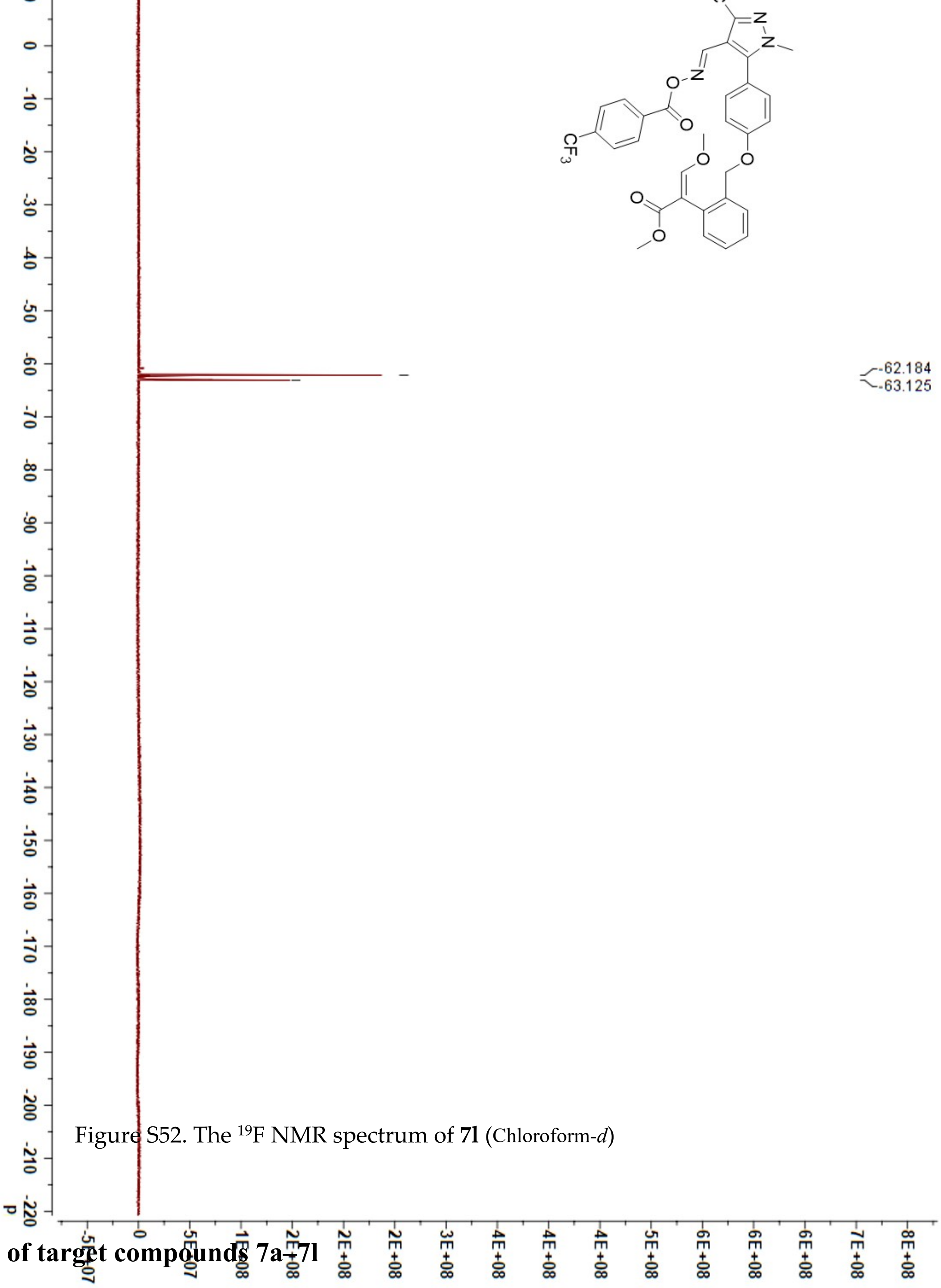

Figure S52. The ^{19}F NMR spectrum of **7l** (Chloroform-*d*)

3. IR spectroscopy of target compounds 7a–7l

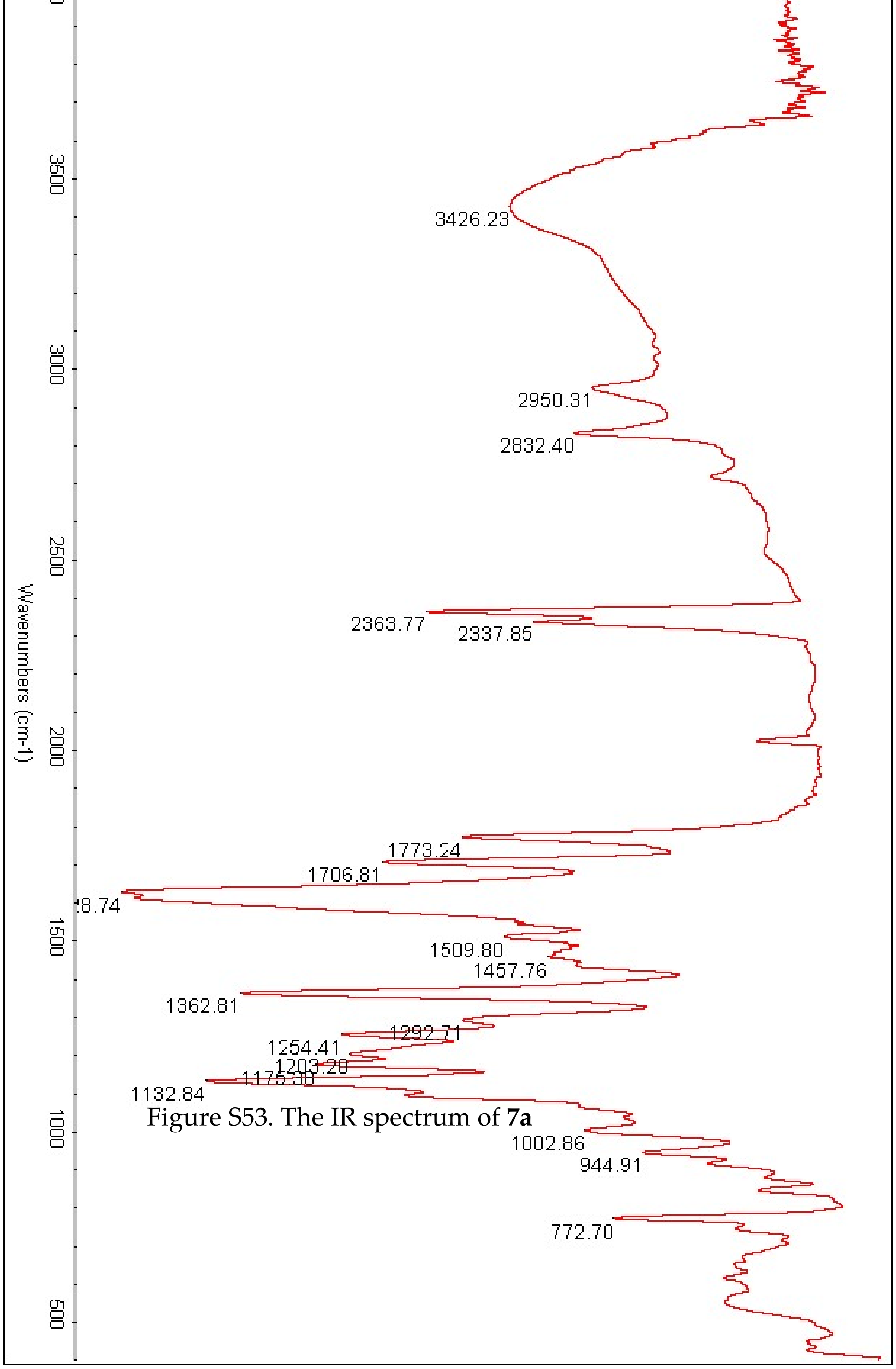

Figure S53. The IR spectrum of **7a**

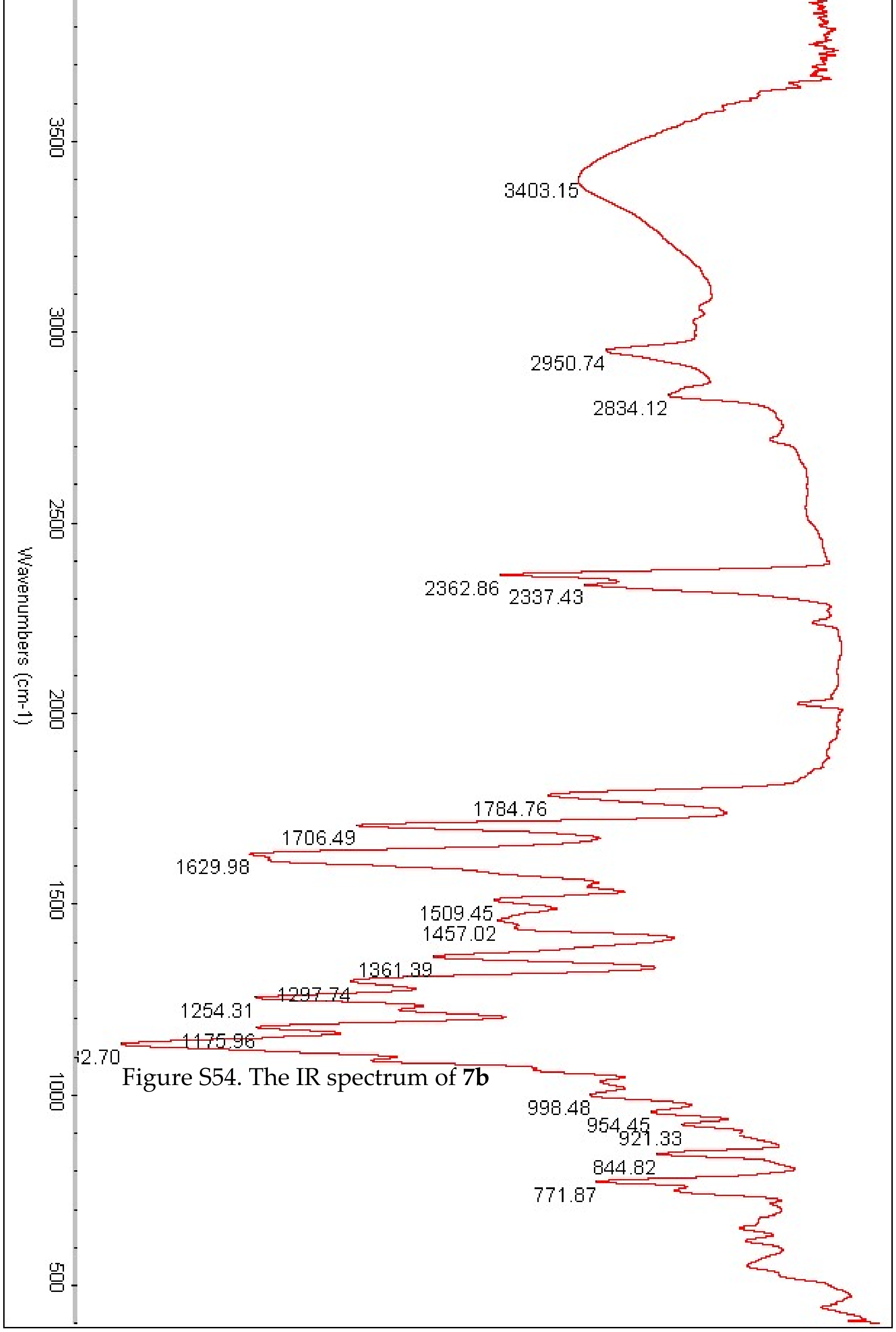

Figure S54. The IR spectrum of **7b**

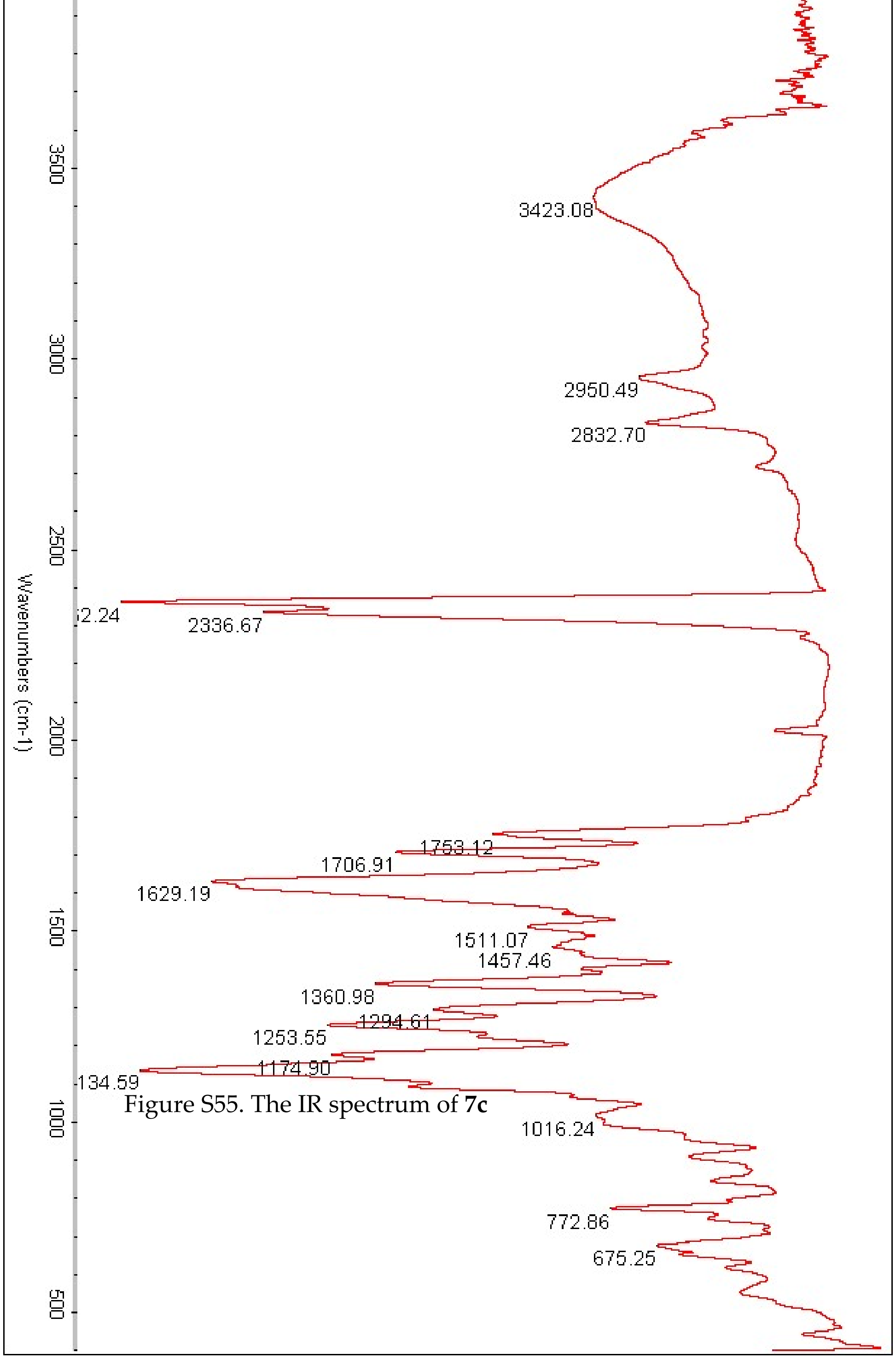

Figure S55. The IR spectrum of **7c**

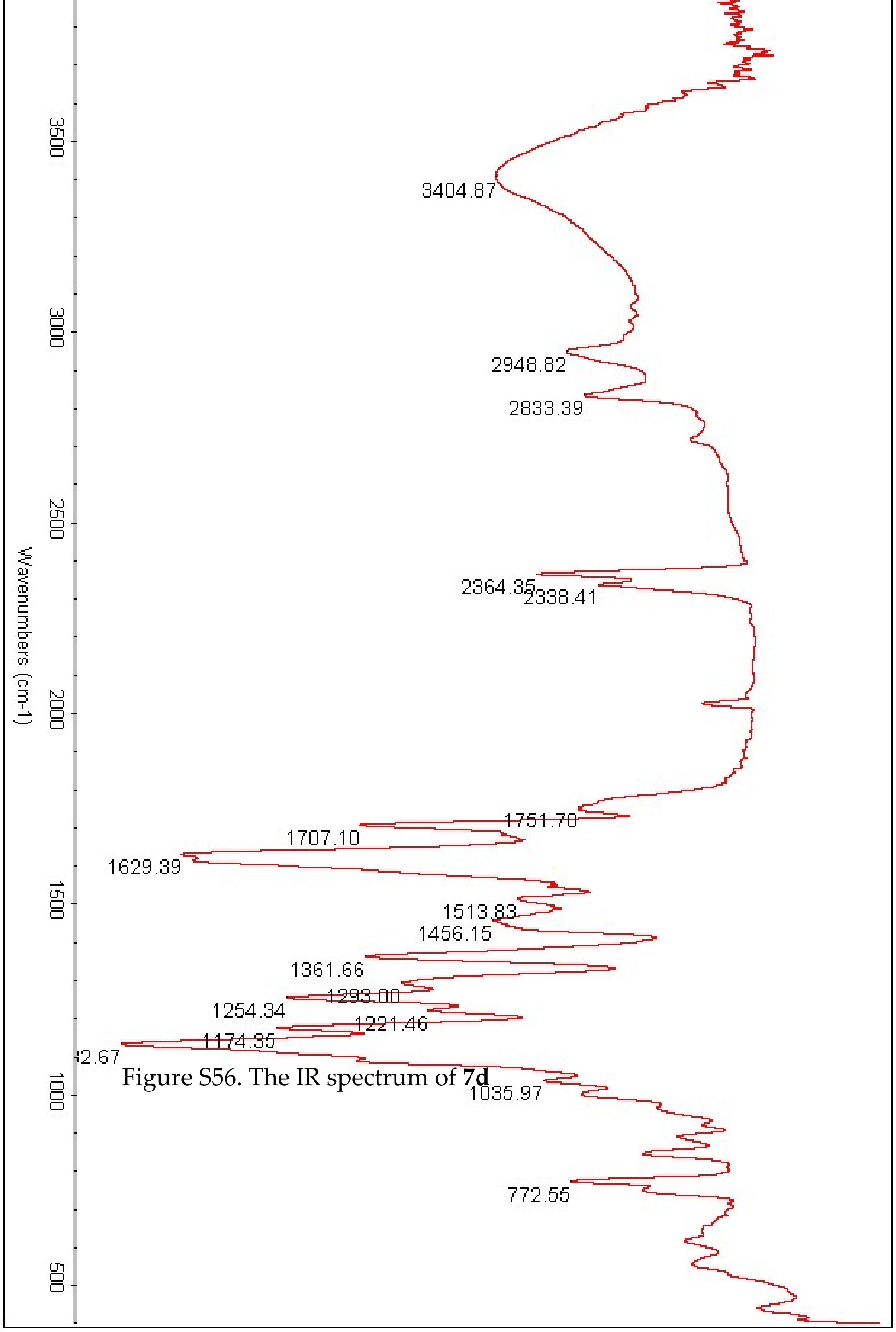

Figure S56. The IR spectrum of **7d**

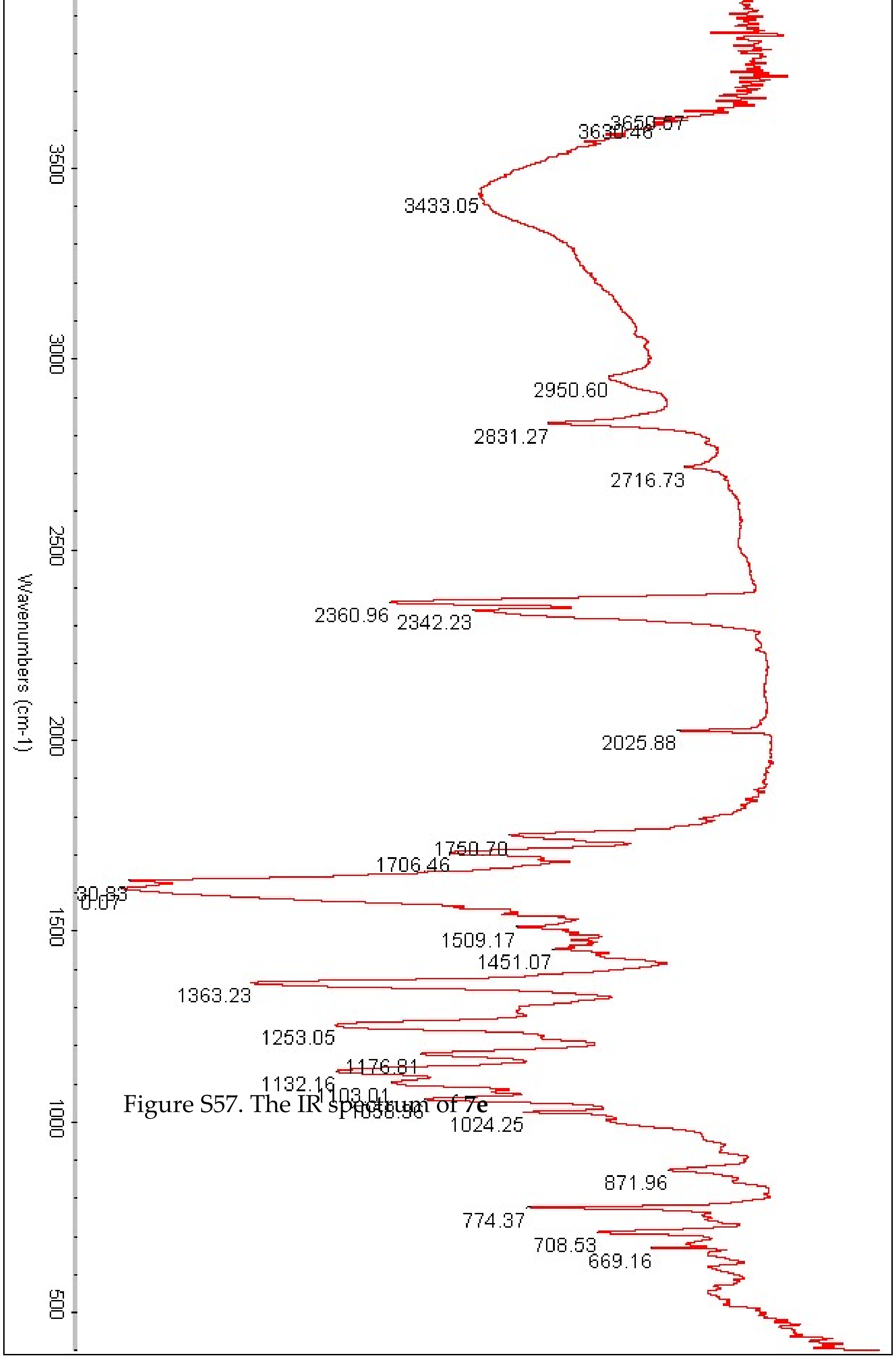

Figure S57. The IR spectrum of **7e**

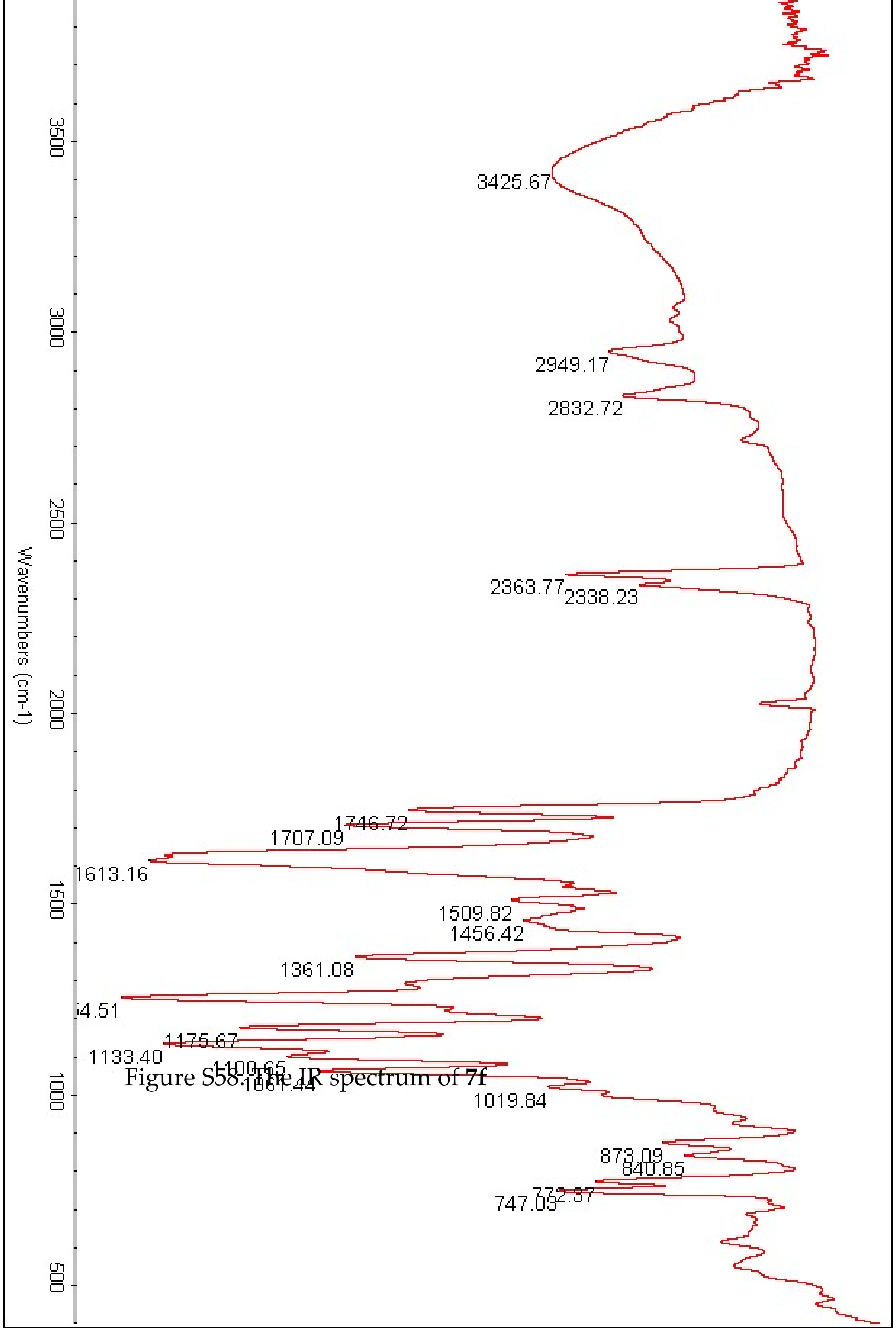

Figure S58. The IR spectrum of **7f**

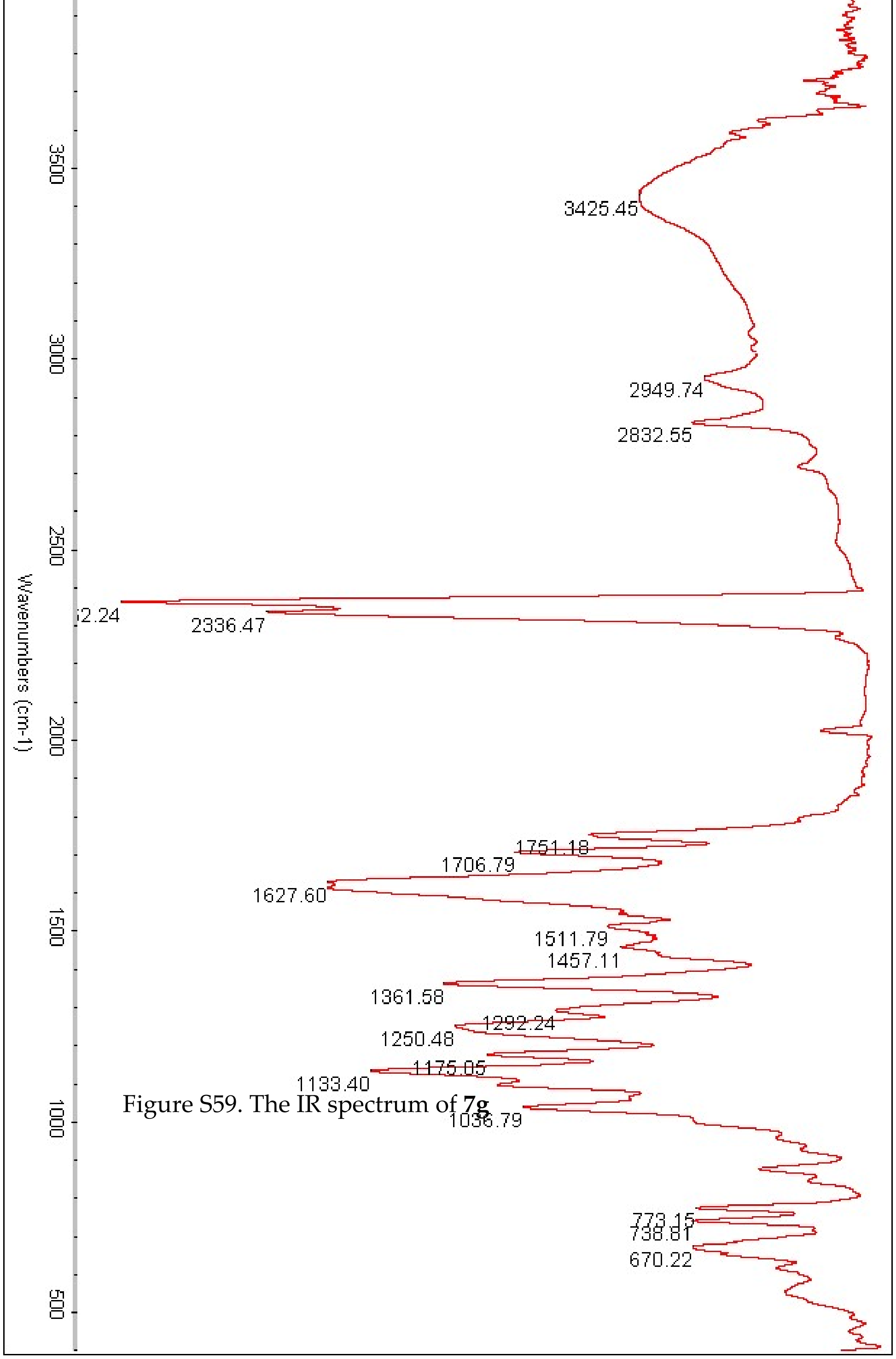

Figure S59. The IR spectrum of **7g**

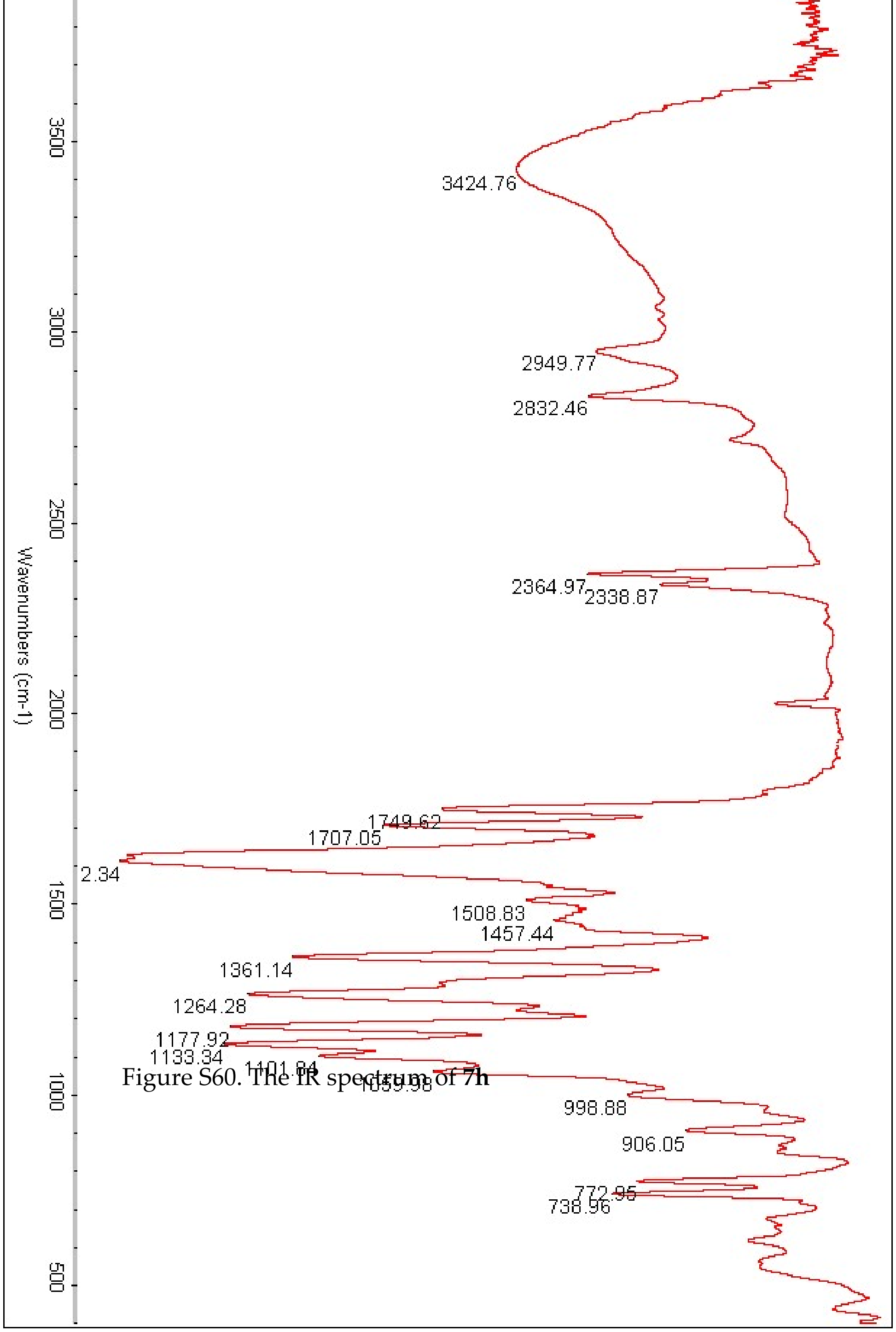

Figure S60. The IR spectrum of **7h**

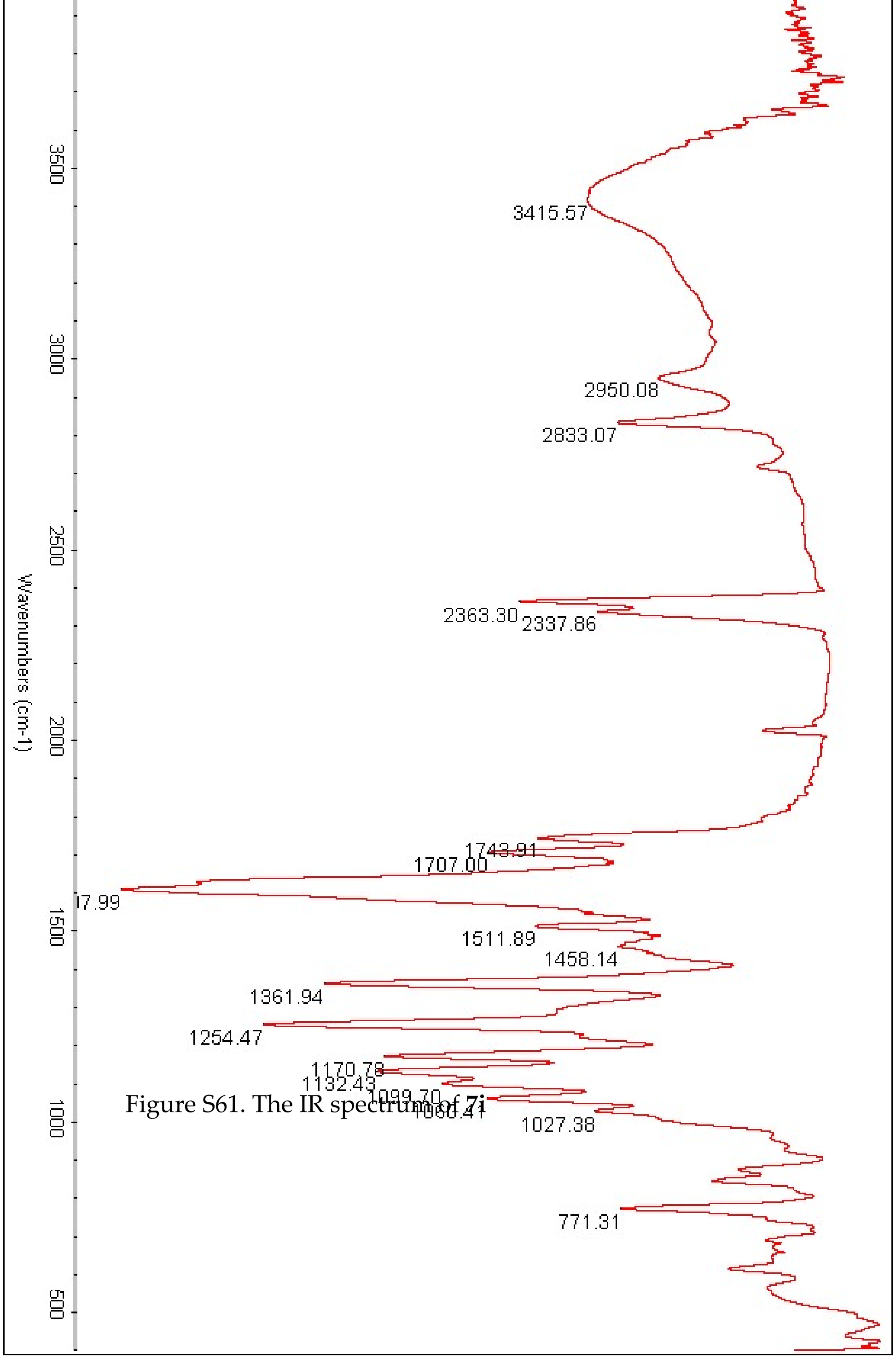

Figure S61. The IR spectrum of **7i**

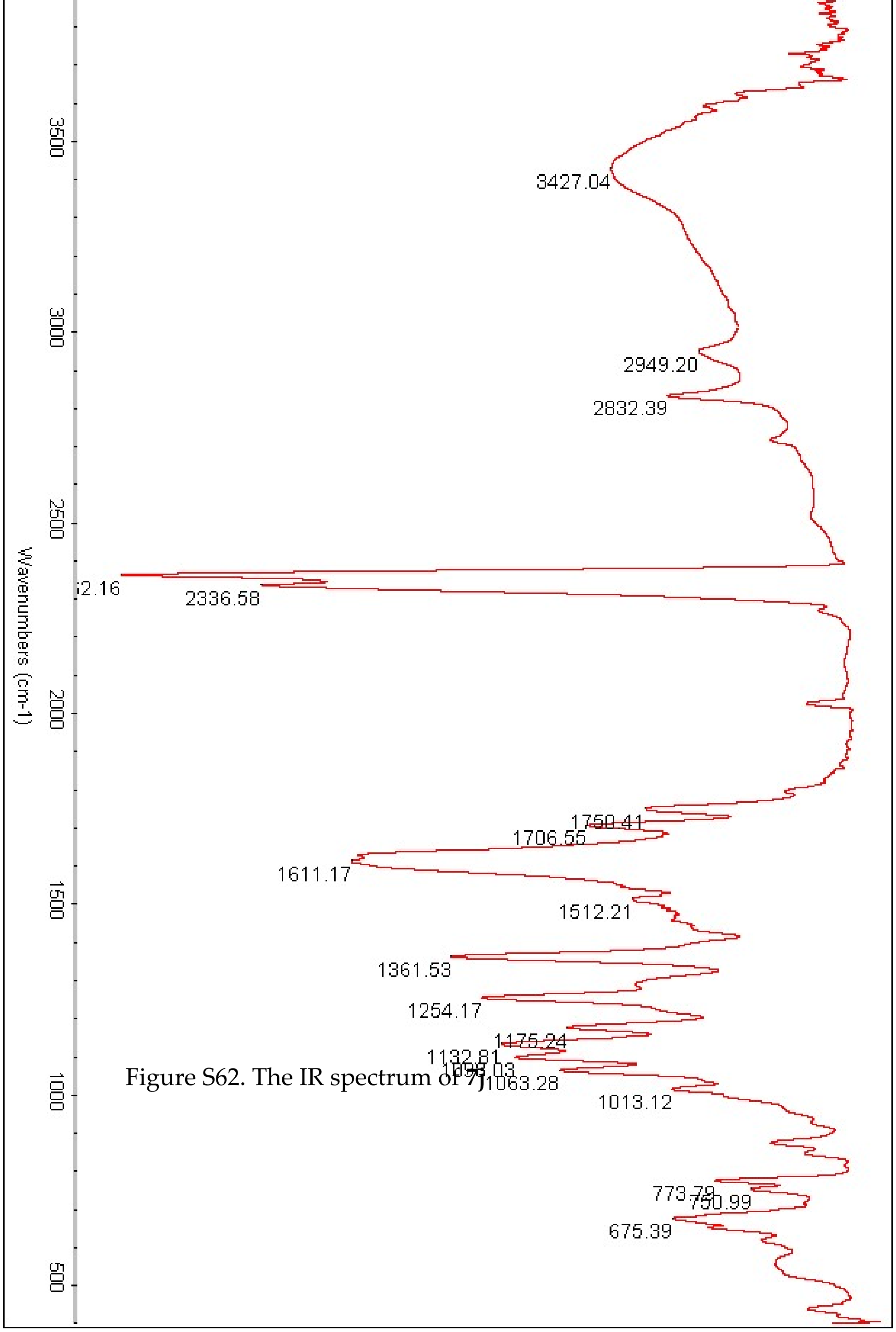

Figure S62. The IR spectrum of 7j

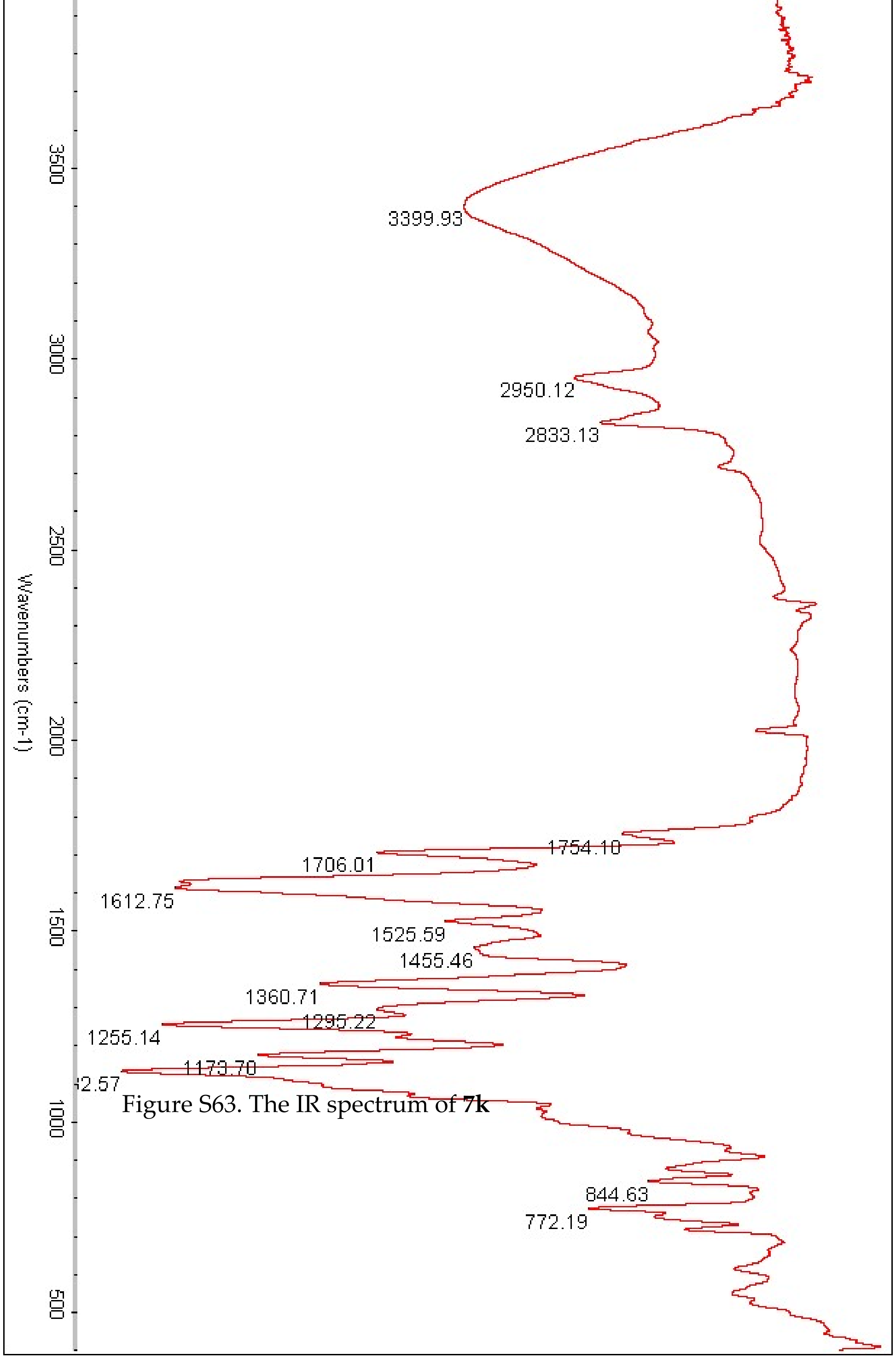

Figure S63. The IR spectrum of **7k**

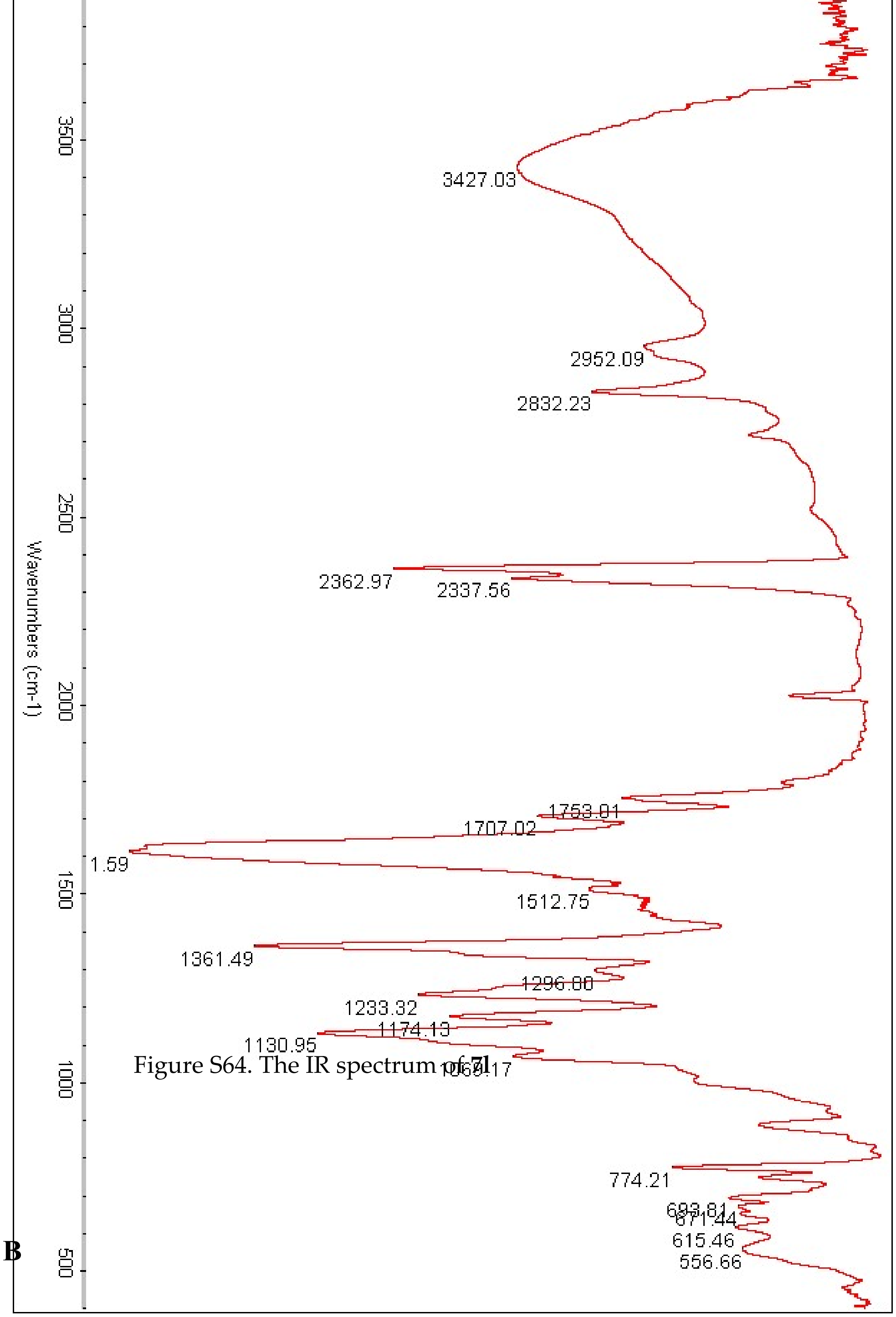

Figure S64. The IR spectrum of 7l

4.Figure A and Figure B

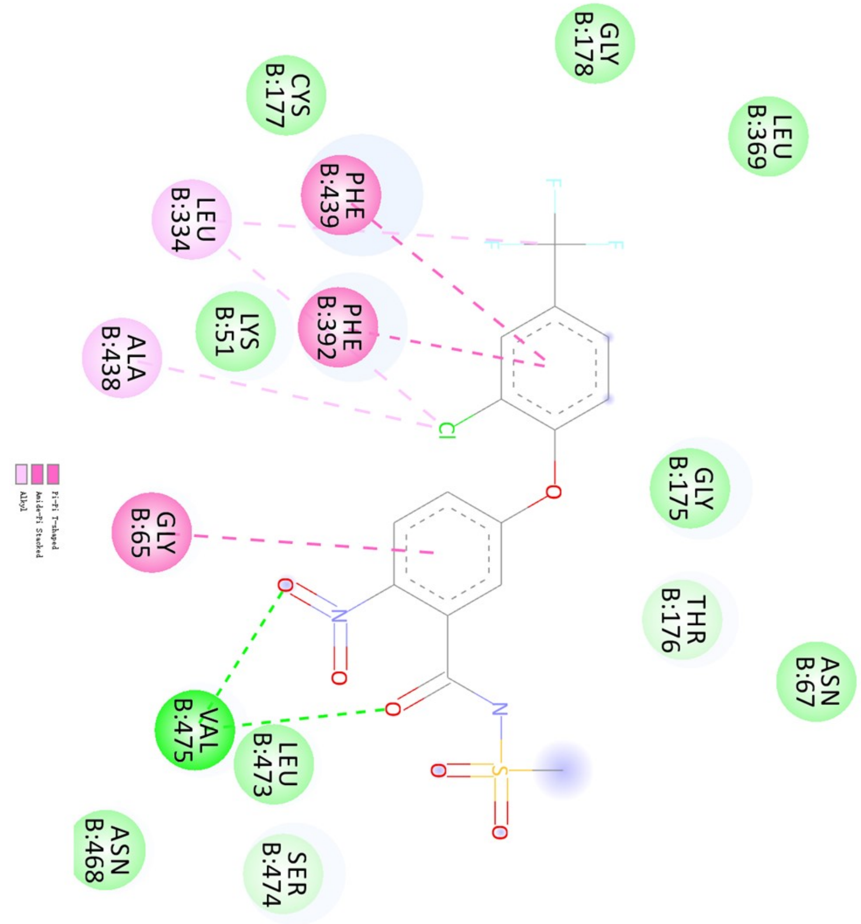

Figure S65. Two-dimensional diagram of the interaction between fomesafen and protoporphyrinogen oxidase

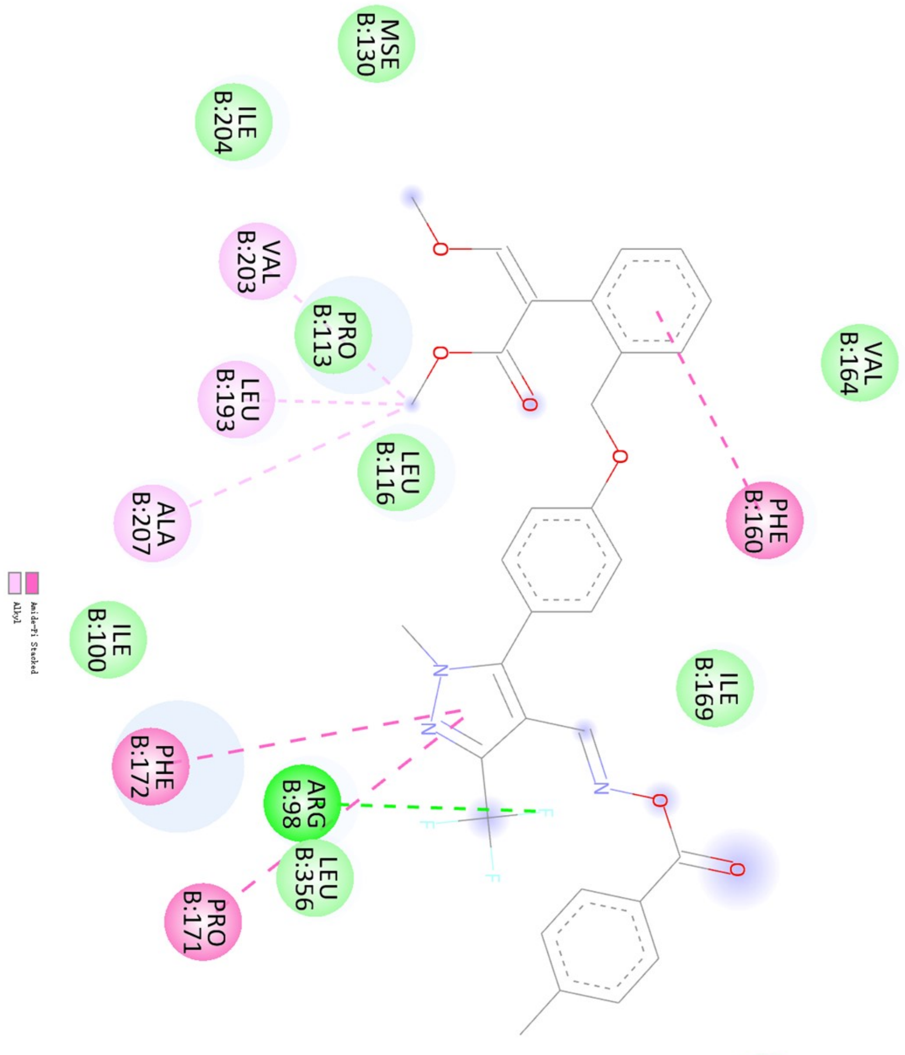

Figure S66. Two-dimensional diagram of the interaction between compound **7f** and protoporphyrinogen oxidase
